# Feedstock‐Derived Thiophene Radical Anions Exhibiting Redox Stability at Extreme Potentials

**DOI:** 10.1002/anie.202512271

**Published:** 2025-07-23

**Authors:** Aurelio C. Gasser, Daniel Käch, Máté J. Bezdek

**Affiliations:** ^1^ Department of Chemistry and Applied Biosciences ETH Zürich Vladimir‐Prelog‐Weg 1 Zürich 8093 Switzerland

**Keywords:** Electronic structure, Molecular electrochemistry, Radicals, Redox chemistry, Thiophene

## Abstract

Organic radicals that are both readily synthesized from commodity chemicals and stable at extreme redox potentials are rare, yet highly desirable for applications in energy storage and organic electronics. Herein, we report feedstock‐derived thiophene radical anions bearing ester functionalities that undergo galvanostatic charge–discharge redox cycling at potentials below −2 V versus Fc/Fc^+^ (Fc = ferrocene). Systematic structural modification led to the identification of a derivative exhibiting exceptional redox stability, showing promise as a scalable and low‐cost electrolyte for electron storage applications. Further, a crystalline thiophene radical anion was isolated and characterized using structural, spectroscopic, and computational methods. These studies revealed that the ester functionalities stabilize the reduced “quinoidal” thiophene electronic structure without the need for extended π‐delocalization. Taken together, a new class of electron storage media is reported that combine redox stability at extreme potentials with straightforward synthesis, while offering rare insight into the structural and electronic features of stable thiophene radicals.

## Graphical abstract

Although organic redox systems are emerging as next‐generation energy storage media, stability at high cell voltage is often achieved at the expense of synthetic accessibility. Here, we isolate and characterize a feedstock‐derived thiophene radical anion bearing simple ester groups. Despite a minimalist design, the radical and its derivatives are stable at extreme potentials, enabling their use as readily accessible electrolytes for electron storage.

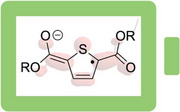

Organic redox systems that are readily accessed from low‐cost feedstocks and exhibit robust electron transfer reactivity hold promise for a host of applications spanning energy storage, catalysis, and organic electronics.^[^ ^1^, ^2^, ^3^, ^4^, ^5^, ^6^ ^]^ However, owing to the inherent reactivity of organic molecules upon reduction or oxidation, identifying scaffolds bearing unpaired electrons with long‐term stability remains a major challenge.^[^ ^7^, ^8^, ^9^ ^]^ This is especially pronounced in the development of chargeable organic electrolytes for emerging energy storage technologies such as redox flow batteries (RFBs), where redox stability at high cell voltage, low molecular weight, and straightforward synthesis are essential yet often mutually exclusive design goals.^[^ ^10^ ^]^ Addressing the prevailing tradeoff between redox stability at extreme potentials and synthetic complexity will be key to unlocking the full promise of organic systems for energy storage and motivates the search for readily‐accessible organic radicals.^[^ ^11^, ^12^, ^13^ ^]^

Oligothiophenes are a promising platform in this context due to their direct accessibility from feedstock chemicals, and tunable optoelectronic properties.^[^ ^14^, ^15^, ^16^, ^17^ ^]^ Previous work on the redox reactivity of oligothiophenes has mainly focused on oxidative electron transfer to induce polymerization and generate conductive (p‐type) materials (Scheme 1a).^[^ ^18^, ^19^, ^20^, ^21^, ^22^ ^]^ By contrast, the reductive (n‐type) redox chemistry of oligothiophenes is far less explored, largely due to the extreme potentials required for reduction and the inherent instability of the resulting species.^[^ ^23^ ^]^ On this front, access to well‐defined reduced oligothiophenes would provide electronic and structural insights and may open new charge transport mechanisms for this ubiquitous class of conducting materials.^[^ ^24^, ^25^ ^]^ Further, owing to their extreme reduction potentials, reductively stable oligothiophenes may be applied as high cell voltage electrolytes for nonaqueous RFBs.^[^ ^2^, ^26^, ^27^, ^28^ ^]^ Despite these prospects, oligothiophenes remain exceptionally challenging to study under reducing conditions.

**Scheme 1:**
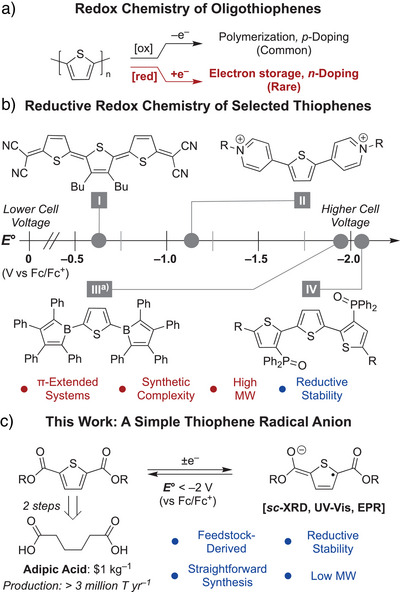
a) Summary of the redox processes in oligothiophenes. b) Selected examples of functionalized oligothiophenes. c) The design and advantages of the system reported in this work. ^a)^ *E*° estimated from chemical reactivity due to the irreversibility of electrochemical features.

One approach for studying the reductive chemistry of oligothiophenes involves the installation of stabilizing functionalities in soluble small molecule analogs. Shown in Scheme 1b, previous examples include thiophenes and terthiophenes containing dicyanomethylene (**I**),^[^ ^29^ ^]^ viologen (**II**),^[^ ^30^ ^]^ and borolyl (**III**)^[^ ^31^ ^]^ substituents, which exhibit varying degrees of stability under reducing conditions. Our group recently demonstrated that incorporating phosphine oxide groups can render terthiophene (**IV**) exceptionally robust toward electron uptake at extreme potentials, making it a promising RFB electrolyte candidate.^[^ ^32^ ^]^ Although effective, such systems typically employ π‐extended substituents that delocalize spin density beyond the thiophene core, thereby complicating direct study of its intrinsic electronic structure. Further, π‐extension is often associated with a higher molecular weight and greater synthetic complexity. These characteristics may pose challenges for large‐scale energy storage where low molecular weight and synthetic simplicity are critical.^[^ ^10^ ^]^ Identifying “minimalist” stabilizing groups is hence of interest, both to more accurately model n‐type charge transport in oligothiophenes and to unlock more practical redox‐active materials for electron storage.

Ester‐functionalized thiophenes offer an enticing solution to this challenge owing to their structural simplicity and straightforward synthesis from adipic acid, an inexpensive commodity chemical.^[^ ^33^, ^34^ ^]^ Further, electrochemical studies have shown that ester‐functionalized thiophenes exhibit reversible cathodic features, enabling their transient reduction in electrochromic devices.^[^ ^35^, ^36^ ^]^ Inspired by this foundation, we sought to determine whether reduced ester‐functionalized thiophenes could be isolated and utilized for long‐term electron storage. Herein, we demonstrate that appended ester moieties render the thiophene platform remarkably robust for galvanostatic charge–discharge cycling at extreme potentials. Additionally, we show that ester substituents allow for the structural and spectroscopic characterization of an anionic thiophene‐centered radical with minimal π‐extension (Scheme 1c). Besides affording fundamental insights into the electronic structure of stable thiophene radicals, our results establish that feedstock‐derived ester‐functionalized thiophenes can be applied as high‐performance electrolytes for energy storage.

Ester‐functionalized thiophenes can be readily synthesized using reported procedures, either via acid‐catalyzed esterification of thiophene‐2,5‐dicarboxylic acid ^[^ ^37^ ^]^ or by reacting thiophene‐2,5‐dicarbonyl dichloride with alcohols.^[^ ^36^ ^]^ Both precursors are readily accessible from adipic acid, rendering the overall route cost‐effective and amenable to scale‐up (Figure S8).^[^ ^33^, ^34^ ^]^ To assess the suitability of ester functionalized thiophenes for electron storage, the cyclic voltammogram (CV) of 2,5‐(CO_2_Me)_2_‐TH (**1‐Me**) was recorded in MeCN solution at room temperature. Shown in Scheme 2a, the CV of **1‐Me** exhibits a fully reversible cathodic wave with a half‐wave potential (*E*_1/2_) of −1.96 V versus Fc/Fc^+^ in MeCN (Fc = ferrocene). This feature is assigned to a one‐electron reduction and retains full reversibility even upon extended cycling (Scheme 2b), consistent with the reported electrochemical behavior and optical switchability of ester‐functionalized thiophenes in electrochromic devices.^[^ ^36^ ^]^ The CV of **1‐Me** stands in stark contrast to thiophene and thiophene‐2,5‐dicarboxylic acid which exhibit only irreversible cathodic features (Figures S36–S38). Collecting the CV of **1‐Me** in the presence of [Li][PF_6_] supporting electrolyte instead of [(*n*‐Bu)_4 _N][PF_6_] led to the observation of a minor anodic *E*_1/2_ shift to −1.89 V, signaling an interaction between the ester‐functionalized thiophene and the Lewis acidic alkali metal ion (Figure S35). Based on these data, it was inferred that the ester functionalities are responsible for imparting the thiophene with its observed reductive stability on the CV timescale.

**Scheme 2:**
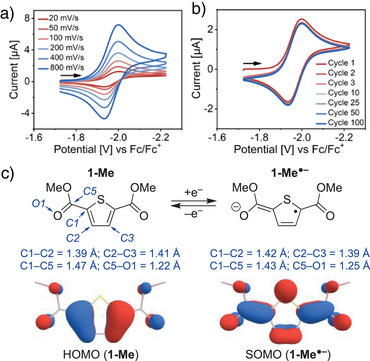
a) The CV of **1‐Me** at various scan rates and b) after extended cycling at 100 mV s^−1^ (r.t., 1.0 mM **1‐Me**, 0.10 M [(*n*‐Bu)_4_N][PF_6_] in MeCN, glassy carbon working electrode). c) DFT‐computed structural changes upon single electron reduction of **1‐Me**, together with frontier molecular orbital illustrations.

The nature of the electronic structure changes taking place upon single‐electron reduction of **1‐Me** was initially probed using density functional theory (DFT) computations. In line with the established electronic structure of thiophenes,^[^ ^38^, ^39^ ^]^ the highest occupied molecular orbital (HOMO) of **1‐Me** exhibits aromatic (benzenoidal) character. Upon reduction, the singly‐occupied molecular orbital (SOMO) of **1‐Me^•–^** shifts toward a “quinoidal” form^[^ ^22^, ^40^ ^]^ with elongation and contraction of intrathiophene C─C contacts (C1─C2 and C2─C3 in Scheme 2c). In the geometry‐optimized structure of **1‐Me^•–^**, the ester groups are coplanar with the thiophene core, with a notable decrease in the C─C bond lengths at their junction (e.g., C1─C5). A slight increase in C─O bond lengths (e.g., C5─O1) can be concurrently observed at the carbonyl groups of **1‐Me^•–^**, highlighting the stabilizing role of the ester functionalities in the radical species.

The redox stability of **1‐Me** motivated us to assess the suitability of ester‐functionalized thiophenes for energy storage, particularly as chargeable anolytes for nonaqueous RFB applications.^[^ ^41^, ^42^, ^43^, ^44^ ^]^ Galvanostatic charge–discharge cycling experiments were carried out in a divided H‐cell, an established method for probing the bulk redox stability of RFB electrolyte candidates (Scheme 3a).^[^ ^45^, ^46^, ^47^, ^48^ ^]^ In a typical experiment, the ester‐functionalized thiophene was dissolved in MeCN (1.0 mM, 0.1 M [(*n*‐Bu)_4 _N][PF_6_]) and reduced by applying a constant current at a reticulated vitreous carbon (RVC) electrode (−0.50 mA; 3C). Reoxidation of the in situ‐generated anion was achieved by reversal of the current (+0.50 mA), thus regenerating the neutral species. Iteration of these charge–discharge steps over time provided quantitative information about the stability (i.e., lifetime) of the redox‐active species under cycling conditions, expressed as the rate of capacity fade over time (% fade h^−1^).^[^ ^49^ ^]^ Given the modular synthesis of ester‐functionalized thiophenes, we aimed to carry out H‐cell experiments for a family of compounds to establish structure–function relationships.

**Scheme 3:**
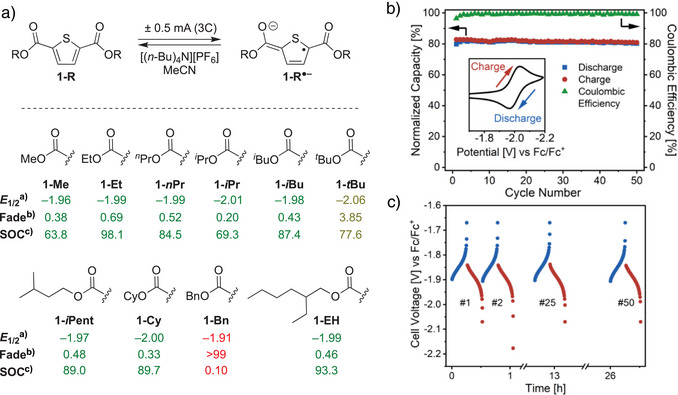
a) Reduction potential (*E*_1/2_) and H‐cell cycling stability of ester‐functionalized thiophenes in MeCN solvent. b) Cell capacity and Coulombic efficiency for H‐cell cycling of **1‐*i*Pr/1‐*i*Pr^•–^** in MeCN:DMF (3:2; *v*:*v*). Inset: CV of **1‐*i*Pr**. c) Cell voltage profile of **1‐*i*Pr/1‐*i*Pr^•–^** cycling. See Supporting Information for electrochemical details. ^a)^V versus Fc/Fc^+^. ^b)^Capacity fade (% fade hr^−1^). ^c)^Maximum state of charge (%).

Shown in Scheme 3a, significant differences in bulk electrochemical stabilities were observed for ester‐functionalized thiophenes under galvanostatic charge–discharge cycling conditions. Linear alkyl esters (**1‐Me**, **1‐Et**, and **1‐*n*Pr**) showed moderate fading (0.38–0.69% h^−1^), while **1‐*i*Pr** exhibited the highest stability (0.20). By contrast, **1‐*t*Bu** showed significantly faster degradation (3.85). Esters containing cyclic or longer chains, (**1‐Cy**, **1‐*i*Pent**, and **1‐EH**) displayed intermediate stabilities (0.33–0.48) while **1‐Bn** underwent complete degradation likely via benzylic fragmentation. Notably, the stability trends presented in Scheme 3a differ from those reported for ester‐functionalized thiophenes in electrochromic devices, where benzyl substituents improved redox‐induced optical switching.^[^ ^36^ ^]^ These differences highlight that developing organic redox systems for energy storage requires unique molecular design criteria. Overall, **1‐*i*Pr** was identified as an optimal electrolyte candidate, combining excellent cycling stability under H‐cell conditions with a low molecular weight desirable for RFB applications.^[^ ^10^ ^]^

Having identified **1‐*i*Pr** as the optimal thiophene derivative, we next examined the influence of solvent on H‐cell cycling performance.^[^ ^50^ ^]^ Among pure solvents, MeCN yielded the best results (0.20% hr^−1^), followed by DMF (0.33) and DME (0.36), with propylene carbonate (PC) resulting in faster degradation (3.7). Interestingly, binary mixtures of MeCN and DMF significantly enhanced stability, likely due to a balance between the fast electron transfer kinetics in MeCN and the broad electrochemical stability window of DMF.^[^ ^48^ ^]^ Specifically, a 1:1 (*v*:*v*) MeCN:DMF mixture yielded 0.11% fade hr^−1^, which improved to 0.086% hr^−1^ in a 3:2 mixture that is among the lowest reported for organic electrolytes in nonaqueous RFBs (Figure S57). Under optimized cycling conditions, **1‐*i*Pr** maintained >98% Coulombic efficiency over 26 h (50 cycles), with 98% of its initial capacity being preserved (Scheme 3b). The voltage profile remained stable throughout cycling (Scheme 3c) and robust performance was observed at both high and low charge‐discharge rates (1–15C; Figures S55, S56). Owing to high solubility in both neutral and charged states (>0.5 M),^[^ ^51^ ^]^ as well as its extremely negative reduction potential, **1‐*i*Pr** delivers a higher theoretical energy density than state‐of‐the‐art organic anolytes (27 Wh L^−1^; Figure S57). These performance metrics, together with its low molecular weight and a feedstock‐derived synthesis, position **1‐*i*Pr** as standout electrolyte candidate for nonaqueous RFBs.

To gain experimental insight into the structural and spectroscopic features of **1‐*i*Pr** upon electron uptake, its chemical reduction was targeted (Scheme 4a). Treating a THF solution of **1‐*i*Pr** with 1 equiv of potassium graphite (KC_8_) in the presence of dibenzo‐18‐crown‐6 (**Bz‐18‐c‐6**) at room temperature resulted in an immediate color change from colorless to deep turquoise. Single crystal X‐ray diffraction identified the product as the ester‐functionalized thiophene anion **[(Bz‐18‐c‐6)K][1‐*i*Pr]**.^[^ ^52^ ^]^ Shown in Scheme 4b, the solid‐state structure of **[(Bz‐18‐c‐6)K][1‐*i*Pr]** features a potassium cation bound equatorially by oxygen atoms of the crown ether. The coordination sphere of potassium is completed by apical oxygens from the carbonyl functionalities of two adjacent **[1‐*i*Pr]^–^** molecules. Consequently, the solid‐state packing of **[(Bz‐18‐c‐6)K][1‐*i*Pr]** is best described as a 1‐D coordination polymer chain (Scheme 4c). As in the case of the DFT‐computed structure for **1‐Me^•–^**, several bond length changes are observable in the experimentally‐determined solid‐state structure of **[(Bz‐18‐c‐6)K][1‐*i*Pr]** relative to a neutral counterpart (**1‐Me**). These include a diagnostic shortening of the C─C bonds linking the ester and thiophene moieties (C1─C5 and C4─C9 in Scheme 4b) alongside the elongation of carbonyl C─O contacts (C5─O1 and C9─O3). These features, in combination with planarization of the ester functionalities, are consistent with a shift toward a quinoidal electronic structure upon reduction. Remarkably, the quinoidal electronic structure in **[(Bz‐18‐c‐6)K][1‐*i*Pr]** is stabilized by the simple ester functionalities without the need for extended π‐delocalization. To our knowledge, **[(Bz‐18‐c‐6)K][1‐*i*Pr]** represents the first crystallographically characterized example of a thiophene radical anion.^[^ ^53^ ^]^

**Scheme 4:**
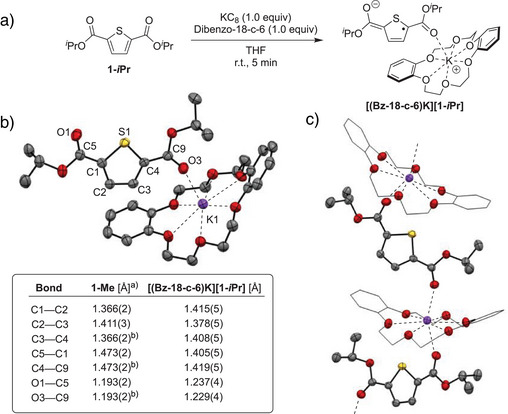
a) Synthesis of **[(Bz‐18‐c‐6)K][1‐*i*Pr]**. b) Solid‐state structure of **[(Bz‐18‐c‐6)K][1‐*i*Pr]** with 50% probability ellipsoids (hydrogen atoms omitted). Key bond length changes in comparison to **1‐Me** are shown. c) Solid‐state packing of **[(Bz‐18‐c‐6)K][1‐*i*Pr]** with 50% probability ellipsoids (hydrogen atoms omitted and carbon atoms in **Bz‐18‐c‐6** are represented without probability ellipsoids). ^a)^ Structural data from reference.^[^ ^54^ ^] b)^Generated by symmetry.

Magnetic and spectroscopic measurements were carried out to further probe the electronic structure of **[(Bz‐18‐c‐6)K][1‐*i*Pr]**. A solution‐state magnetic moment of 1.70 ± 0.06 μ_B_ was measured (Evans method, r.t., THF‐*d_8_*), consistent with an overall *S *= 1/2 ground state. Accordingly, the X‐band electron paramagnetic resonance (EPR) spectrum of the compound exhibits an isotropic signal (*g*_iso_ = 2.0057) in 2‐Me‐THF solution at room temperature, with hyperfine coupling to two equivalent hydrogen atoms {*A*_iso_(^1^H) = 4.36 MHz; Figure 1a}. DFT‐computations for the geometry‐optimized structure of **1‐*i*Pr^•–^** show that the spin density is localized primarily on the thiophene core with a contribution from the appended ester functionalities, consistent with a quinoidal electronic structure description (Figure 1b). The unpaired electron in **[(Bz‐18‐c‐6)K][1‐*i*Pr]** also influences its optical properties. While **1‐*i*Pr** is colorless, **[(Bz‐18‐c‐6)K][1‐*i*Pr]** is dark turquoise and exhibits prominent features in its UV–vis absorption spectrum at λ = 416 nm (*ε* = 2.73 × 10^4^ M^−1^ cm^−1^) and 614 nm (*ε* = 7.17 × 10^3^ M^−1^ cm^−1^) in MeCN solution. Time‐dependent DFT computations {ωB97‐D3, def2‐TZVP, CPCM(MeCN)} were carried out to assign the features as SOMO→LUMO + 1 and SOMO→LUMO transitions, respectively, involving thiophene‐centered orbitals with varying contribution from the ester moieties (Figures S63–S66). Despite the influence of ester substitution, **[(Bz‐18‐c‐6)K][1‐*i*Pr]** mimics the local electronic and structural features of an anionic polaron^[^ ^22^ ^]^ concentrated on a single thiophene ring, and thus serves as a rare isolable model for n‐type doping in polythiophenes.^[^ ^55^ ^]^

**Figure 1:**
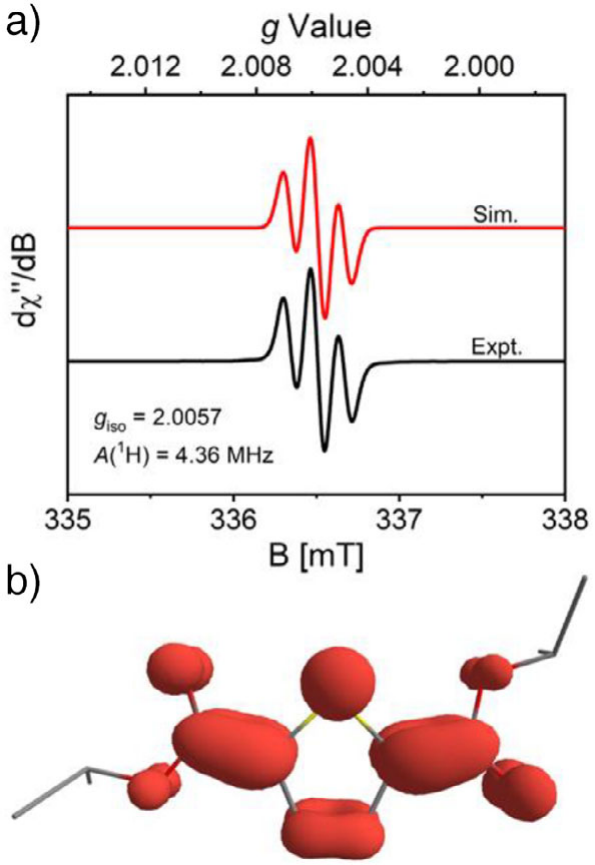
a) X‐band EPR spectrum of **[(Bz‐18‐c‐6)K][1‐*i*Pr]** (2‐Me‐THF, r.t.). b) DFT‐computed spin density plot of **1‐*i*Pr^•−^**.

In conclusion, we have shown that ester‐functionalized thiophenes constitute a structurally simple yet exceptionally stable platform under strongly reducing conditions. Despite their minimalist design, these compounds exhibit robust galvanostatic charge—discharge cycling at high cell voltage, thus circumventing the typical tradeoff between molecular complexity and redox stability at extreme potentials. Combined with high solubility and low molecular weight, we thus disclose that ester‐functionalized thiophenes are cost effective and potentially scalable RFB electrolyte candidates, addressing a central challenge in the field where many organic molecules remain prohibitively complex and expensive.^[^ ^10^ ^]^ Additionally, a crystalline thiophene radical anion **1‐*i*Pr^•−^** was isolated and its electronic structure was probed by single‐crystal X‐ray diffraction as well as spectroscopic and computational methods. Beyond energy storage, **1‐*i*Pr^•−^** represents the smallest isolated molecular analogue of **“**quinoidal” n‐doped polythiophenes. As such, it may serve as a model for understanding the electronic and structural features of conductive oligothiophenes upon reduction.^[^ ^23^, ^56^, ^57^, ^58^ ^]^ Taken together, our results provide a minimalist blueprint that advances both the fundamental understanding and practical deployment of redox systems in energy storage and organic electronics.

## Conflict of Interests

The authors declare no conflict of interest.

## Supporting information



Supporting Information

## Data Availability

The data that support the findings of this study are available in the Supporting Information of this article.
