# Supplementary material for: Feedstock‐Derived Thiophene Radical Anions Exhibiting Redox Stability at Extreme Potentials
Source: Angew Chem Int Ed Engl. 2025 Jul 23;64(37):e202512271. doi: 10.1002/anie.202512271 (PMC12416472; doi:10.1002/anie.202512271)
Supplement: Supplementary file 1 — Supporting Information [file ANIE-64-e202512271-s001.pdf]

Supporting Information

Feedstock-Derived Thiophene Radical Anions Exhibiting Redox Stability at Extreme Potentials

Aurelio C. Gasser, Daniel Käch, and Máté J. Bezdek*

Department of Chemistry and Applied Biosciences

ETH Zürich

Vladimir-Prelog-Weg 1, 8093 Zürich (Switzerland)

*E-mail: mbezdek@ethz.ch

Contents

Materials and Methods 2

Synthetic Protocols 5

NMR Spectra 9

Electrochemical Experiments and Characterization 17

Anolyte Performance Comparison 50

Magnetic Moment Determination 51

Photophysical Data 52

EPR Data 54

X-Ray Data 56

Computational Data 57

References 63

Materials and Methods

General Methods

All air- and moisture-sensitive manipulations were carried out using standard vacuum line Schlenk techniques or in an argon-filled MBraun LabMaster Pro glovebox. Reactions were carried out in oven-dried or flame-dried glassware equipped with a stirbar. Solvents were dried and degassed using an argon-connected MBraun InertGas SPS-7 solvent purification system. The water content was determined using a Mettler-Toledo C30 coulometric Karl-Fischer titrator. Solvents were stored over 3 Å or 4 Å molecular sieves. Chemicals were received from Sigma-Aldrich, Fluorochem, abcr, Acros, STREM, Apollo Scientific Ltd. and were used as received. Thiophene-2,5-dicarboxylic acid was purchased from Apollo Scientific Ltd. and used without further purification. Thiophene-2,5-dicarbonyl dichloride, **1-Me**, **1-Et**, **1-*n*Pr**, **1-*t*Bu**, **1-Bn**,and **1-EH** were synthesized according to literature procedures.[1–6] Basic alumina was purchased from MP Biomedicals and dried at 200 °C under high vacuum for 2 days before use. Solution NMR measurements were carried out on a Bruker Avance 9.4 T spectrometer. NMR spectra were referenced against residual protonated solvent (^{1}H & ^{13}C). Chemical shifts δ are given in ppm and the absolute values of the coupling constants are given in Hertz (Hz). Multiplicities are indicated by s (singlet), d (doublet), t (triplet), vt (virtual triplet), q (quartet), hept (heptet), m (multiplet), br (broad) and combinations thereof.

X-Band EPR experiments were performed using a Magnettech MS-5000 benchtop EPR. Simulations were performed using the EasySpin program.[7] Single crystals suitable for X-ray diffraction were coated with polyisobutenes in a glovebox, transferred onto a nylon loop and mounted on the goniometer of a Rigaku XtaLab Synergy equipped with a molybdenum X-ray tube (λ = 0.71073 Å) and a copper X-ray tube (λ = 1.5406 Å). The structures were solved using direct methods (SHELXT) in the program OLEX2, then completed by Fourier transformation and finally refined by full-matrix least-squares procedures.[8,9] UV-VIS-spectra were recorded on an Agilent Cary5000. FT-IR spectra were collected in the solid state with the ATR technique on a Bruker Alpha inside an MBraun glovebox or a Bruker Tensor II under air. Flash column chromatography was performed on a Biotage Isolera One system with Sfär columns using technical grade solvents. High-resolution mass spectra were provided by the mass spectrometry service facility in the Laboratories of Organic Chemistry at ETH Zürich. The molecular ions $[M]^+$, $[M+H]^+$,$[M+Na^+]$ and $[M+K^+]$ are given in m/z units. NMR spectra are assigned according to the general numbering scheme for the thiophene esters (Figure S1).

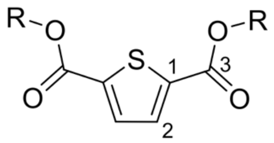

Figure S1: General numbering scheme for the thiophene esters.

Electrochemical Experiments and Characterization

General Methods:

Electrochemical-grade acetonitrile (MeCN) purchased from Sigma-Aldrich was stored over 3 Å molecular sieves. N,N-Dimethylformamide, 99.8%, Extra Dry over Molecular Sieve, AcroSeal® was purchased from Sigma Aldrich and used as received. All solvents were filtered over a short pad of basic alumina prior to use. Tetrabutylammonium hexafluorophosphate {[(*n*-Bu)$_4$N][PF$_6$]} was purchased from Fluorochem, recrystallized twice from hot ethanol (EtOH) and dried under high vacuum for 48 h at 80 °C before use. Potassium hexafluorophosphate (KPF_6) and lithium hexafluorophosphate ($LiPF_6$) were purchased from Sigma-Aldrich in trace metals basis quality and dried under high vacuum for 36 h at 70 °C before use.

Cyclic Voltammetry:

Cyclic Voltammetry (CV) was performed in an argon-filled MBraun UniLab glovebox with a Biologic SP-150e potentiostat using a three-electrode electrochemical cell. If not stated otherwise, a Ag/$AgNO_3$ (0.010 M in [(*n*-Bu)$_4$N][PF$_6$] in MeCN, BASi), glassy carbon disk (0.79 mm^2, eDAQ) and Pt-wire were used as reference, working and counter electrodes, respectively. The working electrode was polished before each experiment on a pad using an alox-slurry (0.05 µm) and rinsed sequentially with millipore water, isopropanol (*i*-PrOH), and acetone. Experiments were conducted using 0.10 M [(*n*-Bu)$_4$N][PF$_6$] supporting electrolyte in MeCN, DMF or propylene carbonate (PC). First, a background scan of a blank sample containing electrolyte and solvent was performed to determine the solvent window and ensure a stable potential and the absence of contaminants. Next, the respective compound (1.0 mM) was dissolved in the blank sample and three scans of the full window starting at the open circuit potential (OCP) were recorded. The first scan is reported. CVs were internally referenced against the Fc/Fc^+ couple. If necessary, data smoothening using the Savitzky-Golay method was applied.

Symmetric H-Cell Charge/Discharge Experiments:

Galvanostatic charge/discharge experiments were performed in a custom-made H-cell in an argon-filled glovebox, adapted from Sanford and coworkers (Figure S2) with a Gamry Interface 1010E potentiostat/galvanostat/ZRA using a three electrode setup.[10] The cell chambers were separated by a P5-frit (15 mm diameter, porosity 5 from Robu®). We employed a cleaning procedure wherein the cell was sonicated 15 min with isopropanol (*i*-PrOH), soaked in water, kept overnight in concentrated nitric acid, soaked in water again, and washed with *i*-PrOH. Prior to use, the cell was oven-dried overnight at 180 °C. Reticulated vitreous carbon (RVC) was purchased from Goodfellow (40 pores/cm) and cut into rods of the dimensions 0.5 cm x 0.5 cm x 4.0 cm. The RVC rods were connected to a Au wire and positioned ca. 1.5 cm deep in solution. The RVC rods were single use and were dried for 16 h at 80 °C under reduced pressure ($6.0*10^{-6}$ bar) prior to use. The reference electrode was a Ag/$AgNO_3$ reference containing 0.010 M $AgNO_3$ in 0.10 M [(*n*-Bu)$_4$N][PF$_6$] in MeCN. Prior to cycling, the compound of interest (1.0 mM) was loaded into the working electrode side together with [(*n*-Bu)$_4$N][PF$_6$] supporting electrolyte (0.10 M) dissolved in 6.0 mL of solvent previously filtered over basic alumina. The counter side of the cell was filled with a solution containing only [(*n*-Bu)$_4$N][PF$_6$] (0.10 M) dissolved in 6.0 mL of solvent previously filtered over basic alumina. Then, a single charge cycle was performed at −0.50 mA and with a stir rate of 500 rpm. The voltage limits were set to approximately $E_{1/2}$ ±0.2 V (vs. internal Ag/$AgNO_3$ reference), where $E_{1/2}$ was previously determined for each compound of interest by CV. Next, the

solution in the counter electrode compartment was discarded and replaced with a fresh solution containing a small excess (typically 0.2 mg) of the compound of interest relative to the working electrode side, 0.10 M [(n-Bu)$_4$N][PF$_6$] dissolved in 6.0 mL of solvent previously filtered over basic alumina.[11] Then, 50 cycles were performed at ± 0.50 mA (3C), 500 rpm with voltage limits as described above.

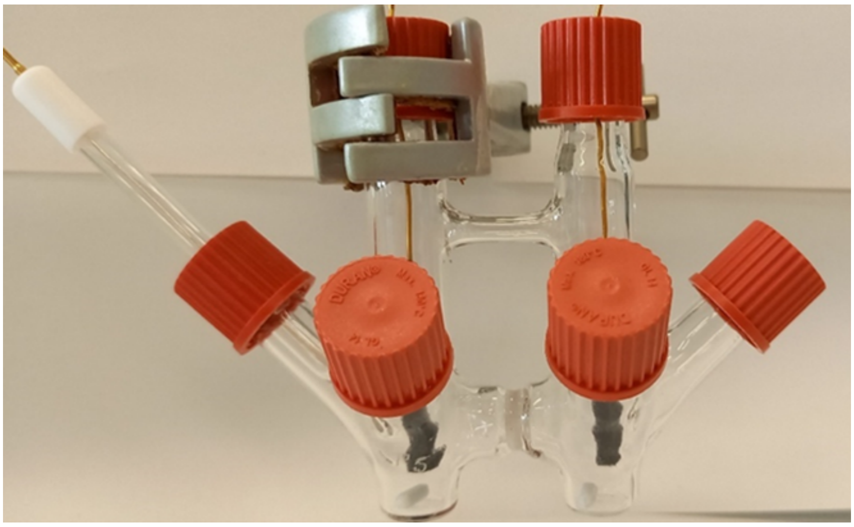

Figure S2: Picture of the assembled H-cell.

Synthetic Protocols

Modified Synthesis of **1-*i*Pr**

1-*i*Pr is reported in the literature[6] but was synthesized using an alternative, simplified procedure adapted from a different report.[3]

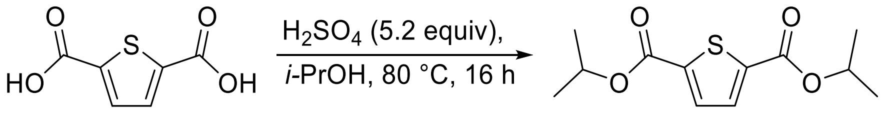

1-*i*Pr

Figure S3: Modified synthesis of **1-*i*Pr**.

A 100-mL oven-dried two-neck round-bottom flask was charged with a magnetic stir bar and thiophene-2,5-dicarboxylic acid (1.0 g, 5.8 mmol). Dry isopropanol (10 mL) was added, resulting in a white suspension. Sulfuric acid (1.6 mL, 30 mmol) was then added and the resulting reaction mixture was heated to 80 °C for 16 h, after which time a clear solution was obtained. The reaction mixture was poured onto excess of EtOAc (25 mL), washed with water (25 mL) once, dried over $MgSO_4$ and concentrated *in vacuo* to obtain **1-*i*Pr** as a colorless oil (0.81 g, 3.2 mmol, 54 %).

Characterization data matched literature report.[6]

Synthesis of **1-*i*Bu**

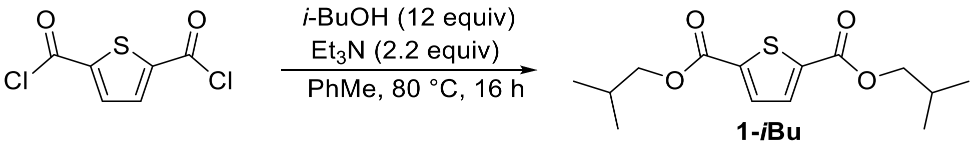

1-*i*Bu

Figure S4: Synthesis of **1-*i*Bu**.

Under air, a 10 ml oven-dried crimp vial equipped with a magnetic stir bar was charged with thiophene-2,5-dicarbonyl dichloride (0.20 g, 0.96 mmol). After sealing the vial with a crimp cap, dry toluene (5.0 mL) and triethylamine (0.29 mL, 2.1 mmol) were added via syringe, resulting in a yellow solution. Isobutanol (1.2 mL, 13 mmol) was then added dropwise and the resulting reaction mixture was heated to 80 °C for 16 h. The reaction mixture was then cooled to room temperature, poured onto excess of DCM (20 mL) and subsequently washed with brine (20 mL) and water (20 mL), one time each. The organic phase was then dried over $MgSO_4$, filtered through Celite and concentrated in vacuo to yield **1-*i*Bu** as an analytically pure brown oil (0.22 g, 0.79 mmol, 83 %).

^{1}H NMR (400 MHz, $CDCl_3$, 298 K): δ 7.74 (s, 2H, ***H2***), 4.10 (d, *J* = 6.6 Hz, 4H, C***H***$_2$), 2.07 (dt, *J* = 13.4, 6.7 Hz, 2H, C***H***), 1.01 (d, *J* = 6.7 Hz, 12H, C***H***$_3$).

^{13}C {^{1}H} NMR (101 MHz, $CDCl_3$, 298 K): δ 161.83 (s, ***C3***), 139.29 (s, ***C1***), 133.02 (s, ***C2***), 71.76 (s, ***C***H$_2$) 28.01 (s, ***C***H), 19.22 (s, ***C***H$_3$).

IR [cm^{-1}]: ṽ = 2959, 2875, 1710, 1532, 1468, 1394,1371,1344, 1240, 1209, 1086, 1032, 975, 944, 836, 790, 748, 532.

HRMS: $[M]^+$ $C_{14}H_{20}NaO_4S$ 307.0975; found 307.0974.

UV-VIS-NIR: λ_{max} (MeCN): 276 nm (ε = 1.2 × 10^4 M^{-1} cm^{-1})

Synthesis of **1-*i*Pent**

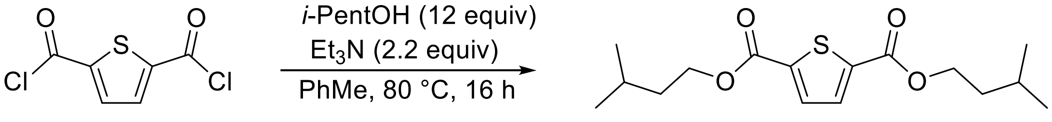

Figure S5: Synthesis of **1-*i*Pent**.

Under air, a 10 ml oven-dried crimp vial equipped with a magnetic stir bar was charged with thiophene-2,5-dicarbonyl dichloride (0.20 g, 0.96 mmol). After sealing the vial with a crimp cap, dry toluene (5.0 mL) and triethylamine (0.29 mL, 2.1 mmol) were added via syringe, resulting in a yellow solution. Isopentanol (1.2 mL, 11 mmol) was then added dropwise and the resulting reaction mixture was heated to 80 °C for 16 h. The reaction mixture was then cooled to room temperature, poured onto excess DCM (20 mL) and subsequently washed with brine (25 mL) and water (25 mL), one time each. The organic phase was then dried over $MgSO_4$ and filtered through Celite. After drying *in vacuo*, the crude product was purified by flash column chromatography using *n*-hexane:DCM (20:80, v:v) as eluent to afford **1-*i*Pent** as a brown oil (0.23 g, 0.72 mmol, 76 %).

^{1}H NMR (400 MHz, $CDCl_3$, 298 K): δ 7.72 (s, 2H, ***H2***), 4.35 (s, 4H, C***H₂***), 1.77 (dp, *J* = 13.3, 6.7 Hz, 2H, C***H***), 1.65 (q, *J* = 6.8 Hz, 4H, C***H₂***), 0.97 (d, *J* = 6.6 Hz, 12H, C***H₃***).

^{13}C {^{1}H} NMR (101 MHz, $CDCl_3$, 298 K): δ 161.86 (s, ***C3***), 139.32 (s, ***C1***), 133.00 (s, ***C2***), 64.54 (s, ***C***H_2), 37.44 (s, ***C***H_2), 25.33 (s, ***C***H), 22.63 (s, ***C***H_3).

IR [cm^{-1}]: ṽ = 2957, 2871, 1710, 1532, 1462, 1386, 1347, 1241, 1170, 1087, 1028, 944, 836, 747, 517.

HRMS: $[M]^+$ $C_{16}H_{24}NaO_4S$ 335.1288; found 335.1292.

UV-VIS-NIR: λ_{max} (MeCN): 276 nm (ε = 2.2 × 10^4 M^{-1} cm^{-1})

Synthesis of **1-Cy**

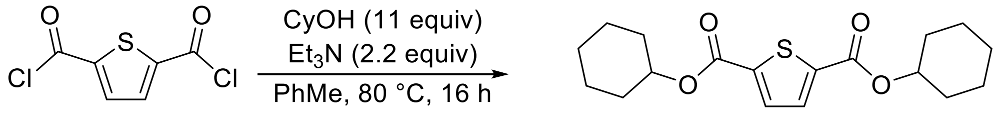

Figure S6: Synthesis of **1-Cy.**

Under air, a 10 ml oven-dried crimp vial equipped with a magnetic stir bar was charged with thiophene-2,5-dicarbonyl dichloride (0.20 g, 0.96 mmol). After sealing the vial with a crimp cap, dry toluene (5.0 mL) and triethylamine (0.29 mL, 2.1 mmol) were added via syringe, resulting in a yellow solution. Cyclohexanol (1.2 mL, 11 mmol) was then added dropwise and the resulting reaction mixture was heated to 80 °C for 16 h. The reaction mixture was then cooled to room temperature, poured onto excess DCM (20 mL) and subsequently washed with brine (25 mL) and

water (25 mL), one time each. The organic phase was then dried over $MgSO_4$, filtered through Celite and dried under high vacuum to afford **1-Cy** as a white solid (86 mg, 0.26 mmol, 27 %).

^{1}H NMR (400 MHz, $CDCl_3$): δ 7.69 (s, 2H, ***H2***), 4.97 (tt, *J* = 8.8, 3.8 Hz, 2H, C***H***), 1.98 – 1.83 (m, 4H, C_6***H***$_{11}$), 1.76 (ddq, *J* = 13.0, 6.6, 3.3 Hz, 4H, C_6***H***$_{11}$), 1.56 (ddq, *J* = 15.8, 9.8, 3.4 Hz, 6H, C_6***H***$_{11}$), 1.46 – 1.22 (m, 6H, C_6***H***$_{11}$).

^{13}C {^{1}H} NMR (101 MHz, $CDCl_3$, 298 K): δ 161.14 (s, ***C3***), 139.65 (s, ***C1***), 132.71 (s, ***C2***), 74.13 (s, ***C***H), 31.58 (s, ***C$_6$***H_{11}), 25.44 (s, ***C$_6$***H_{11}), 23.62 (s, ***C$_6$***H_{11}).

IR [cm^{-1}]: $\tilde{\nu}$ = 2939, 2852, 1705, 1532, 1503, 1452, 1371, 1341, 1317, 1282, 1265, 1239, 1209, 1195, 1091, 1055, 1030, 1008, 933, 901, 890, 848, 830, 821, 789, 747, 680, 583, 559, 517, 502.

HRMS: $[M]^+$ $C_{18}H_{24}NaO_4S$ 359.1288; found 359.1285.

UV-VIS-NIR: λ_{max} (MeCN): 277 nm ($\varepsilon = 1.9 \times 10^4\ M^{-1}\ cm^{-1}$)

Synthesis of **[(Bz-18-c-6)K][1-*i*Pr]**

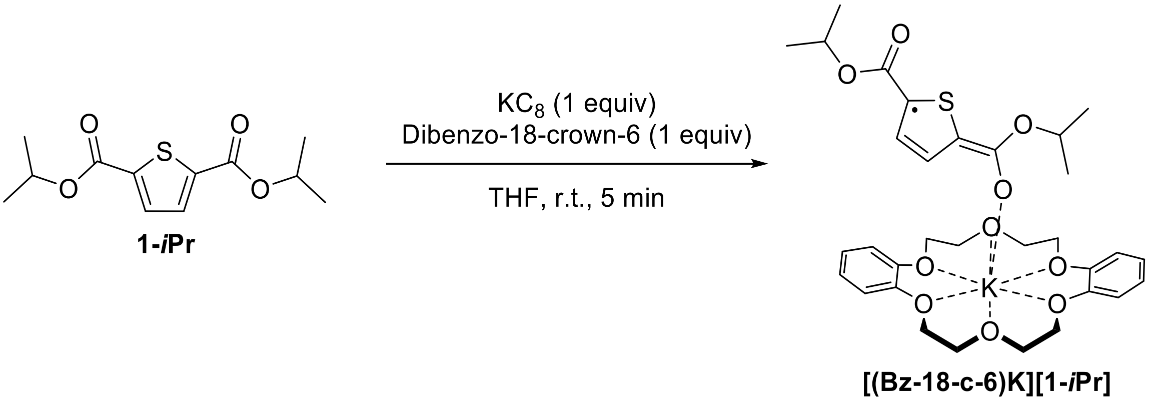

Figure S7: Synthesis of **[(Bz-18-c-6)K][1-*i*Pr]**.

In an argon-filled glovebox, a 5 mL scintillation vial was charged with a magnetic stir bar and **1-*i*Pr** (50 mg, 0.20 mmol). Then, THF (2.0 mL, previously filtered over a short pad of basic alumina) was added to the vial. To the resulting colorless solution, KC_8 (26 mg, 0.20 mmol) and dibenzo-18-crown-6 (70 mg, 0.20 mmol) were added, immediately resulting in a dark turquoise suspension. After 5 minutes of stirring at room temperature, the reaction mixture was filtered over a short pad of Celite and the filtrate was subsequently layered with 10 ml of *n*-hexane. After 16 hours of standing at room temperature, the supernatant was decanted and the solids were dried *in vacuo* to yield **[(Bz-18-c-6)K][1-*i*Pr]** as a dark green crystalline solid (110 mg, 0.17 mmol, 85%). Single crystals suitable for X-ray diffraction were obtained by vapor diffusion of *n*-hexane into a saturated THF solution of **[(Bz-18-c-6)K][1-*i*Pr]** at room temperature.

Solubility: In an argon-filled glovebox, a 15 mL scintillation vial was charged with **[(Bz-18-c-6)K][1-*i*Pr]** (0.45 g, 0.69 mmol) dissolved in 1.5 mL of MeCN/DMF (3:2, *v*:*v*) containing 0.10 M $[(n\text{-}Bu)_4N][PF_6]$ supporting electrolyte. After shaking the vial, all species were solubilized, yielding an estimated lower bound for the solubility of **[(Bz-18-c-6)K][1-*i*Pr]** as ≈ 0.5 M.

EPR: g_{iso} (2-Me THF, 298 K) = 2.0057.

IR [cm^{-1}]: $\tilde{\nu}$ = 3064, 2970, 2928, 2873, 2123, 1867, 1683, 1636, 1595, 1560, 1503, 1467, 1439, 1380, 1360, 1328, 1269, 1247, 1214, 1173, 1122, 1038, 1010, 955, 942, 904, 851 ,826 ,780 ,737, 726, 705, 633, 600, 527, 491, 469, 429

HRMS: [M]+ $C_{32}H_{40}KO_{10}S$ 655.1974; found 655.1975

UV-VIS-NIR: λ_{max} (MeCN): 614 nm (ε = 0.7 × 10^4 M^{-1} cm^{-1}), 416 nm (ε = 2.7 × 10^4 M^{-1} cm^{-1}), 225 nm (ε = 3.0 × 10^4 M^{-1} cm^{-1}).

Note: The thiophene-2,5-dicarboxylic acid and thiophene-2,5-dicarbonyl dichloride starting materials are directly accessible from the commodity chemical adipic acid according to reported procedures (Figure S8). [12,13]

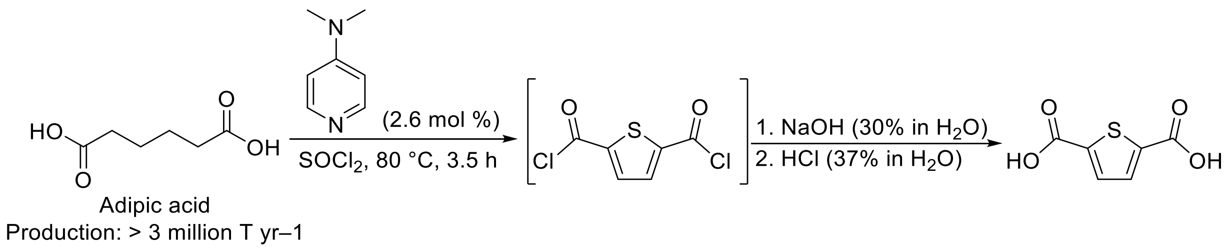

Figure S8: Reported synthesis of thiophene-2,5-dicarbonyl dichloride and thiophene-2,5-dicarboxylic acid from adipic acid.

NMR Spectra

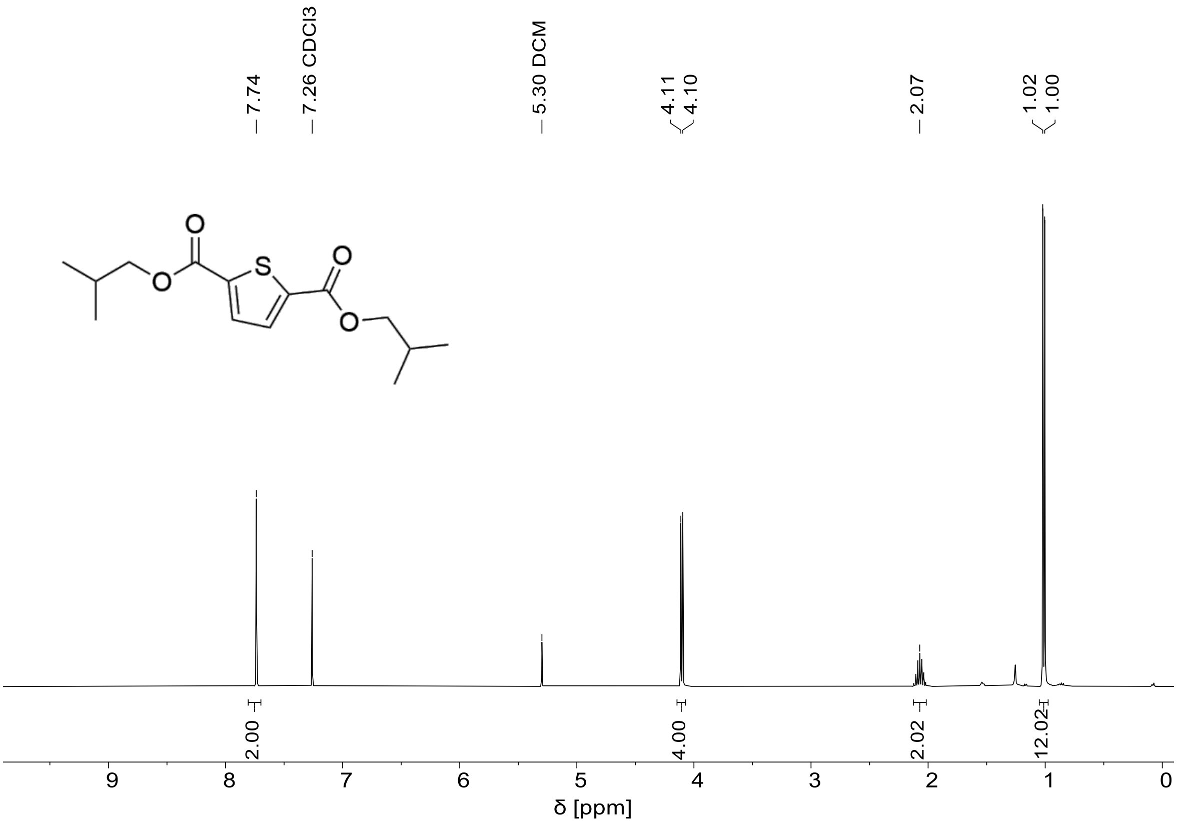

Figure S9: ^{1}H NMR (400 MHz, $CDCl_3$, 298 K) spectrum of **1-*i*Bu**.

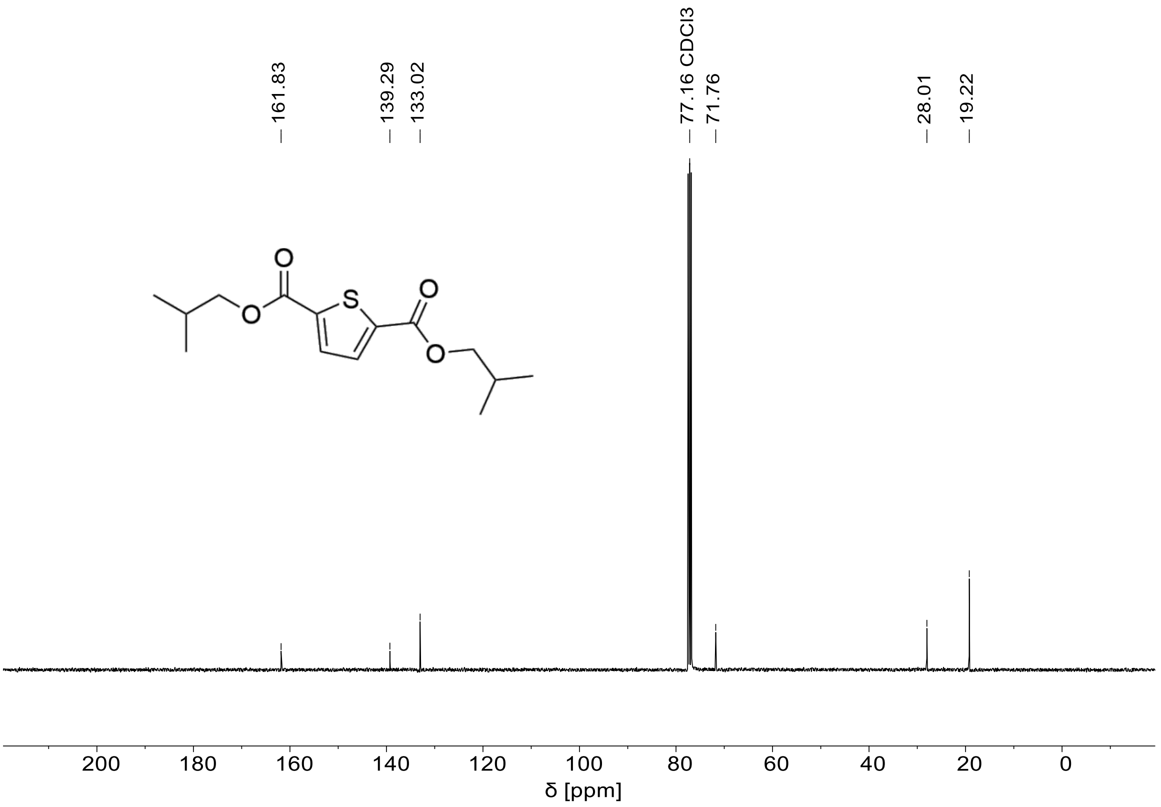

Figure S10: $^{13}C\{^{1}H\}$ NMR (101 MHz, $CDCl_3$, 298 K) spectrum of **1-*i*Bu**.

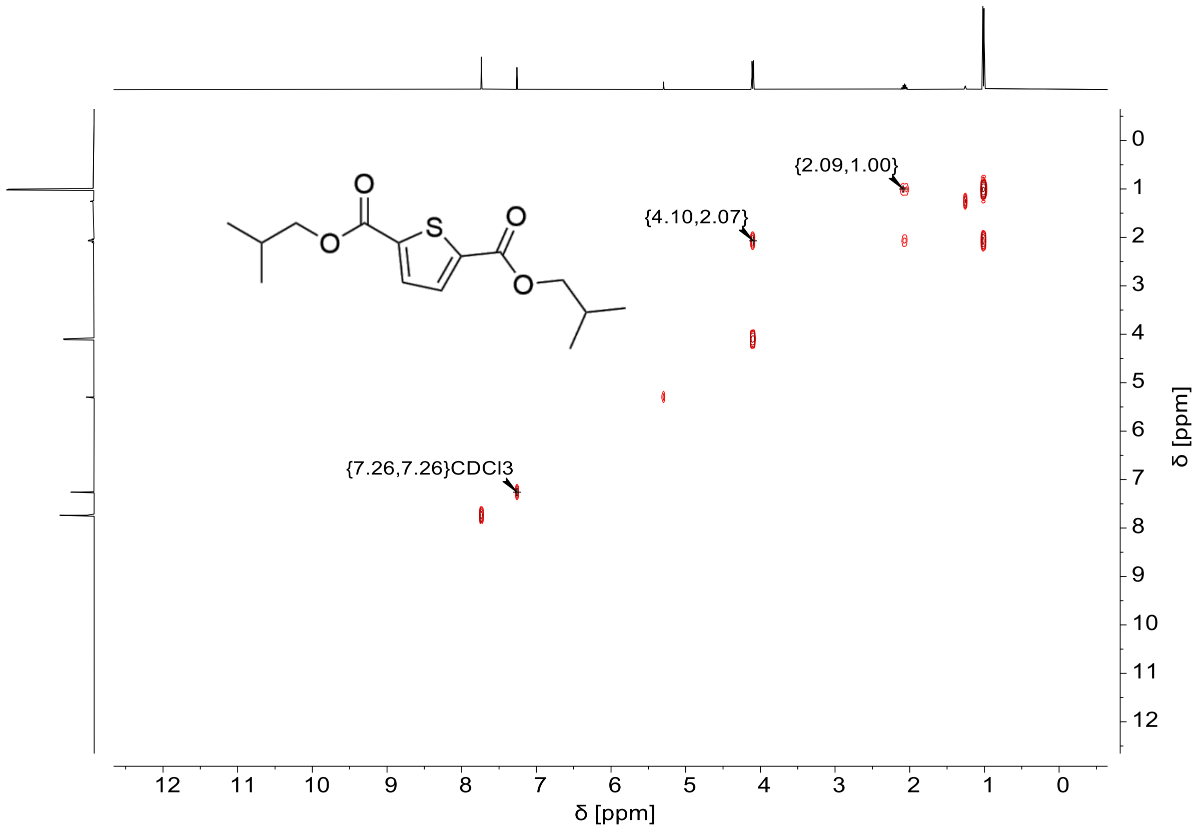

Figure S11: $^{1}H/^{1}H$ COSY NMR (400/400 MHz, $CDCl_3$, 298 K) spectrum of **1-*i*Bu**.

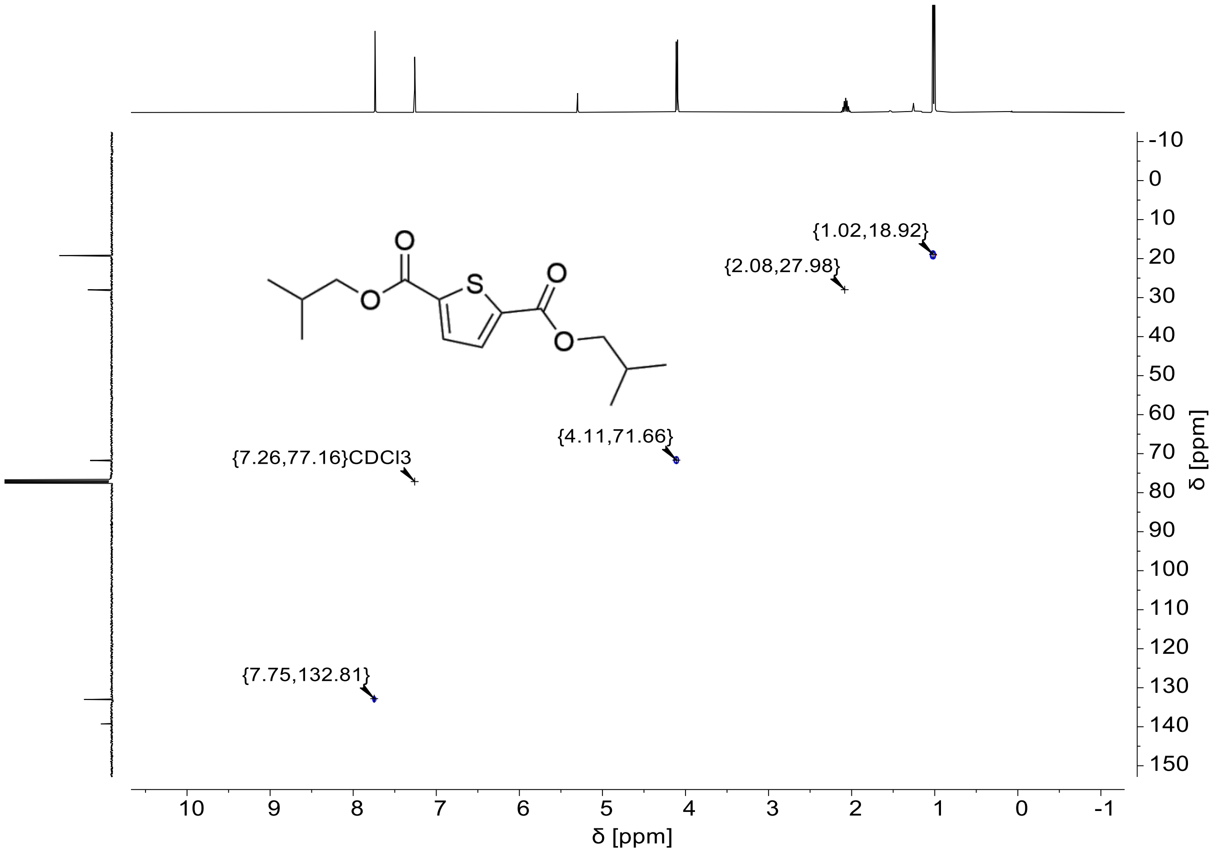

Figure S12: $^{1}H/^{13}C$ HSQC NMR (400/101 MHz, $CDCl_3$, 298 K) spectrum of **1-*i*Bu**.

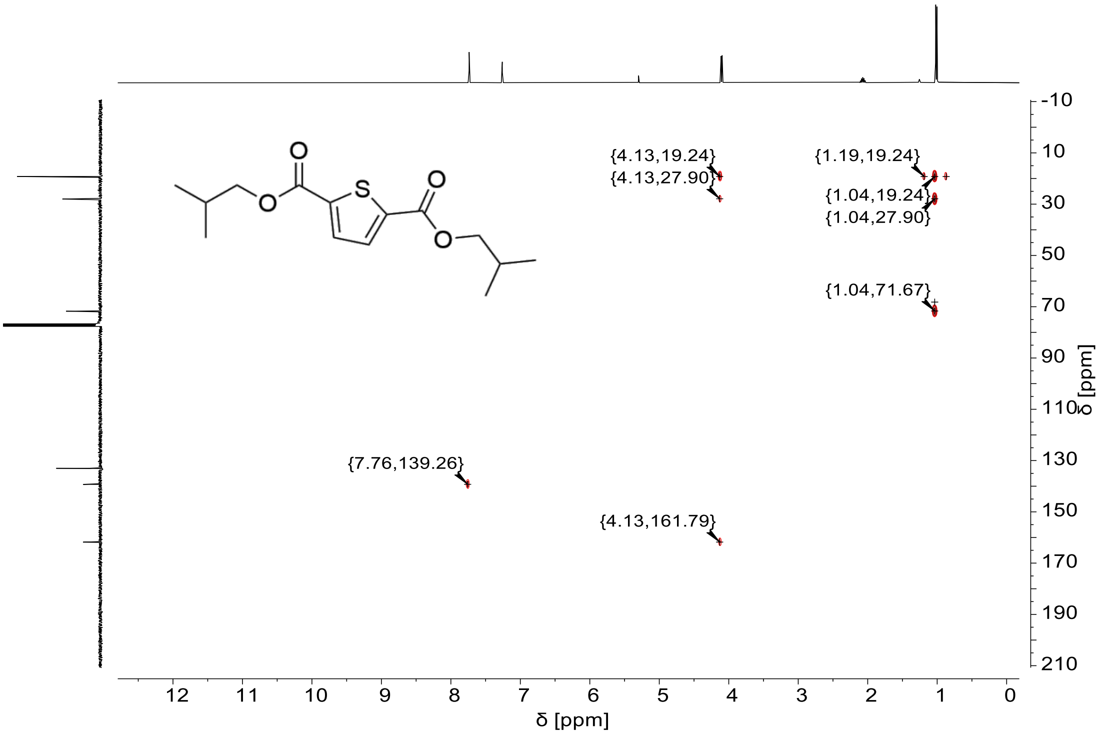

Figure S13: $^{1}H/^{13}C$ HMBC NMR (400/101 MHz, $CDCl_3$, 298 K) spectrum of **1-*i*Bu**.

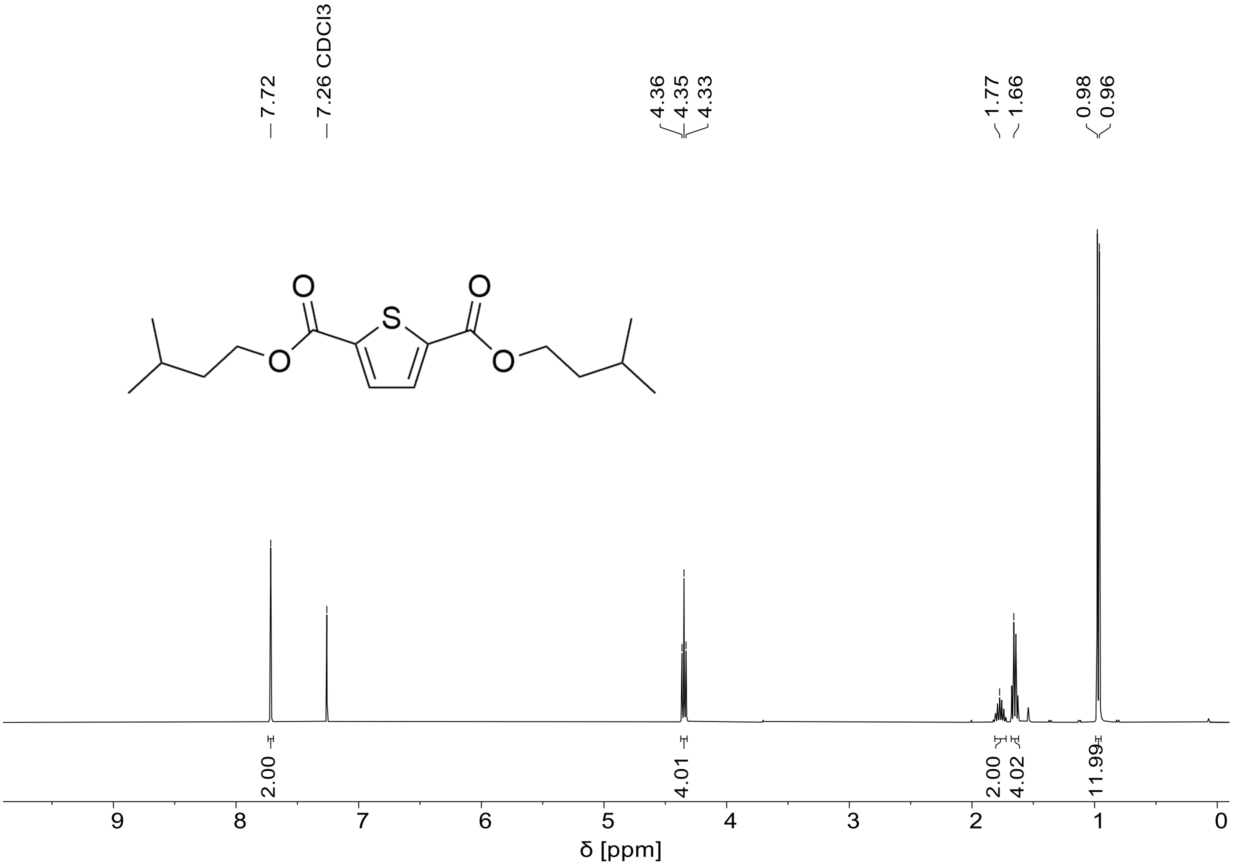

Figure S14: ^{1}H NMR (400 MHz, $CDCl_3$, 298 K) spectrum of **1-*i*Pent**.

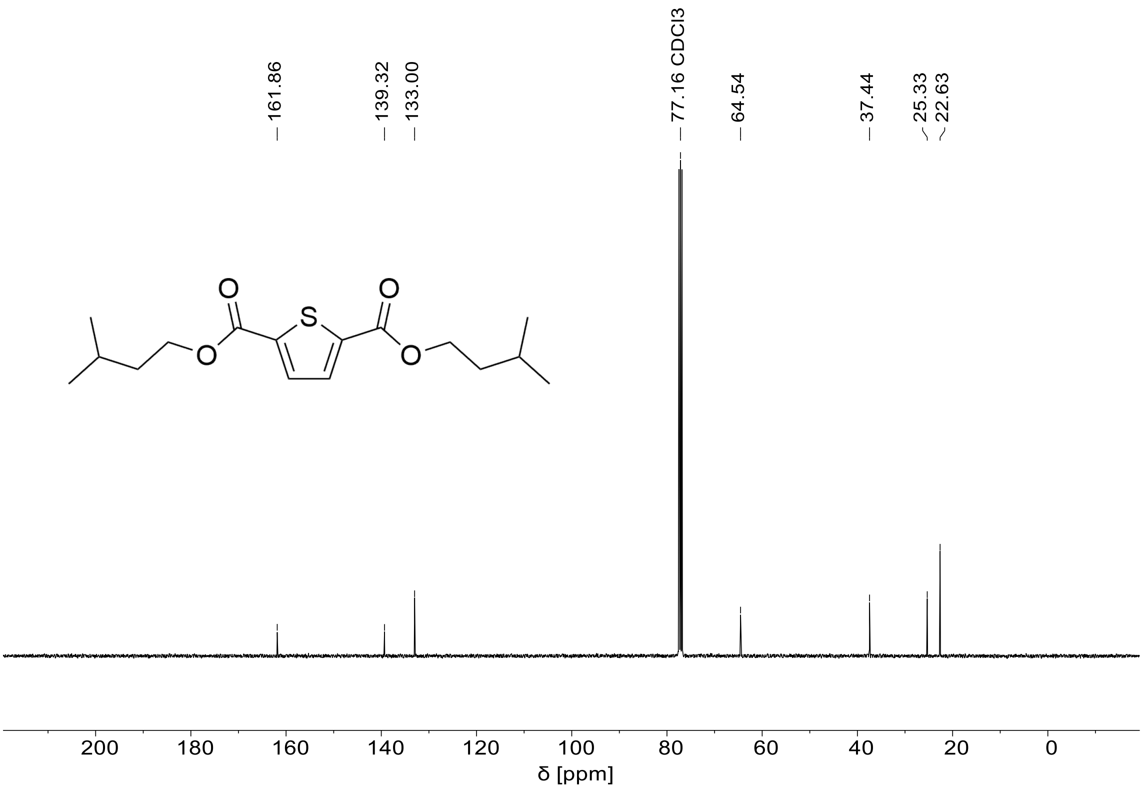

Figure S15: $^{13}C\{^1H\}$ NMR (101 MHz, $CDCl_3$, 298 K) spectrum of **1-*i*Pent**.

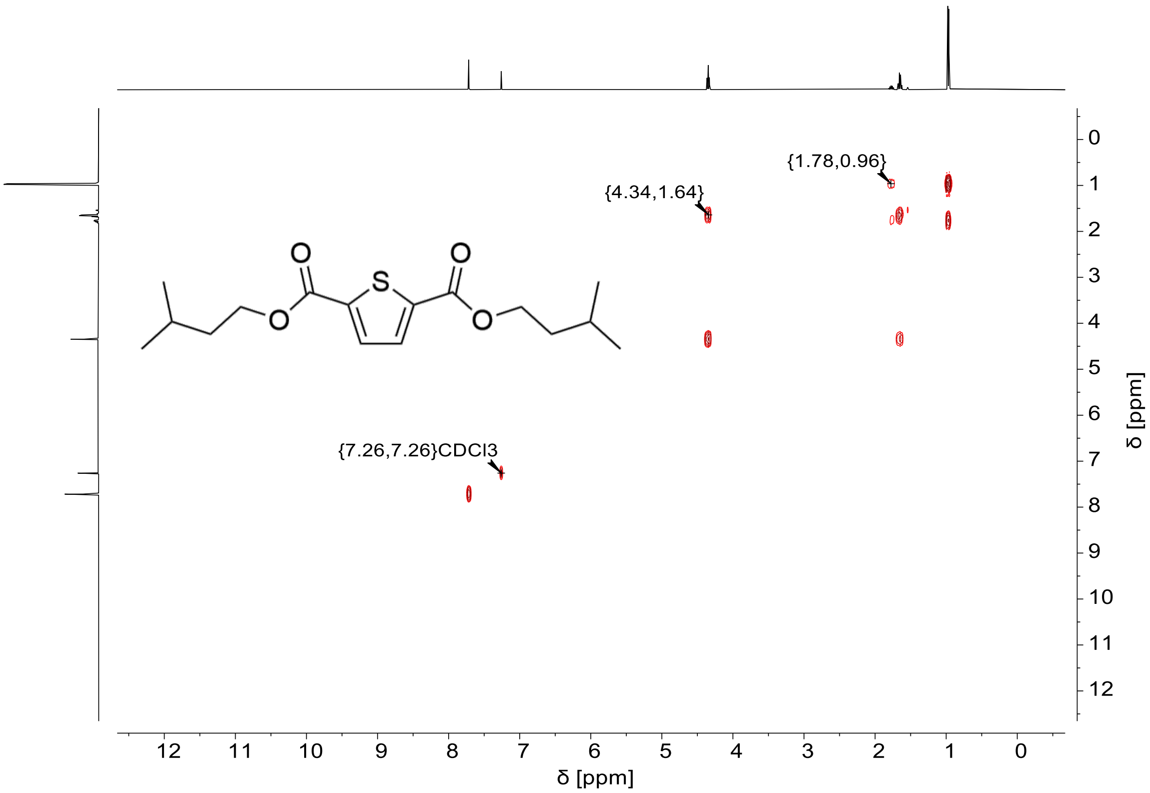

Figure S16: $^1H/^1H$ COSY NMR (400/400 MHz, $CDCl_3$, 298 K) spectrum of **1-*i*Pent.**

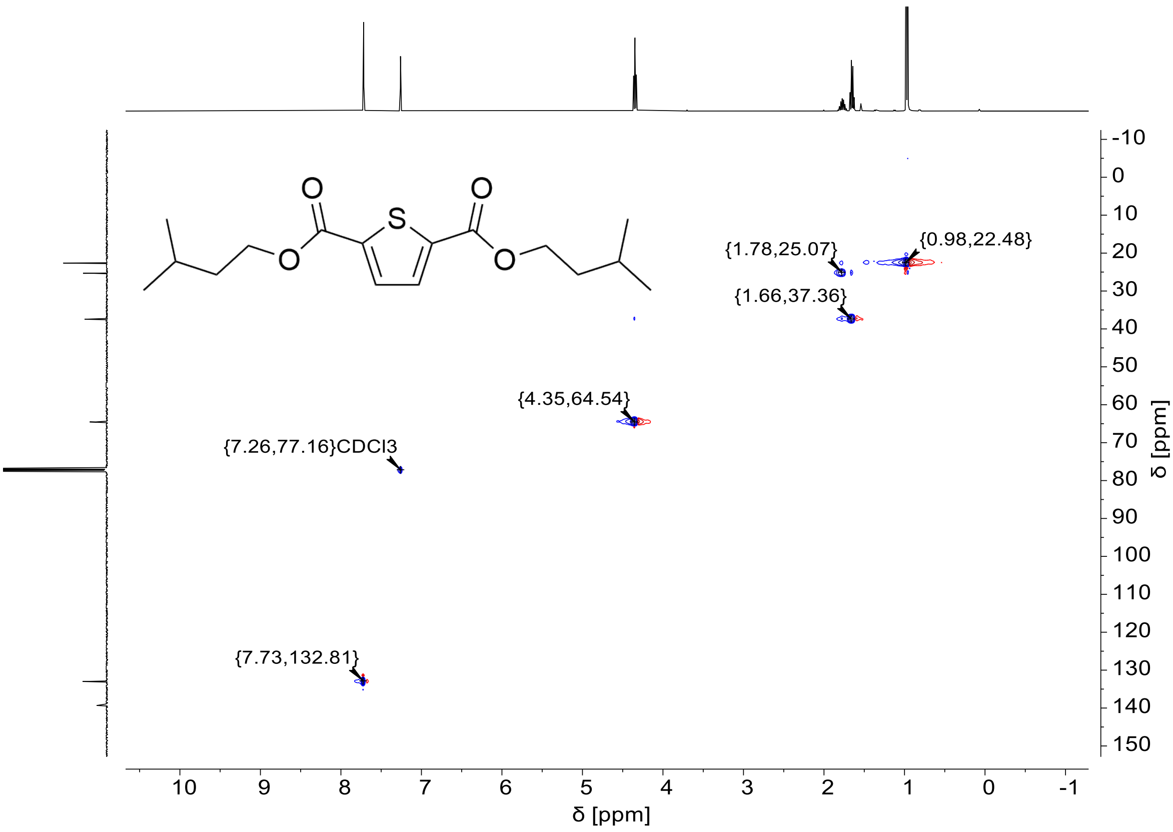

Figure S17: ^{1}H/^{13}C HSQC NMR (400/101 MHz, $CDCl_3$, 298 K) spectrum of **1-*i*Pent**.

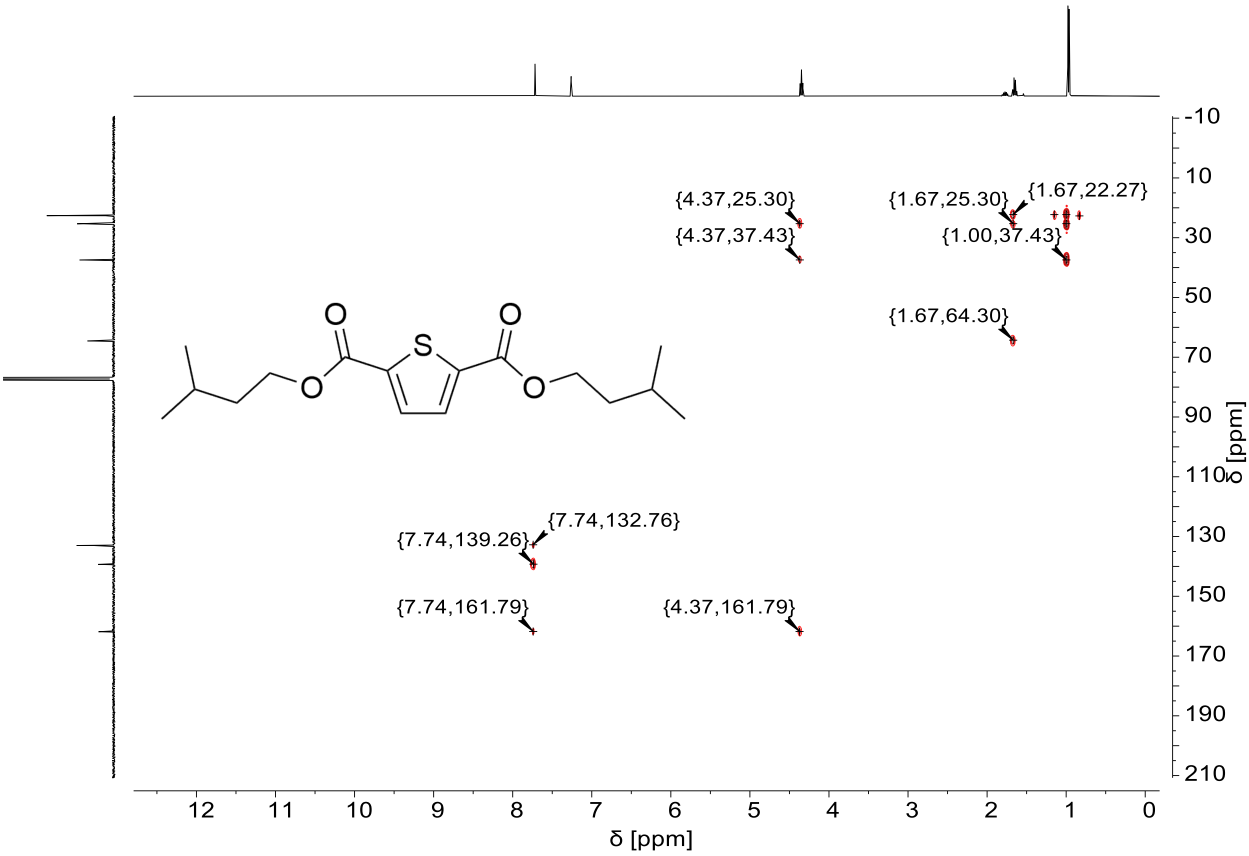

Figure S18: ^{1}H/^{13}C HMBC NMR (400/101 MHz, $CDCl_3$, 298 K) spectrum of **1-*i*Pent**.

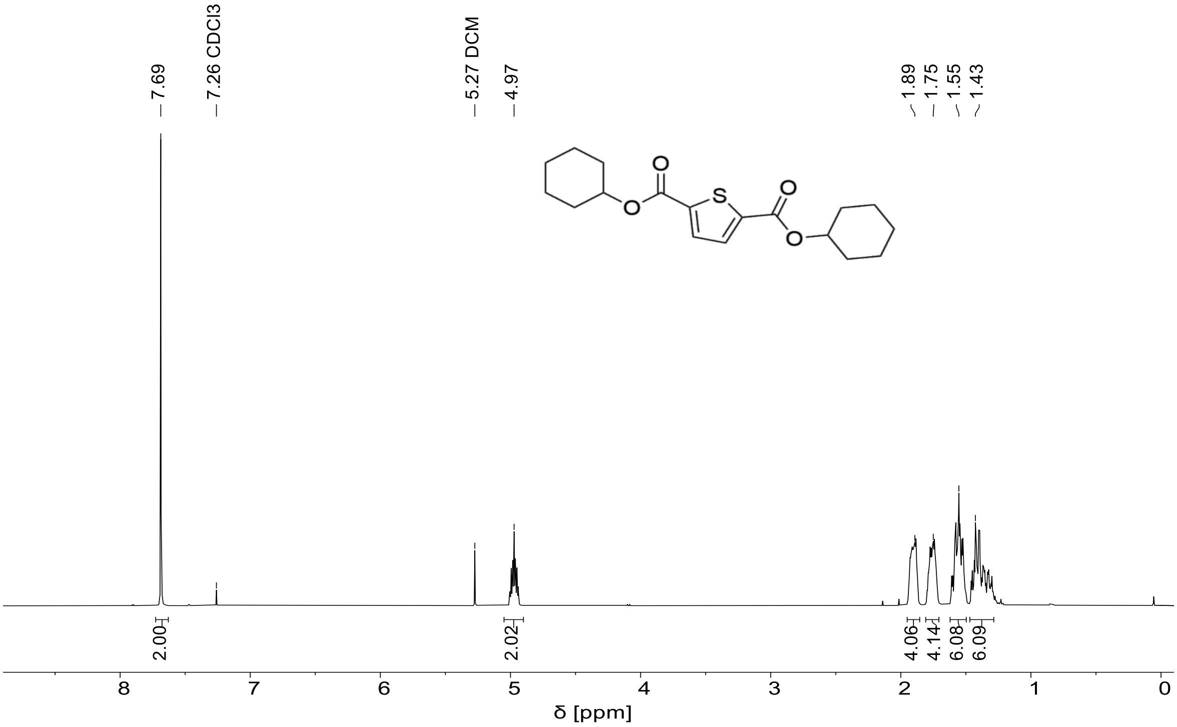

Figure S19: ^{1}H NMR (400 MHz, $CDCl_3$, 298 K) spectrum of **1-Cy**.

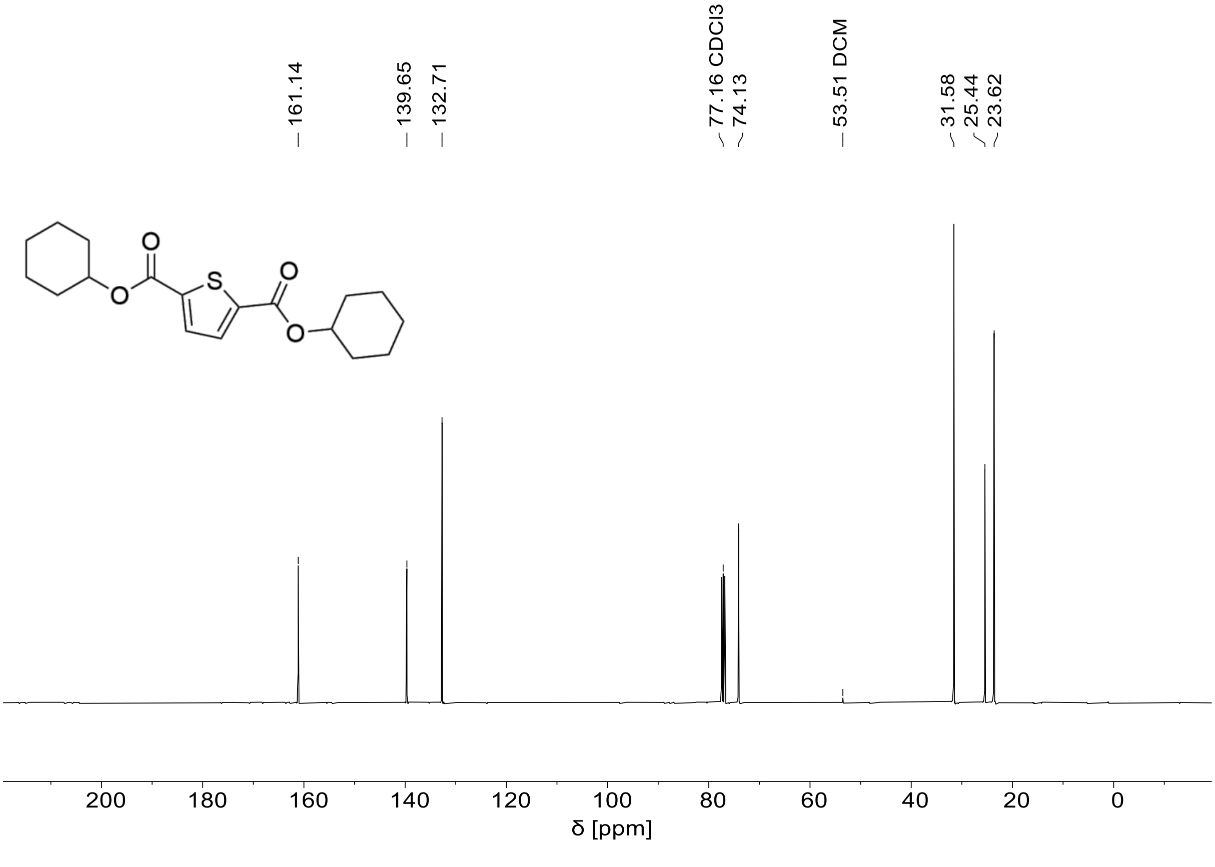

Figure S20: $^{13}C\{^{1}H\}$ NMR (101 MHz, $CDCl_3$, 298 K) spectrum of **1-Cy**.

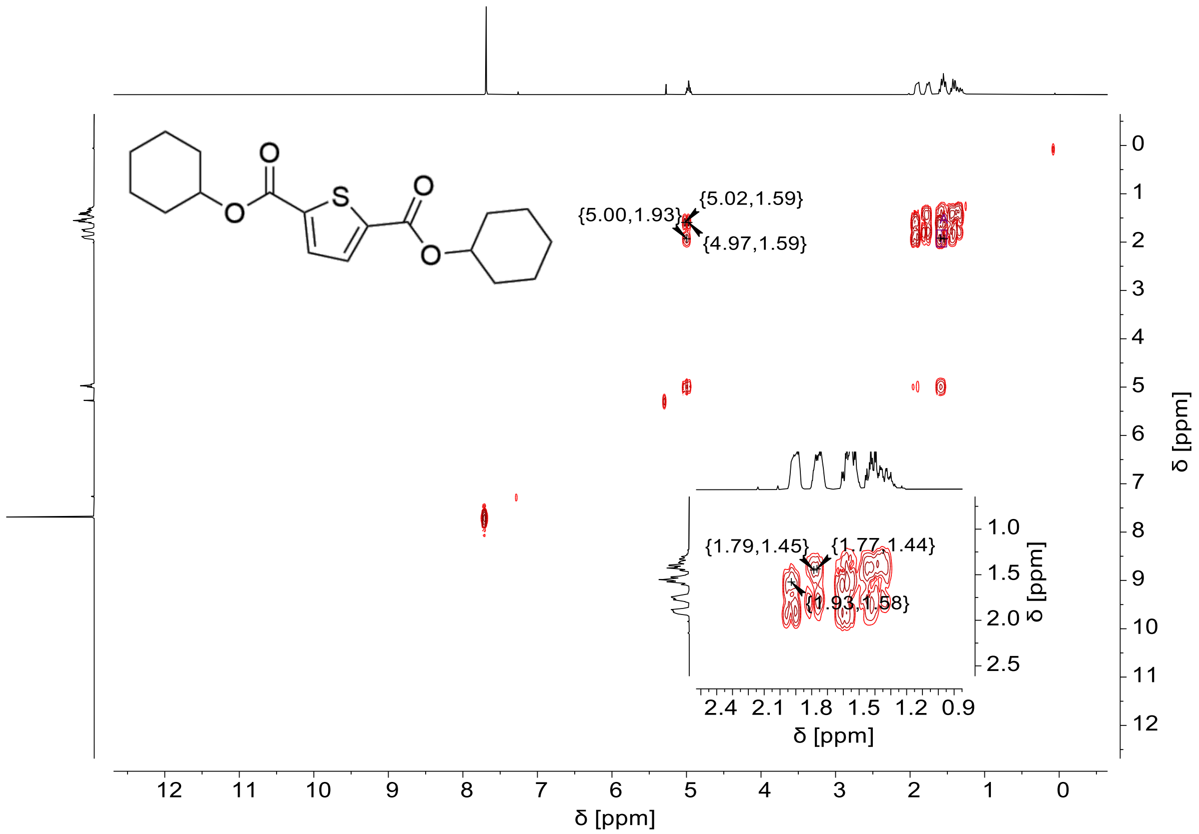

Figure S21: $^{1}H/^{1}H$ COSY NMR (400/400 MHz, $CDCl_3$, 298 K) spectrum of **1-Cy**.

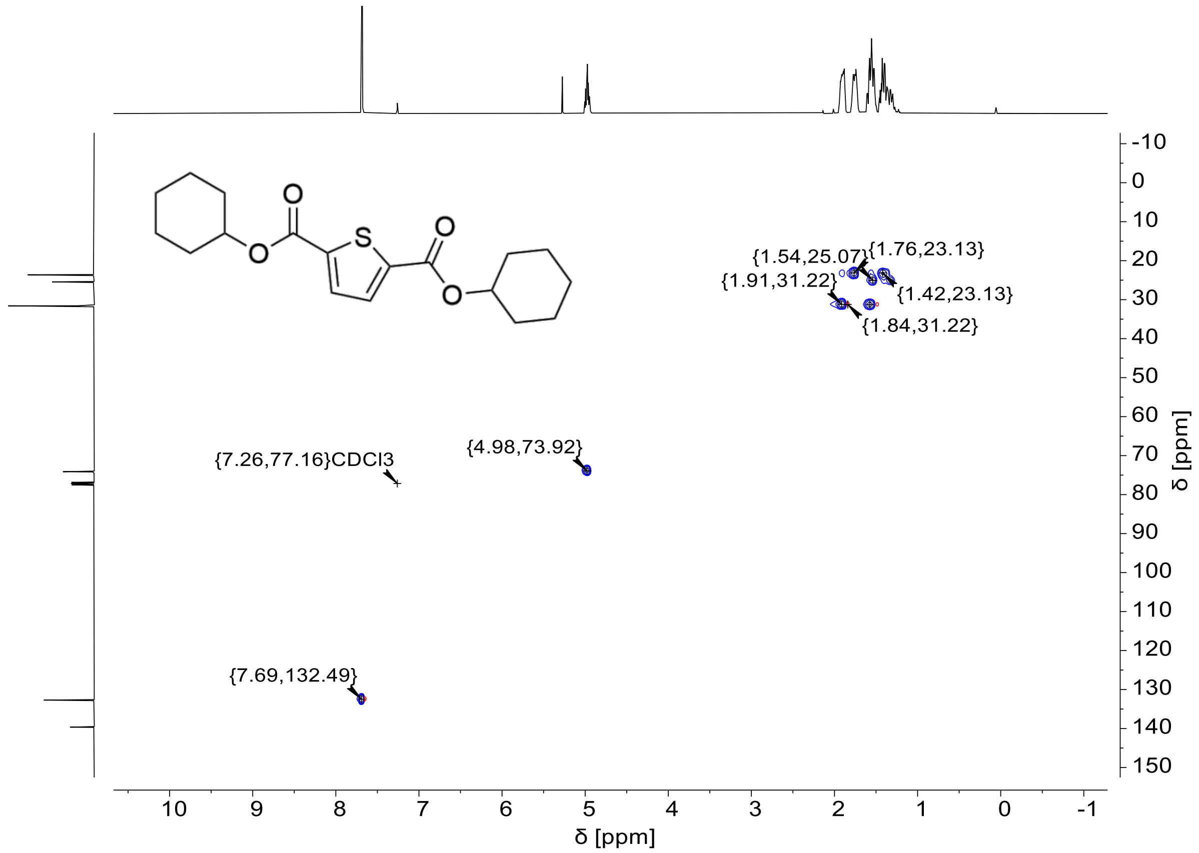

Figure S22: $^{1}H/^{13}C$ HSQC NMR (400/101 MHz, $CDCl_3$, 298 K) spectrum of **1-Cy**.

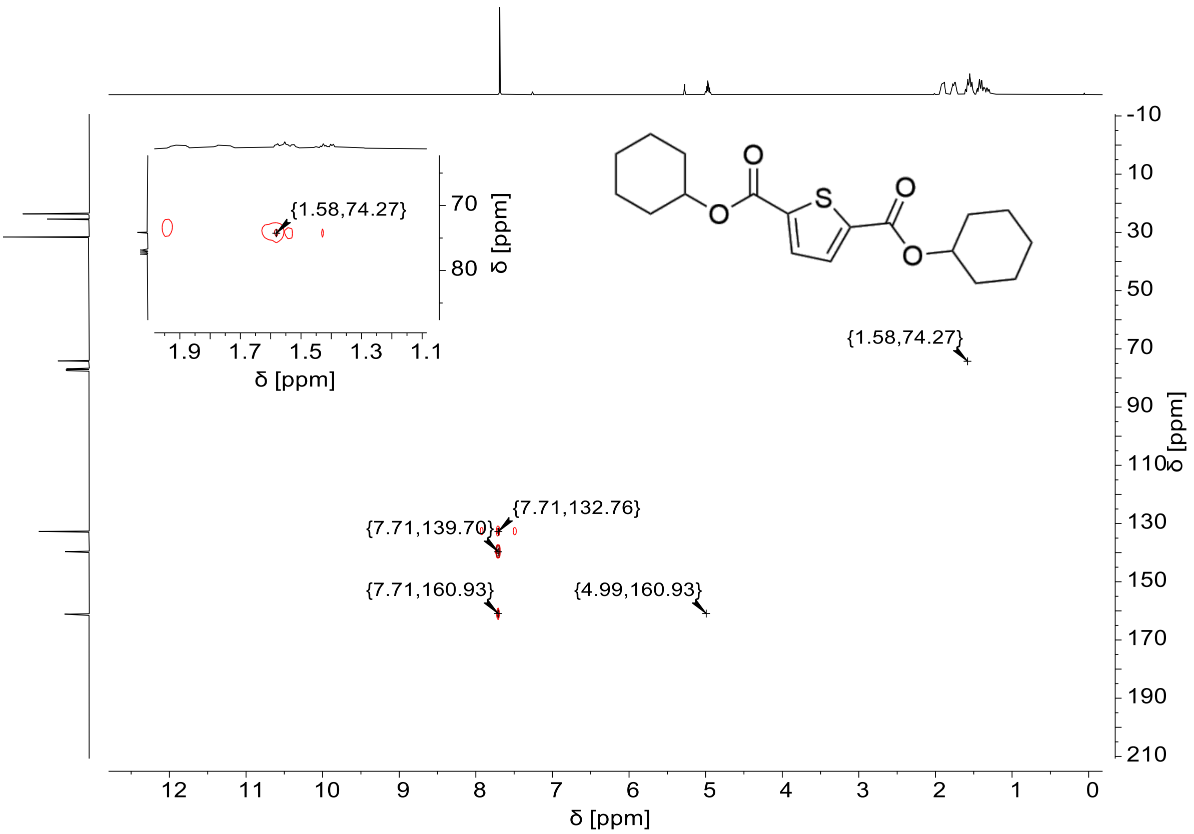

Figure S23: $^{1}H/^{13}C$ HMBC NMR (400/101 MHz, $CDCl_3$, 298 K) spectrum of **1-Cy**.

Electrochemical Experiments and Characterization

Cyclic Voltammetry

Compound **1-Me** in MeCN:

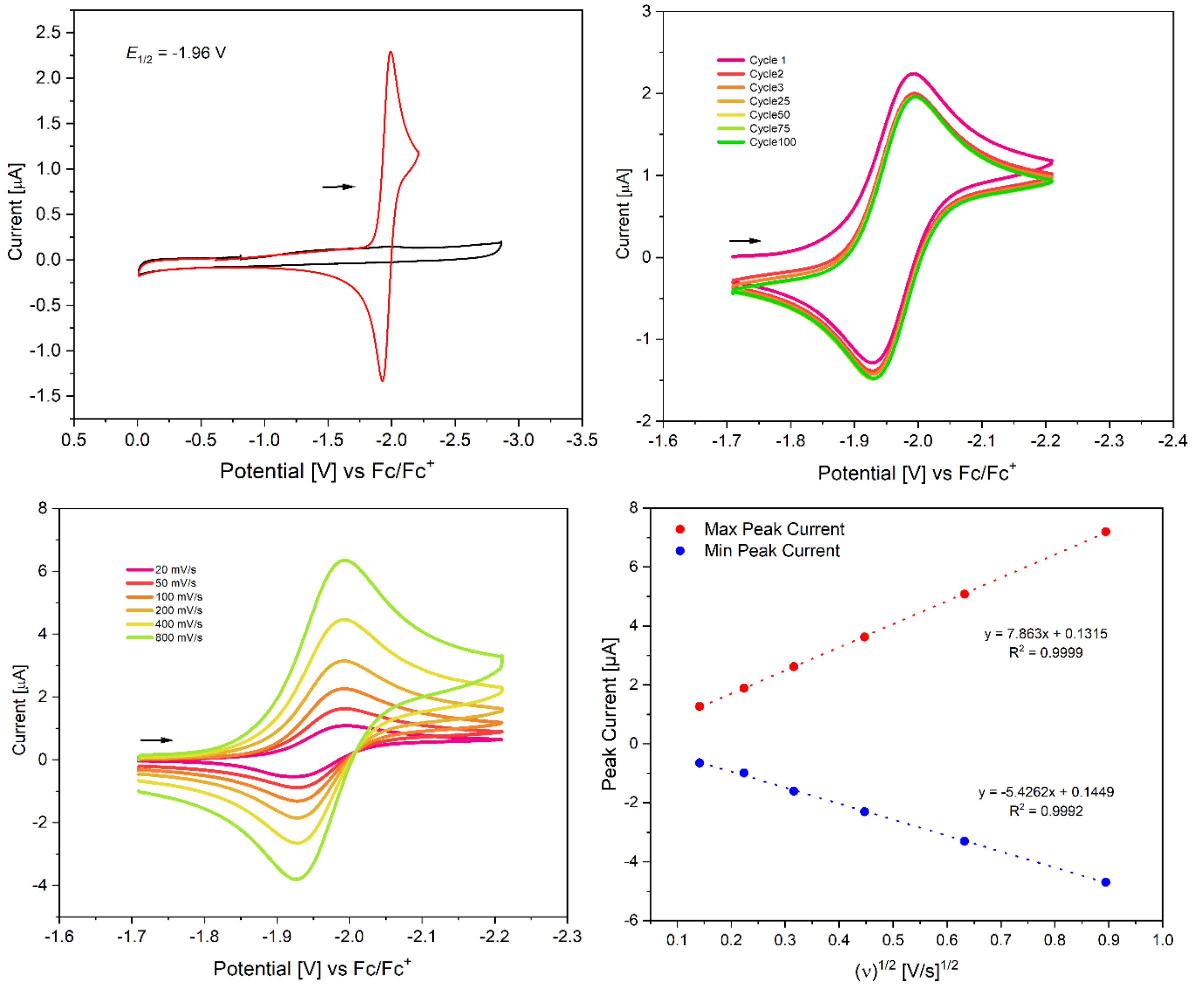

Figure S24: Cyclic voltammogram (top left), cycling (top right) and scan rate study (bottom) of **1-Me** (1.0 mM) with 0.10 M [(*n*-Bu)$_4$N][PF$_6$] as supporting electrolyte in MeCN. Referenced against Fc/Fc$^+$. The full scan (red) was recorded at 100 mV/s and is shown against the background (black) of 0.10 M [(*n*-Bu)$_4$N][PF$_6$] in MeCN.

Compound **1-Et** in MeCN:

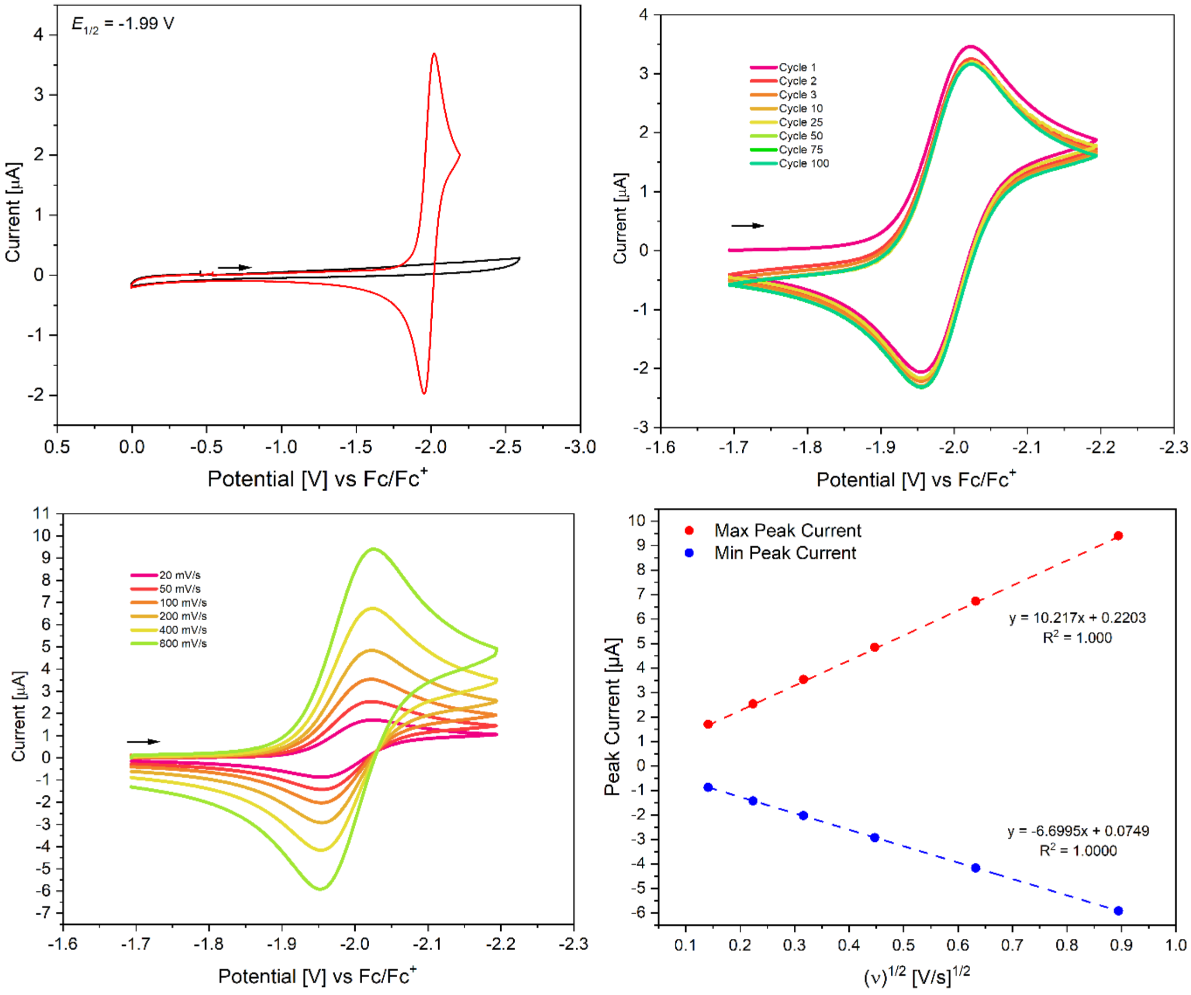

Figure S25: Cyclic voltammogram (top left), cycling (top right) and scan rate study (bottom) of **1-Et** (1.0 mM) with 0.10 M [(*n*-Bu)$_4$N][PF$_6$] as supporting electrolyte in MeCN. Referenced against Fc/Fc$^+$. The full scan (red) was recorded at 100 mV/s and is shown against the background (black) of 0.10 M [(*n*-Bu)$_4$N][PF$_6$] in MeCN.

Compound **1-*n*Pr** in MeCN:

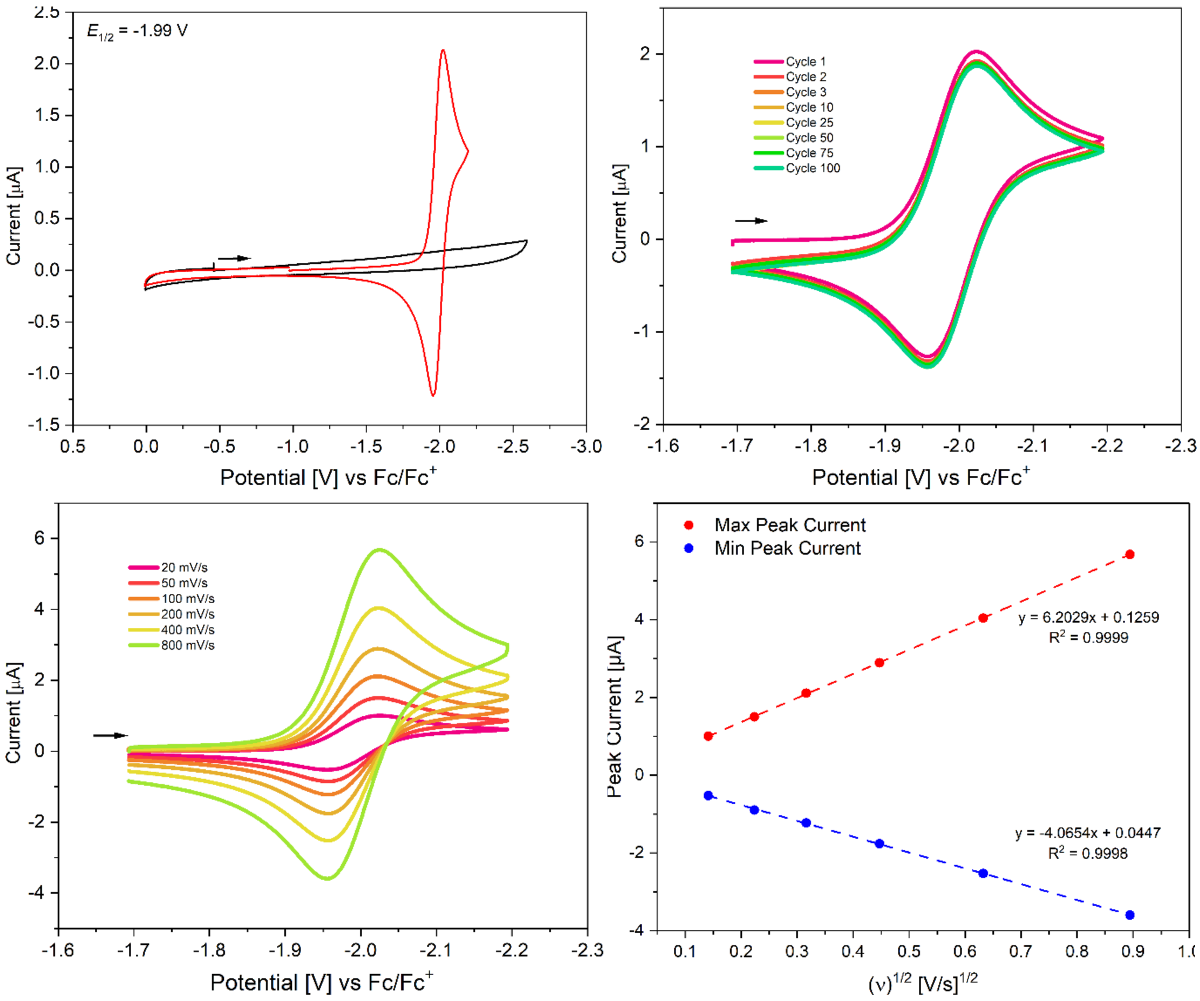

Figure S26: Cyclic voltammogram (top left), cycling (top right) and scan rate study (bottom) of **1-*n*Pr** (1.0 mM) with 0.10 M [(*n*-Bu)$_4$N][PF$_6$] as supporting electrolyte in MeCN. Referenced against Fc/Fc$^+$. The full scan (red) was recorded at 100 mV/s and is shown against the background (black) of 0.10 M [(*n*-Bu)$_4$N][PF$_6$] in MeCN.

Compound **1-*i*Pr** in MeCN:

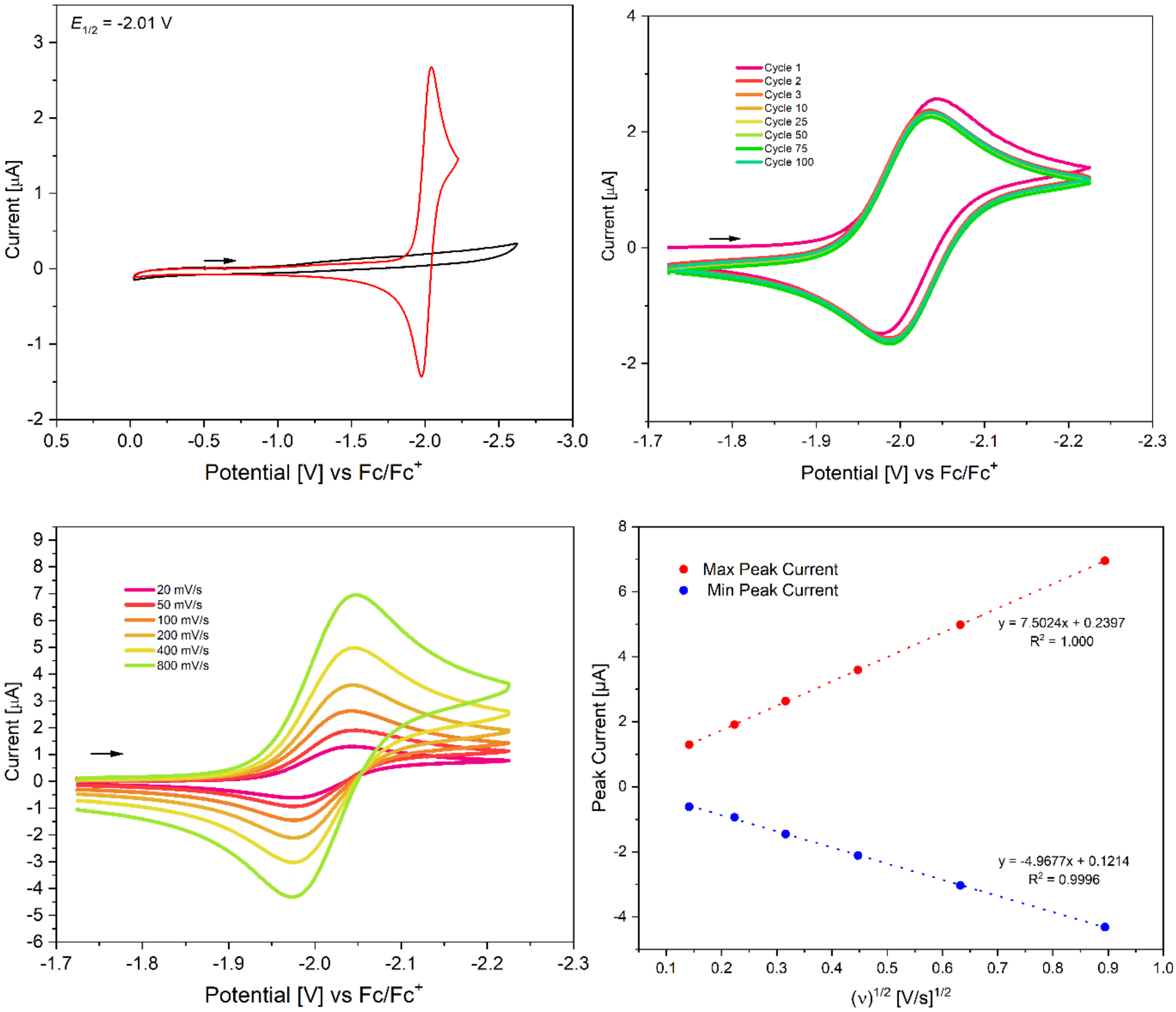

Figure S27: Cyclic voltammogram (top left), cycling (top right) and scan rate study (bottom) of **1-*i*Pr** (1.0 mM) with 0.10 M [(*n*-Bu)$_4$N][PF$_6$] as supporting electrolyte in MeCN. Referenced against Fc/Fc$^+$. The full scan (red) was recorded at 100 mV/s and is shown against the background (black) of 0.10 M [(*n*-Bu)$_4$N][PF$_6$] in MeCN.

Compound **1-*i*Bu** in MeCN:

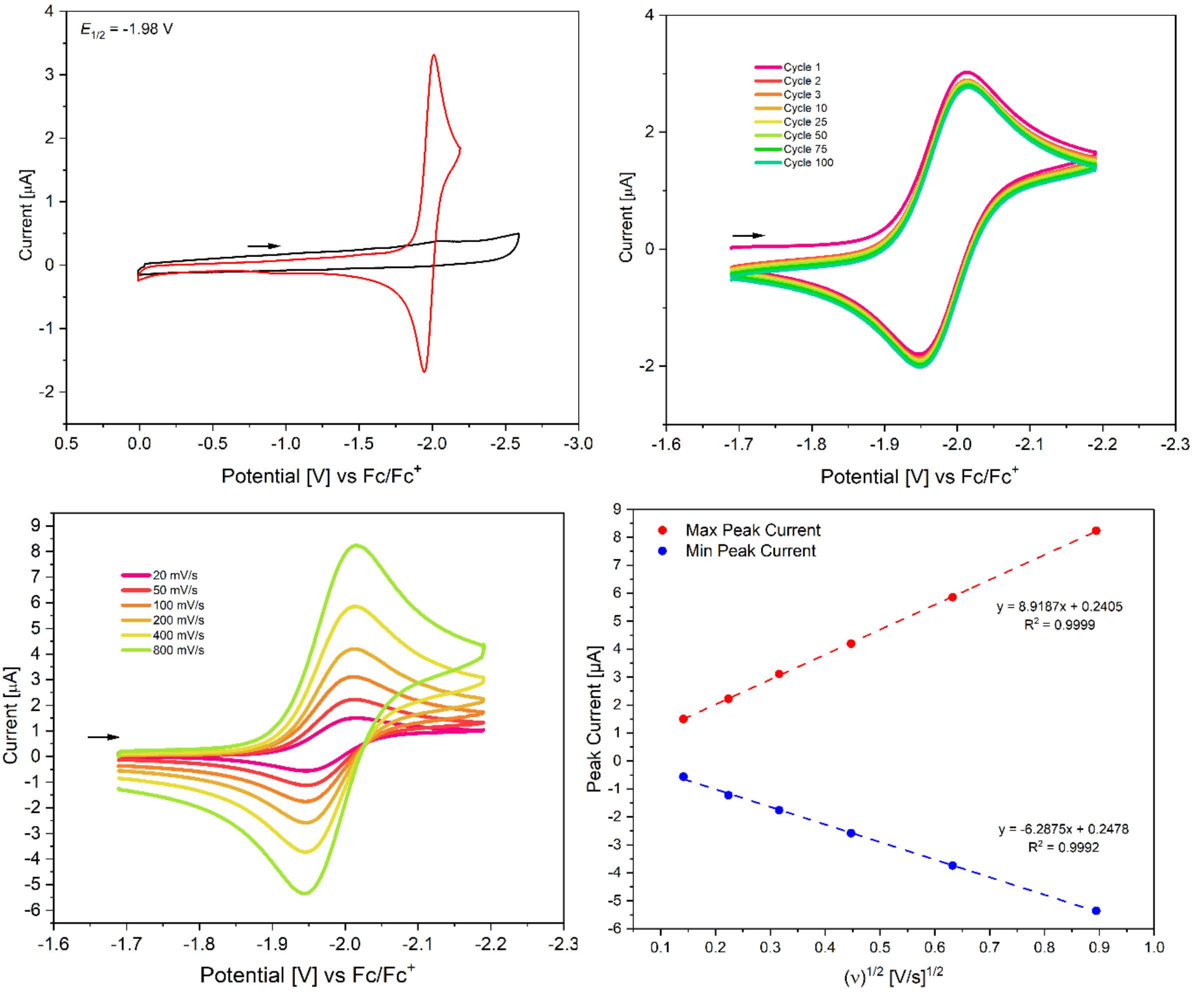

Figure S28: Cyclic voltammogram (top left), cycling (top right) and scan rate study (bottom) of **1-*i*Bu** (1.0 mM) with 0.10 M [(*n*-Bu)$_4$N][PF_6] as supporting electrolyte in MeCN. Referenced against Fc/Fc$^+$. The full scan (red) was recorded at 100 mV/s and is shown against the background (black) of 0.10 M [(*n*-Bu)$_4$N][PF_6] in MeCN.

Compound **1-*t*Bu** in MeCN:

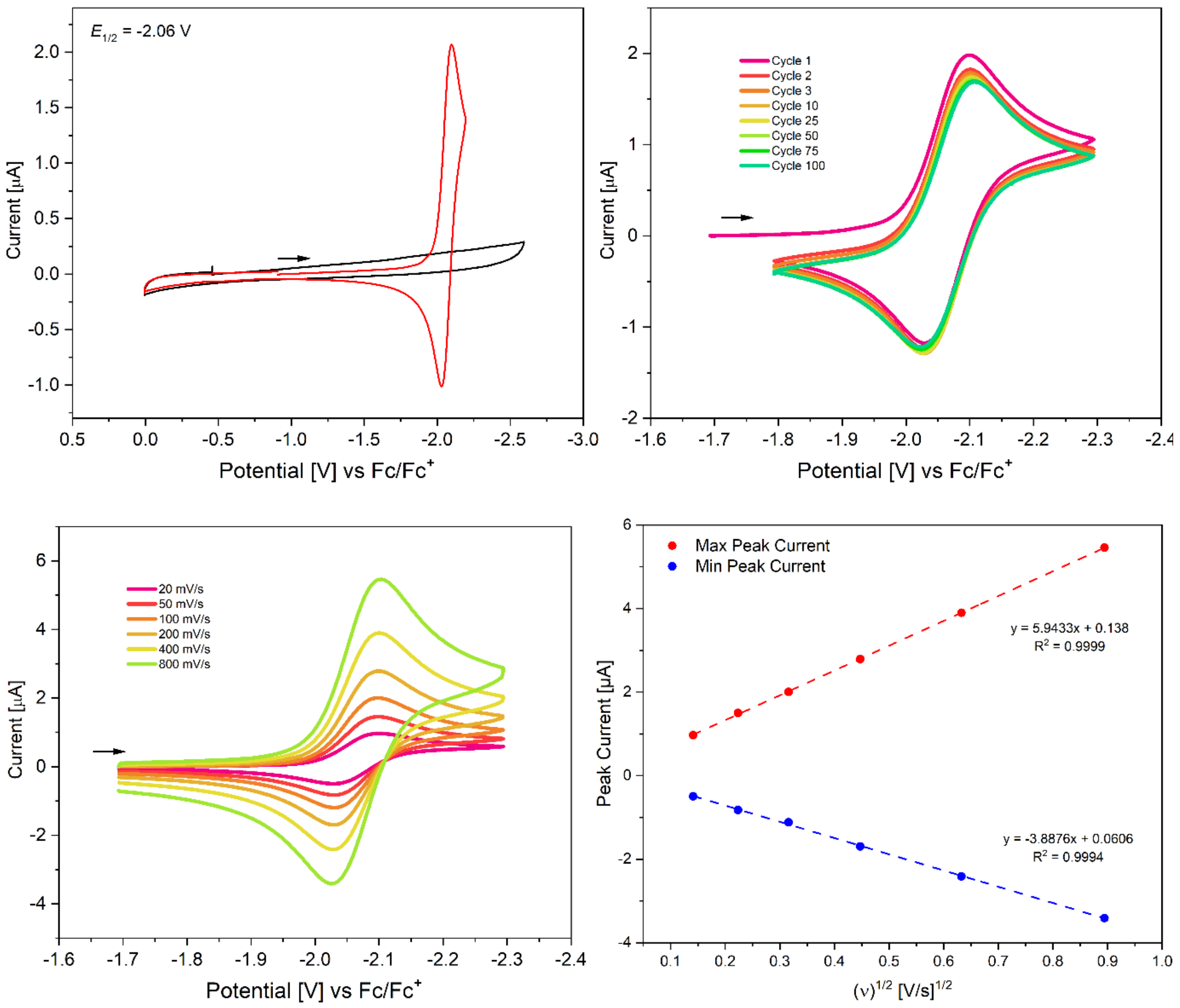

Figure S29: Cyclic voltammogram (top left), cycling (top right) and scan rate study (bottom) of **1-*t*Bu** (1.0 mM) with 0.10 M [(*n*-Bu)$_4$N][PF$_6$] as supporting electrolyte in MeCN. Referenced against Fc/Fc$^+$. The full scan (red) was recorded at 100 mV/s and is shown against the background (black) of 0.10 M [(*n*-Bu)$_4$N][PF$_6$] in MeCN.

Compound **1-*i*Pent** in MeCN:

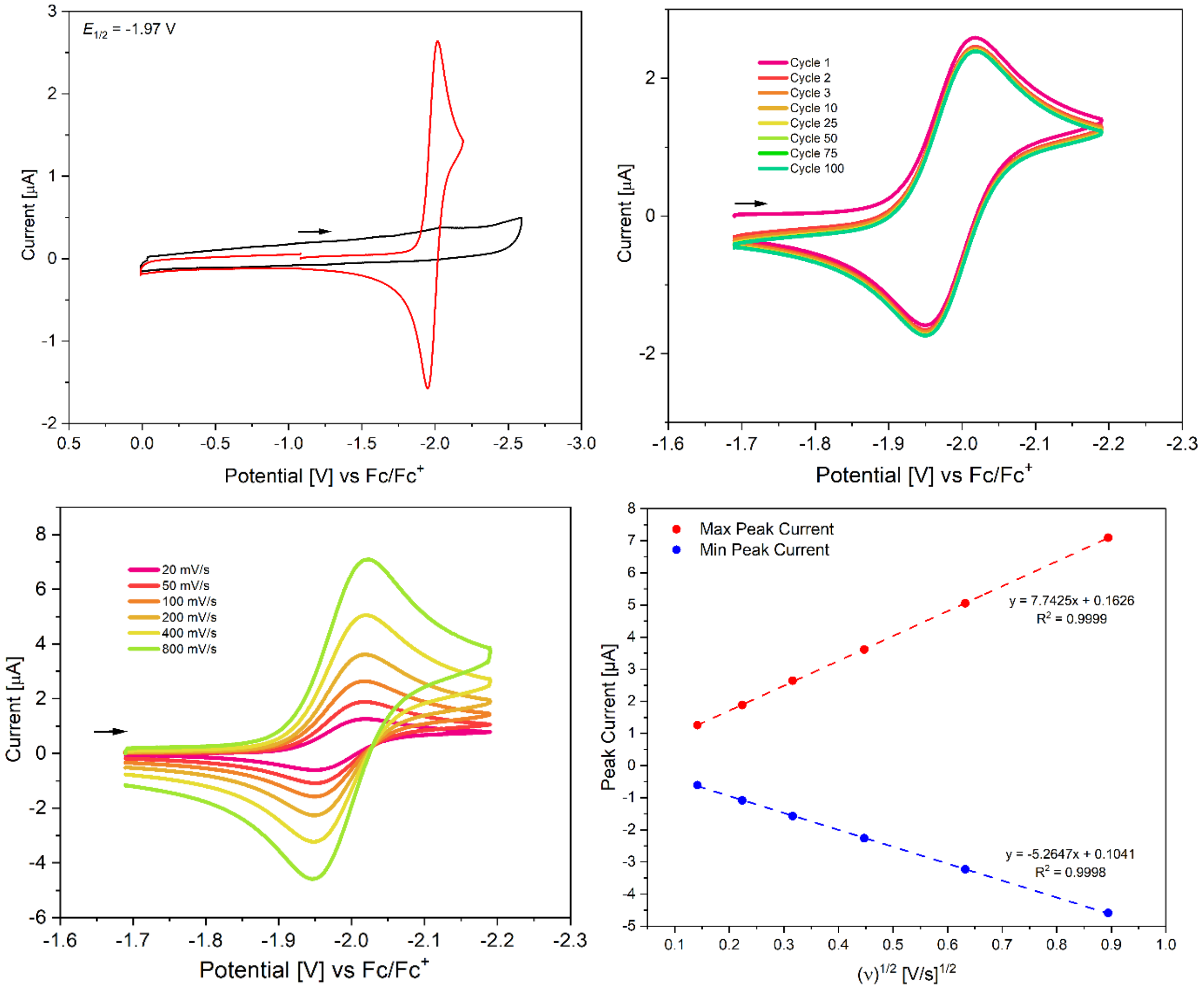

Figure S30: Cyclic voltammogram (top left), cycling (top right) and scan rate study (bottom) of **1-*i*Pent** (1.0 mM) with 0.10 M [(*n*-Bu)$_4$N][PF$_6$] as supporting electrolyte in MeCN. Referenced against Fc/Fc$^+$. The full scan (red) was recorded at 100 mV/s and is shown against the background (black) of 0.10 M [(*n*-Bu)$_4$N][PF$_6$] in MeCN.

Compound **1-Cy** in MeCN:

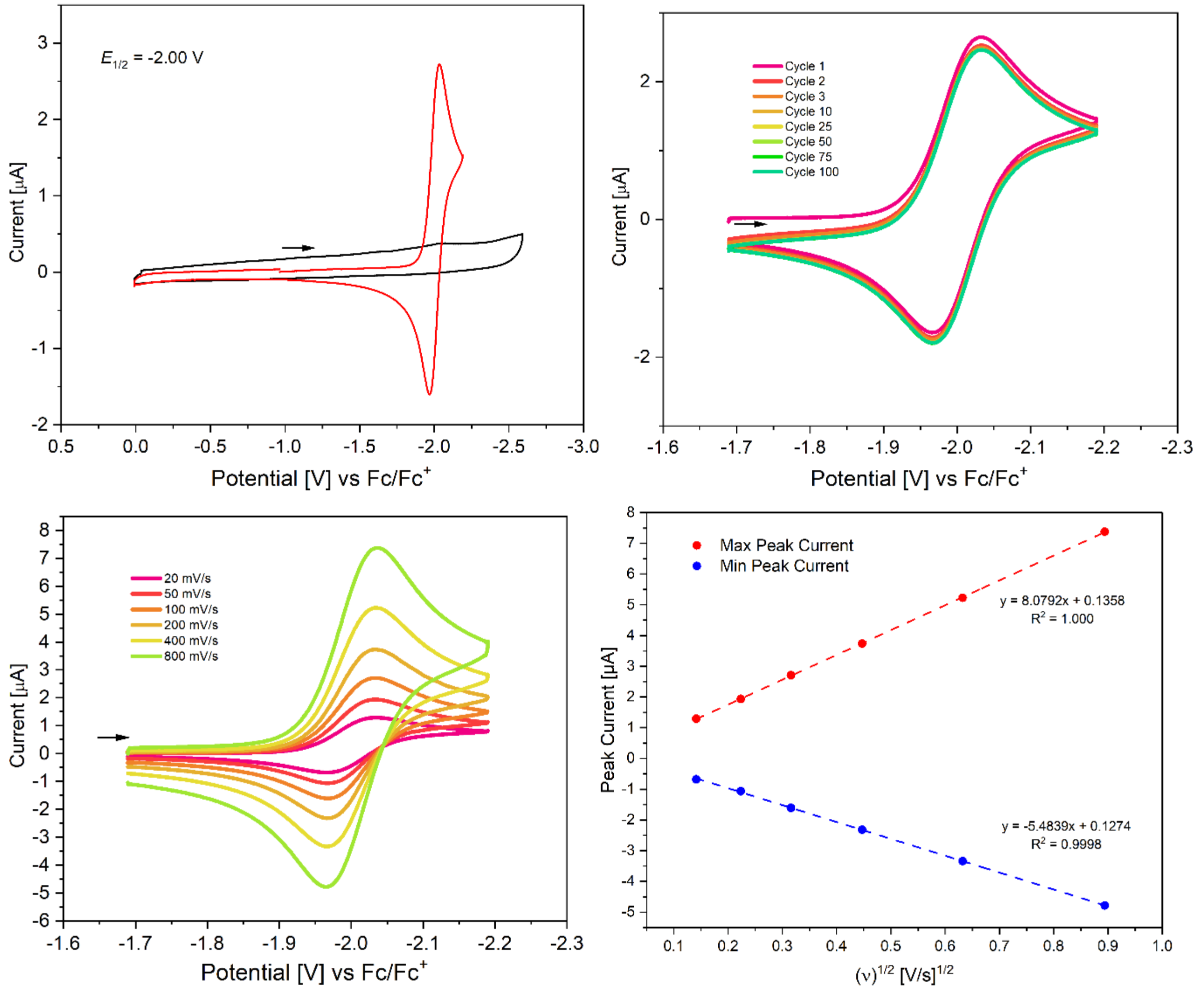

Figure S31: Cyclic voltammogram (top left), cycling (top right) and scan rate study (bottom) of **1-Cy** (1.0 mM) with 0.10 M [(n-Bu)$_4$N][PF$_6$] as supporting electrolyte in MeCN. Referenced against Fc/Fc$^+$. The full scan (red) was recorded at 100 mV/s and is shown against the background (black) of 0.10 M [(n-Bu)$_4$N][PF$_6$] in MeCN.

Compound **1-Bn** in MeCN:

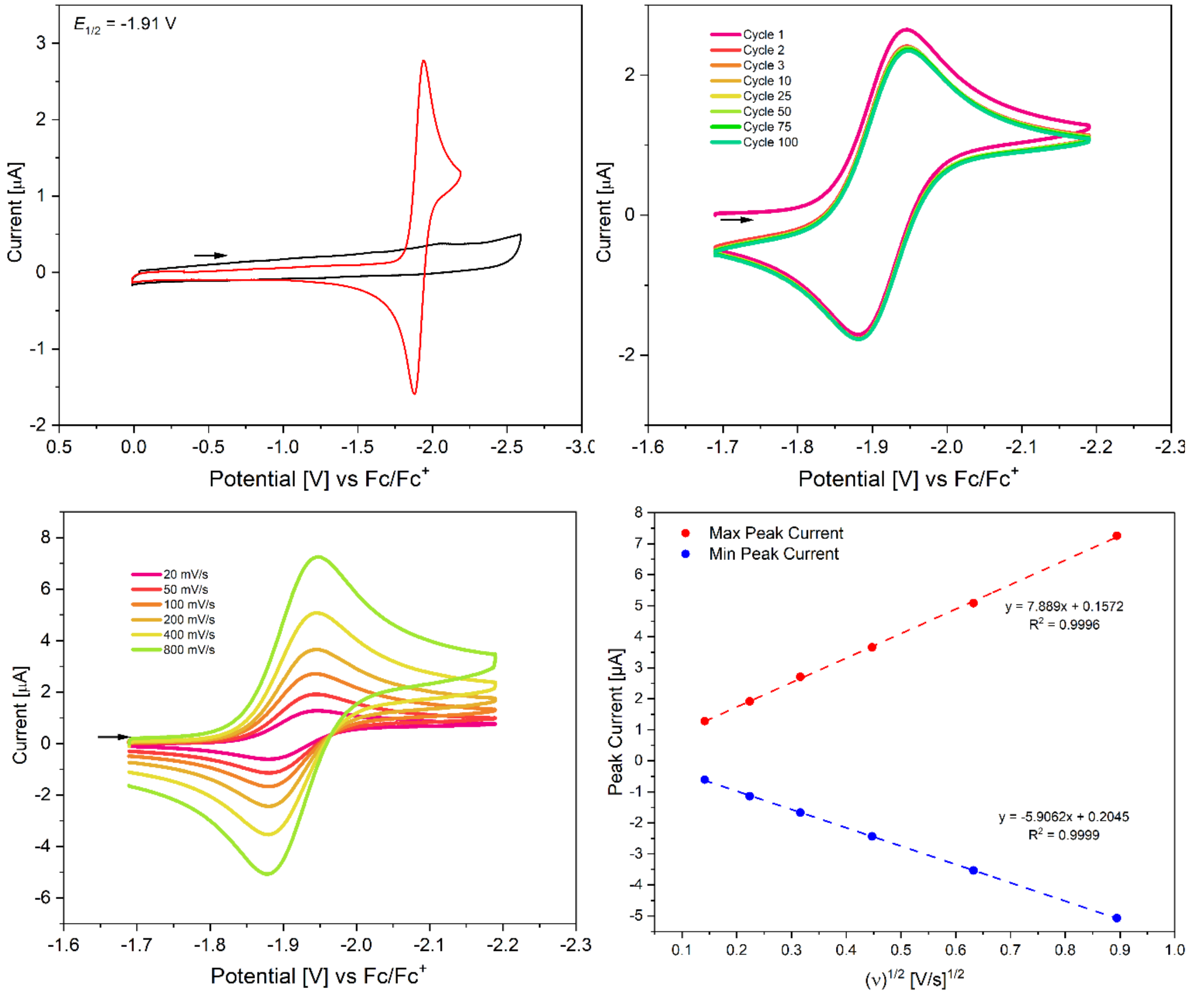

Figure S32: Cyclic voltammogram (top left), cycling (top right) and scan rate study (bottom) of **1-Bn** (1.0 mM) with 0.10 M $[(n\text{-Bu})_4N][PF_6]$ as supporting electrolyte in MeCN. Referenced against Fc/Fc^+. The full scan (red) was recorded at 100 mV/s and is shown against the background (black) of 0.10 M $[(n\text{-Bu})_4N][PF_6]$ in MeCN.

Compound **1-EH** in MeCN:

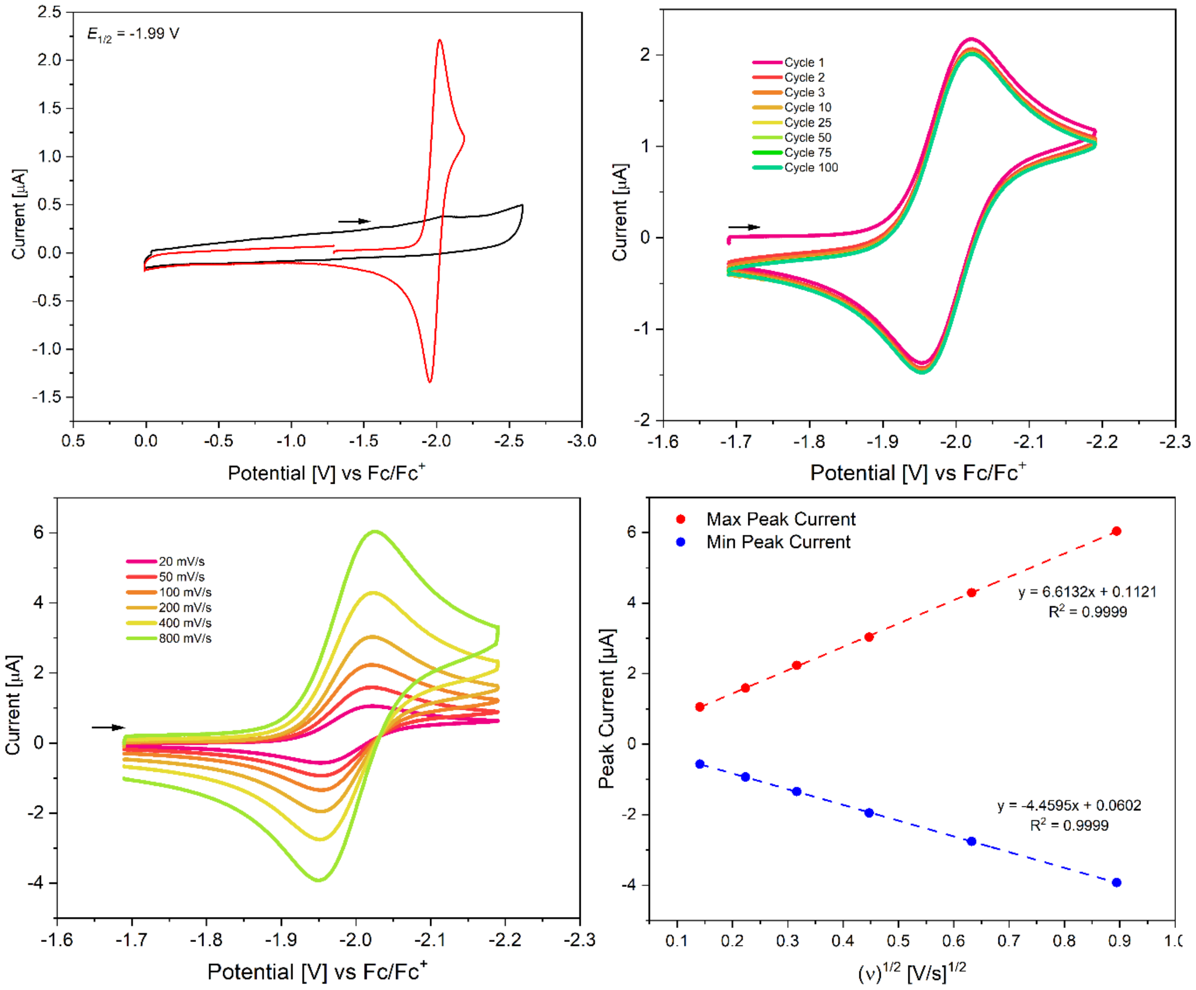

Figure S33: Cyclic voltammogram (top left), cycling (top right) and scan rate study (bottom) of **1-EH** (1.0 mM) with 0.10 M [(*n*-Bu)$_4$N][PF$_6$] as supporting electrolyte in MeCN. Referenced against Fc/Fc$^+$. The full scan (red) was recorded at 100 mV/s and is shown against the background (black) of 0.10 M [(*n*-Bu)$_4$N][PF$_6$] in MeCN.

Compound **1-*i*Pr** in MeCN/DMF (3:2, *v*:*v*):

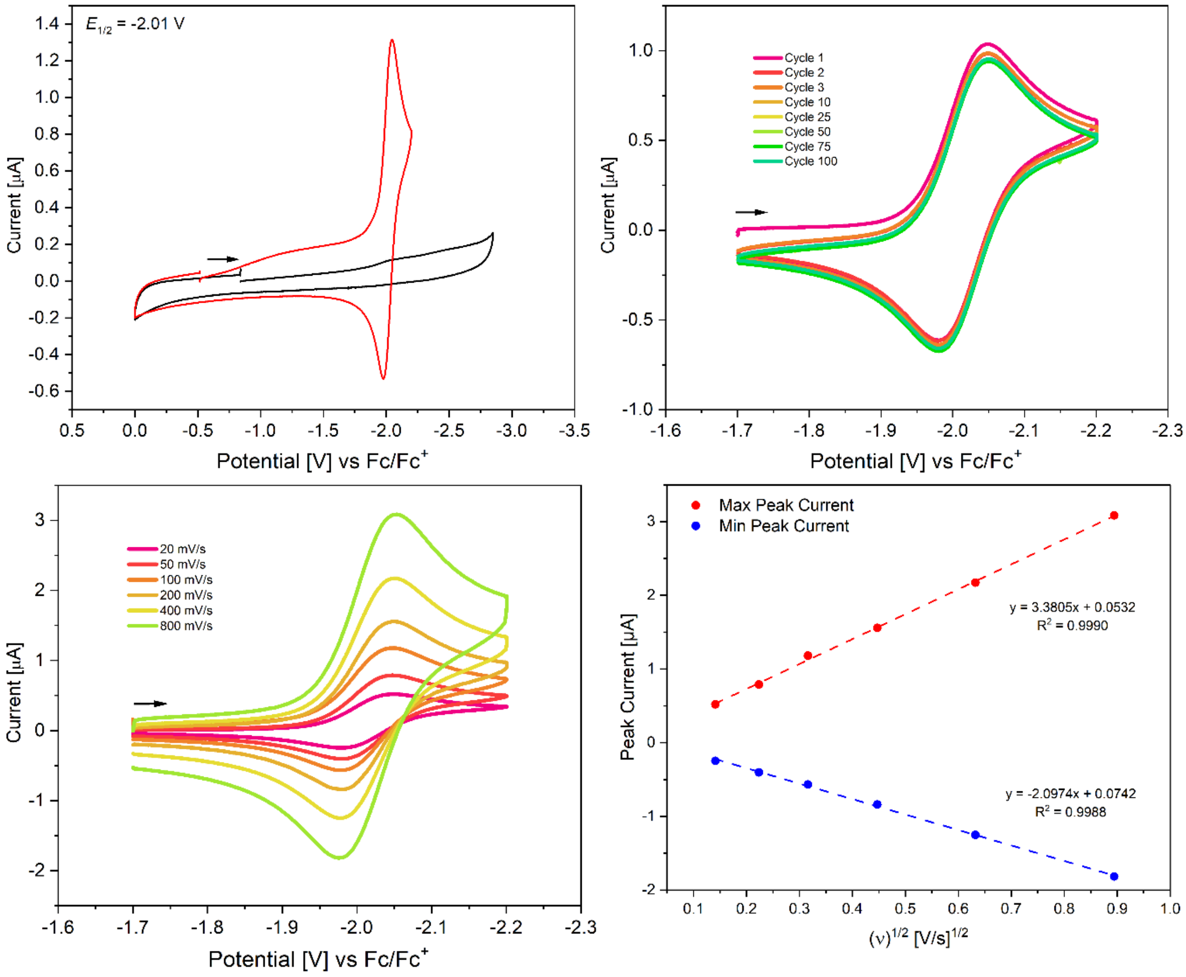

Figure S34: Cyclic voltammogram (top left), cycling (top right) and scan rate study (bottom) of **1-*i*Pr** (1.0 mM) with 0.10 M [(*n*-Bu)$_4$N][PF$_6$] as supporting electrolyte in MeCN/DMF (3:2, *v*:*v*). Referenced against Fc/Fc$^+$. The full scan (red) was recorded at 100 mV/s and is shown against the background (black) of 0.10 M [(*n*-Bu)$_4$N][PF$_6$] in MeCN/DMF (3:2, *v*:*v*).

Supporting Electrolyte Study for **1-Me**:

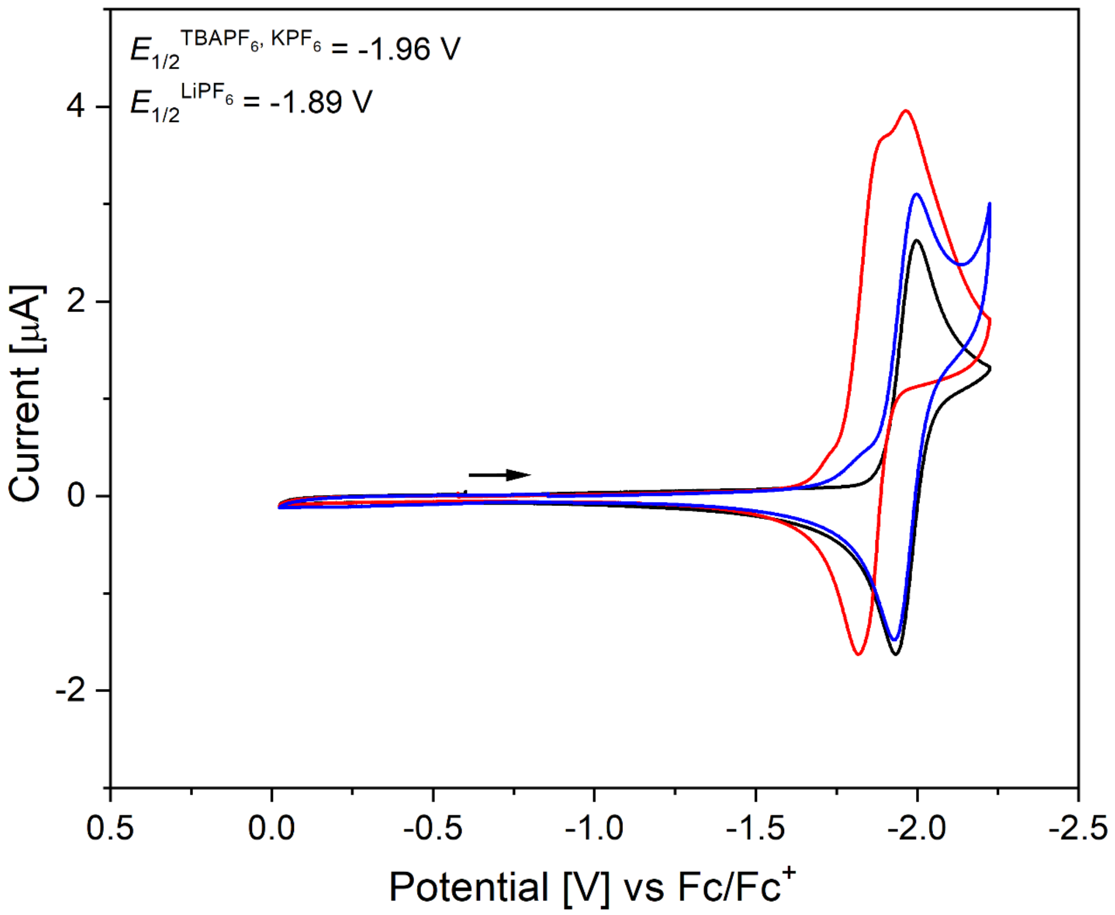

Figure S35: Cyclic voltammograms of **1-Me** (1.0 mM) in MeCN with different supporting electrolytes: 0.10 M [(*n*-Bu)$_4$N][PF$_6$] (black), 0.10 M [K][PF$_6$] (blue) and 0.10 M [Li][PF$_6$] (red). All data was referenced against Fc/Fc$^+$ and recorded at a scan rate of 100 mV/s.

Additional Electrochemical Data

Thiophene in MeCN

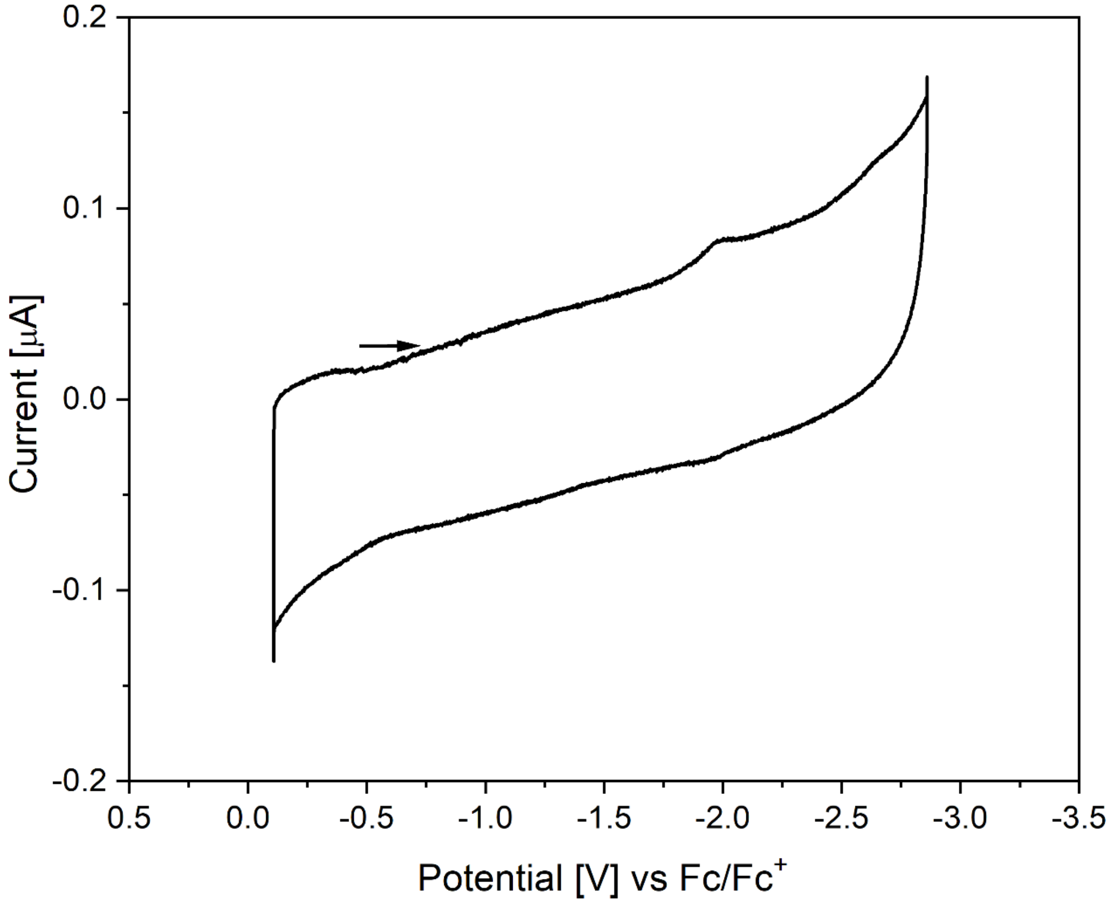

Figure S36: Cyclic voltammogram of thiophene (1.0 mM) with 0.10 M [(*n*-Bu)$_4$N][PF_6] as supporting electrolyte in MeCN. Referenced against Fc/Fc$^+$. Only the reductive window was recorded due to electropolymerization on the working electrode at positive potentials.

Thiophene-2,5-Dicarboxylic Acid in 3M aqueous KCl

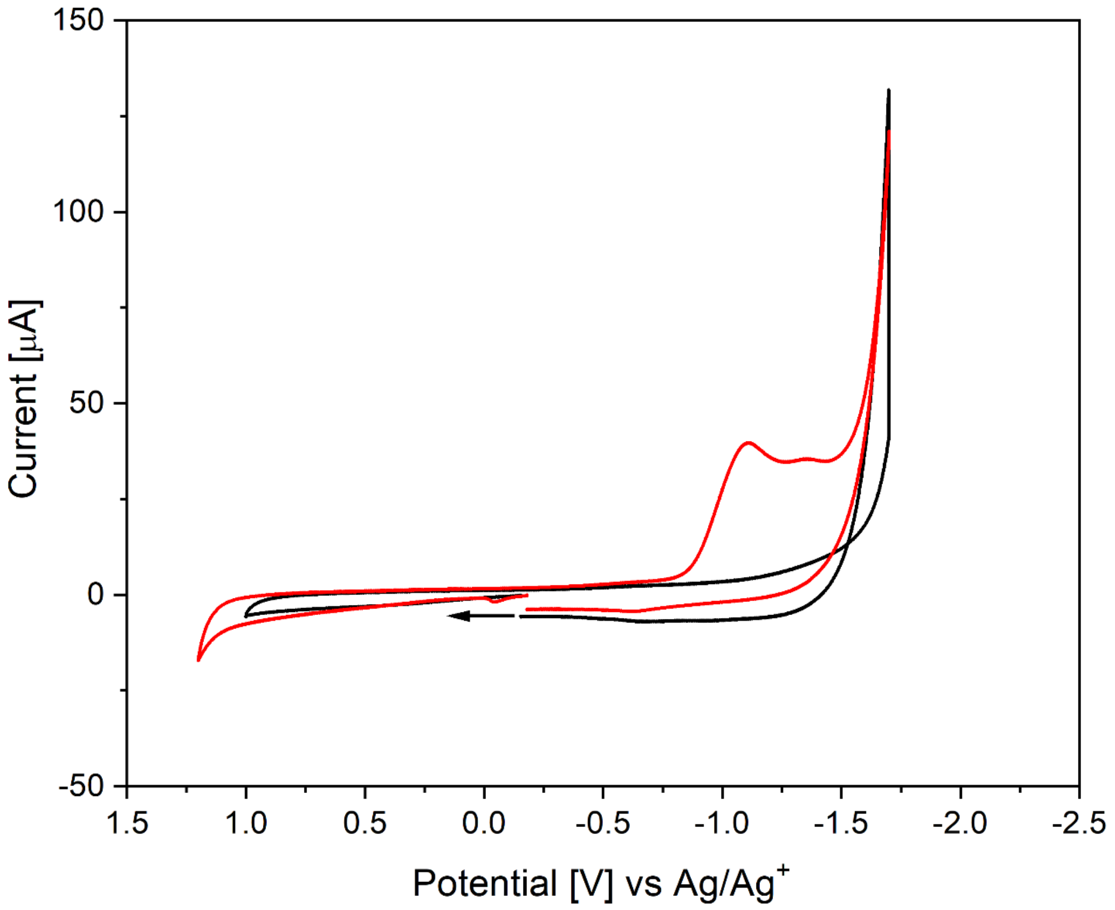

Figure S37: Cyclic voltammogram of thiophene-2,5-dicarboxylic acid (1.0 mM) with 3 M KCl as supporting electrolyte in water using a glassy carbon working electrode and a platinum wire counter electrode. Referenced against an Ag/Ag$^+$ pseudo reference electrode. The full scan (red) was recorded at 100 mV/s and is shown against the background (black) of 3 M KCl in water.

Thiophene-2,5-Dicarboxylic Acid in 3M aqueous KOH

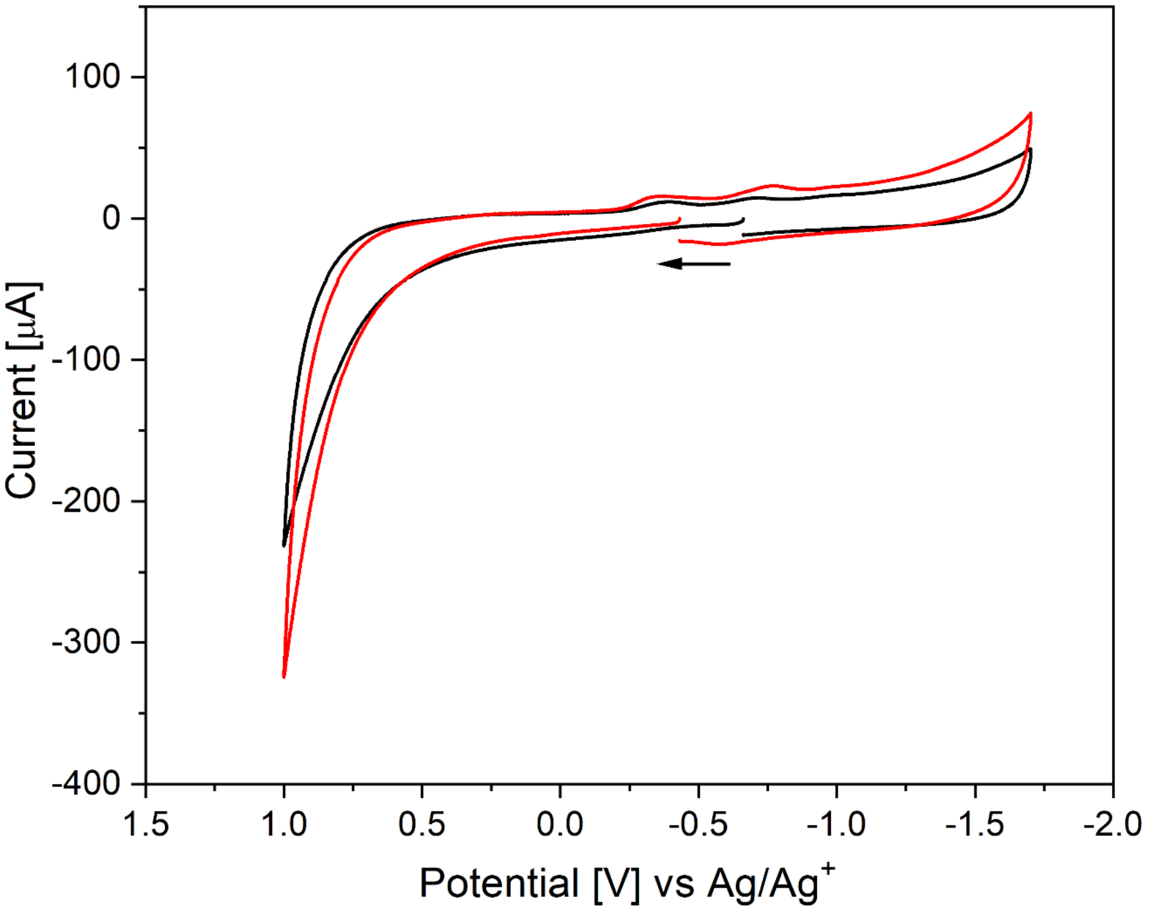

Figure S38: Cyclic voltammogram of thiophene-2,5-dicarboxylic acid (1.0 mM) with 3 M KOH as supporting electrolyte in water using a glassy carbon working electrode and a platinum wire counter electrode. Referenced against an Ag/Ag^+ pseudo reference electrode. The full scan (red) was recorded at 100 mV/s and is shown against the background (black) of 3 M KOH in water.

Galvanostatic Charge-Discharge Cycling

1-Me in MeCN

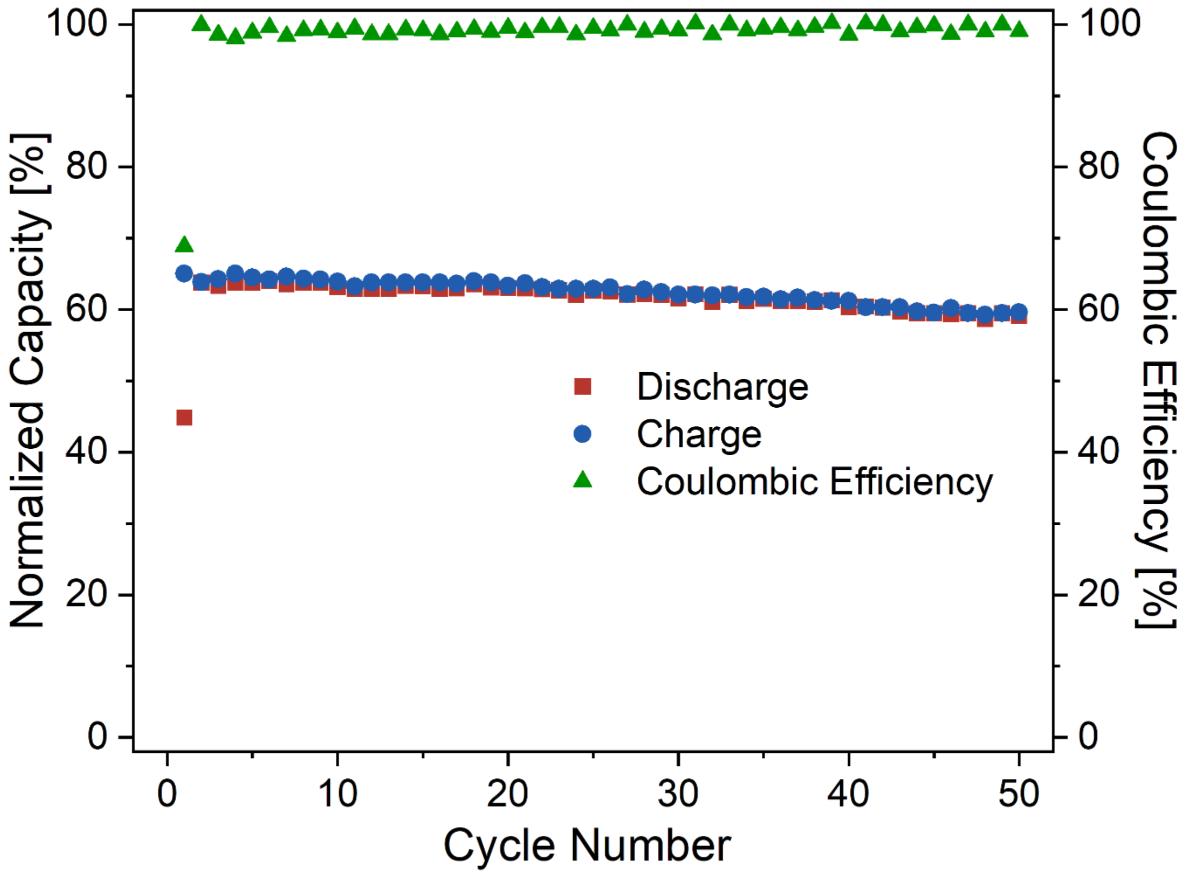

Figure S39: Normalized capacity and coulombic efficiency versus cycle number of **1-Me** with 0.1 M [(n-Bu)$_4$N][PF_6] as supporting electrolyte in MeCN in a symmetrical H-cell experiment. The experiment was performed as described above at 0.5 mA (3C) with voltaic cutoffs of −2.05 and −1.65 V (vs internal reference) over the course of 20.0 hours, giving an average 0.38% capacity fade per hour.

1-Et in MeCN

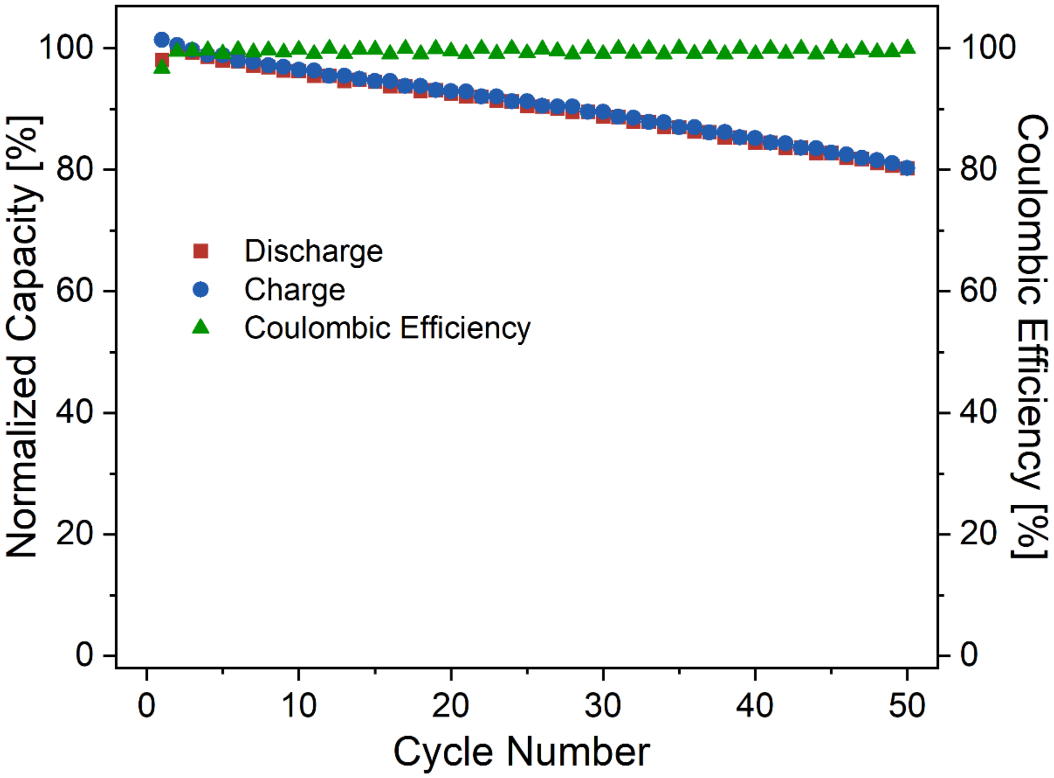

Figure S40: Normalized capacity and coulombic efficiency versus cycle number of **1-Et** with 0.1 M [(*n*-Bu)$_4$N][PF$_6$] as supporting electrolyte in MeCN in a symmetrical H-cell experiment. The experiment was performed as described above at 0.5 mA (3C) with voltaic cutoffs of −2.09 and −1.69 V (vs internal reference) over the course of 29.8 hours, giving an average 0.69% capacity fade per hour.

1-*n*Pr in MeCN

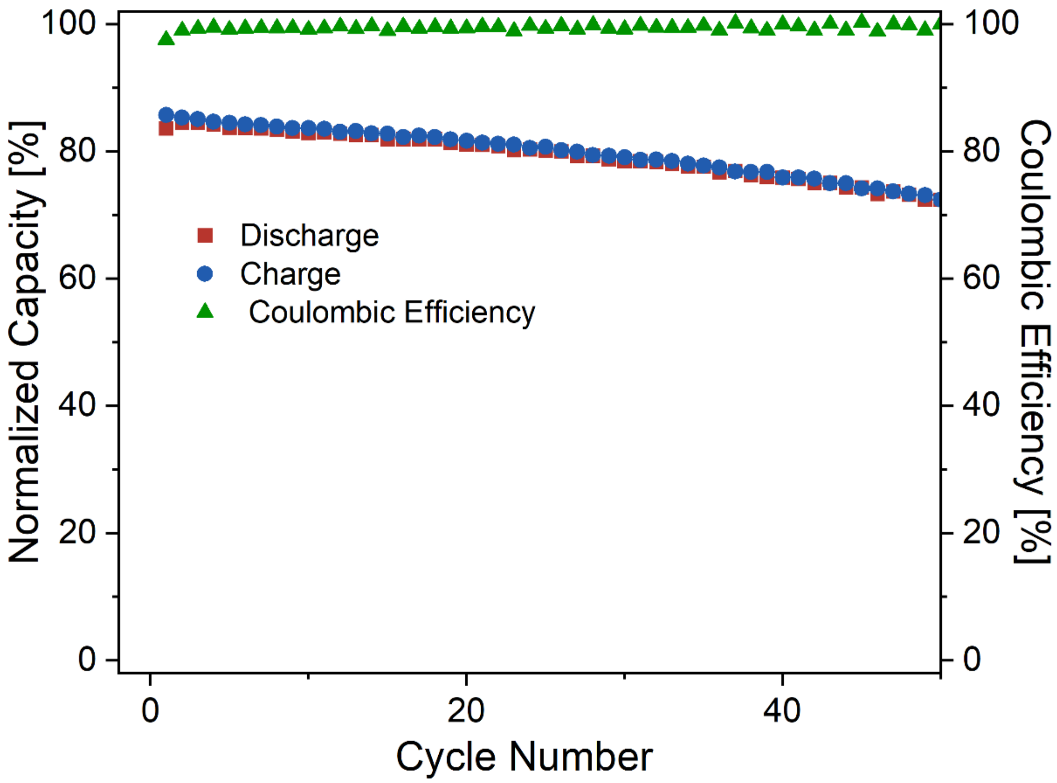

Figure S41: Normalized capacity and coulombic efficiency versus cycle number of **1-*n*Pr** with 0.1 M [(*n*-Bu)$_4$N][PF$_6$] as supporting electrolyte in MeCN in a symmetrical H-cell experiment. The experiment was performed as described above at 0.5 mA (3C) with voltaic cutoffs of −1.90 and −1.50 V (vs internal reference) over the course of 20.7 hours, giving an average 0.52% capacity fade per hour.

1-*i*Pr in MeCN

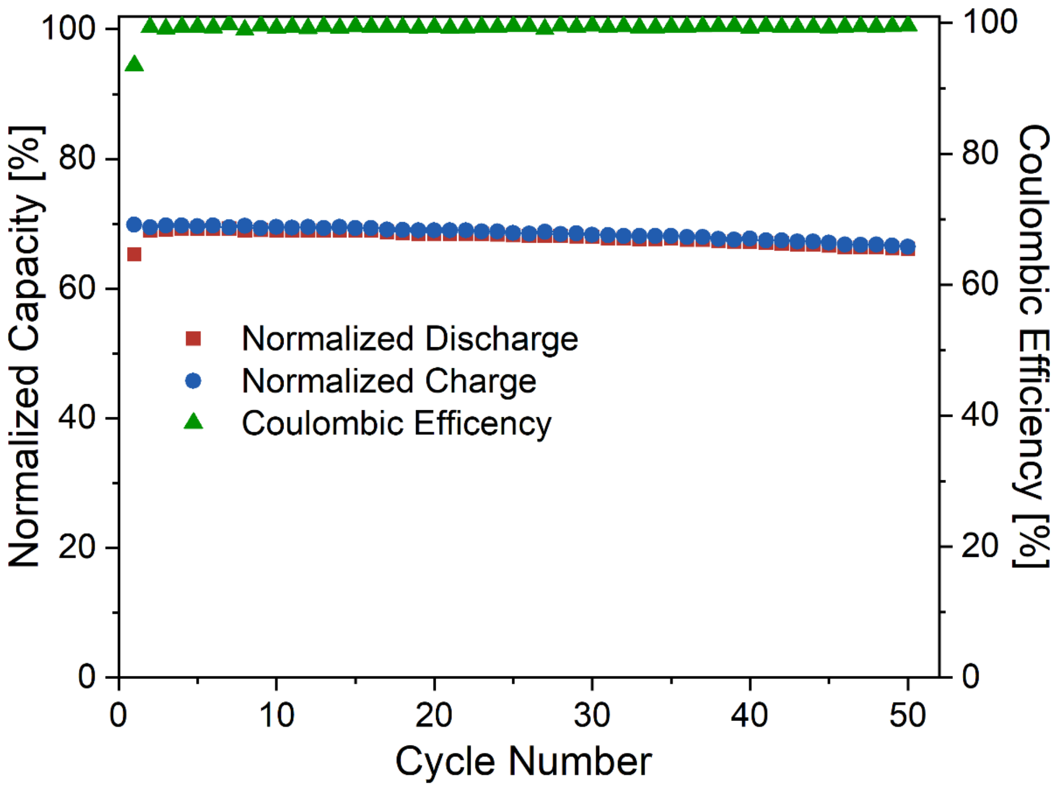

Figure S42: Normalized capacity and coulombic efficiency versus cycle number of **1-*i*Pr** with 0.1 M [(*n*-Bu)$_4$N][PF$_6$] as supporting electrolyte in MeCN in a symmetrical H-cell experiment. The experiment was performed as described above at 0.5 mA (3C) with voltaic cutoffs of −2.07 and −1.67 V (vs internal reference) over the course of 22.0 hours, giving an average 0.20% capacity fade per hour.

1-*i*Bu in MeCN

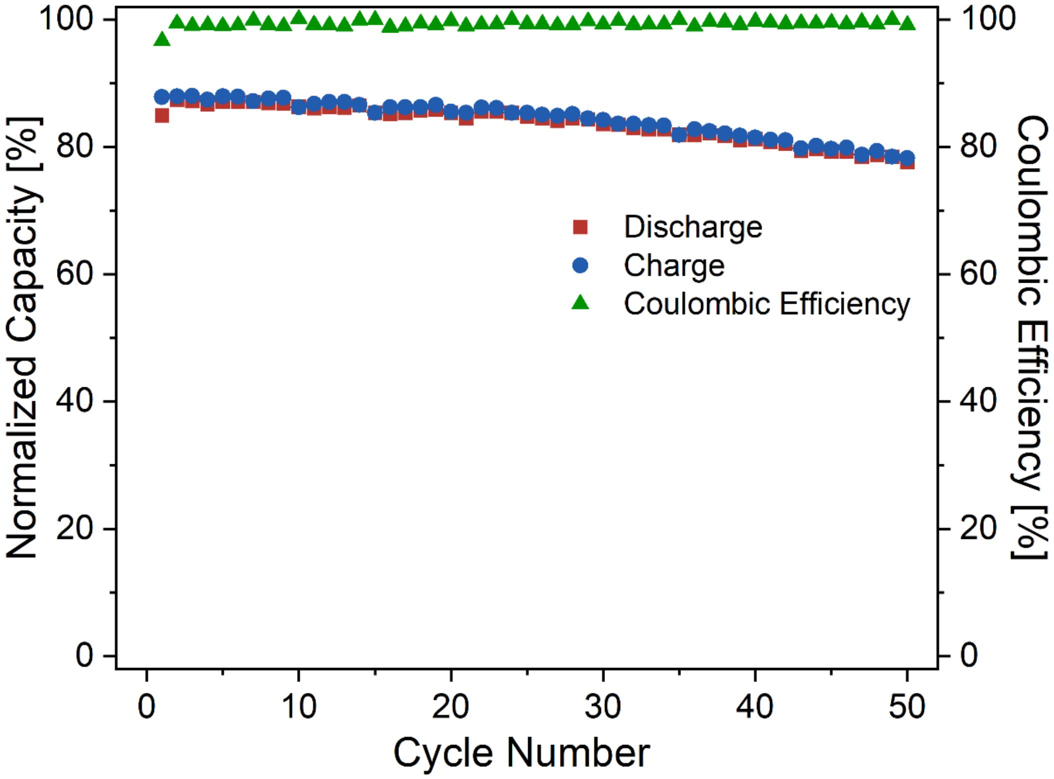

Figure S43: Normalized capacity and coulombic efficiency versus cycle number of **1-*i*Bu** with 0.1 M [(*n*-Bu)$_4$N][PF$_6$] as supporting electrolyte in MeCN in a symmetrical H-cell experiment. The experiment was performed as described above at 0.5 mA (3C) with voltaic cutoffs of −2.05 and −1.65 V (vs internal reference) over the course of 27.1 hours, giving an average 0.43% capacity fade per hour.

1-*t*Bu in MeCN

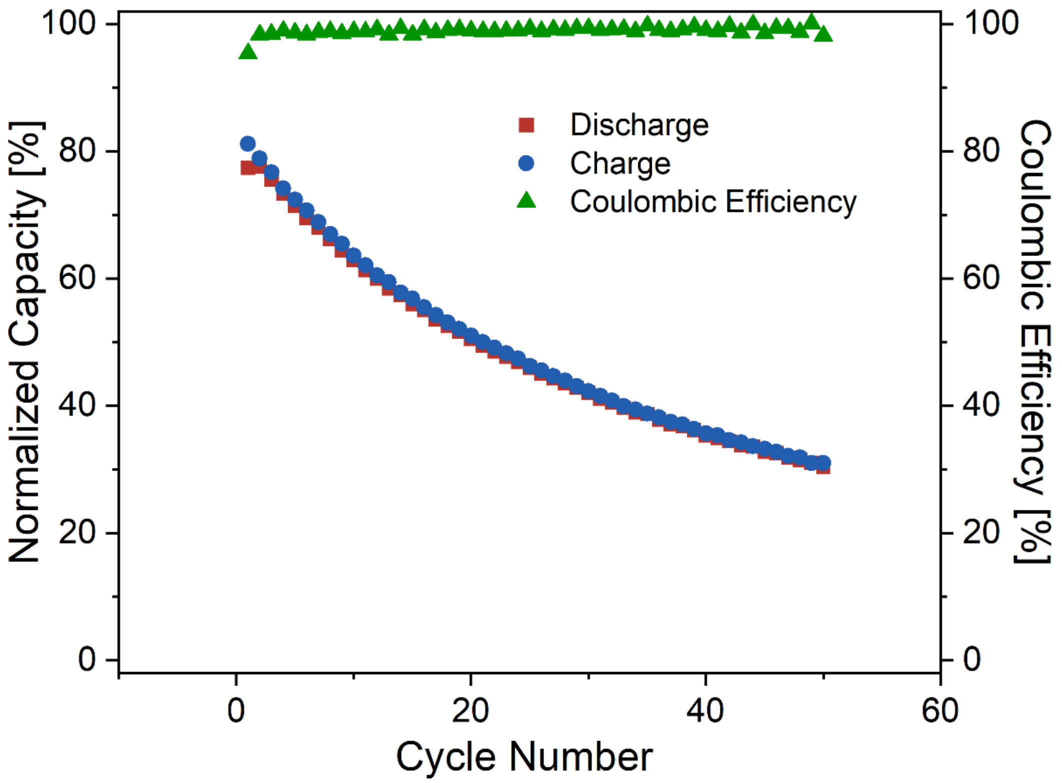

Figure S44: Normalized capacity and coulombic efficiency versus cycle number of **1-*t*Bu** with 0.1 M [(*n*-Bu)$_4$N][PF$_6$] as supporting electrolyte in MeCN in a symmetrical H-cell experiment. The experiment was performed as described above at 0.5 mA (3C) with voltaic cutoffs of −2.09 and −1.69 V (vs internal reference) over the course of 15.8 hours, giving an average 3.85% capacity fade per hour.

1-*i*Pent in MeCN

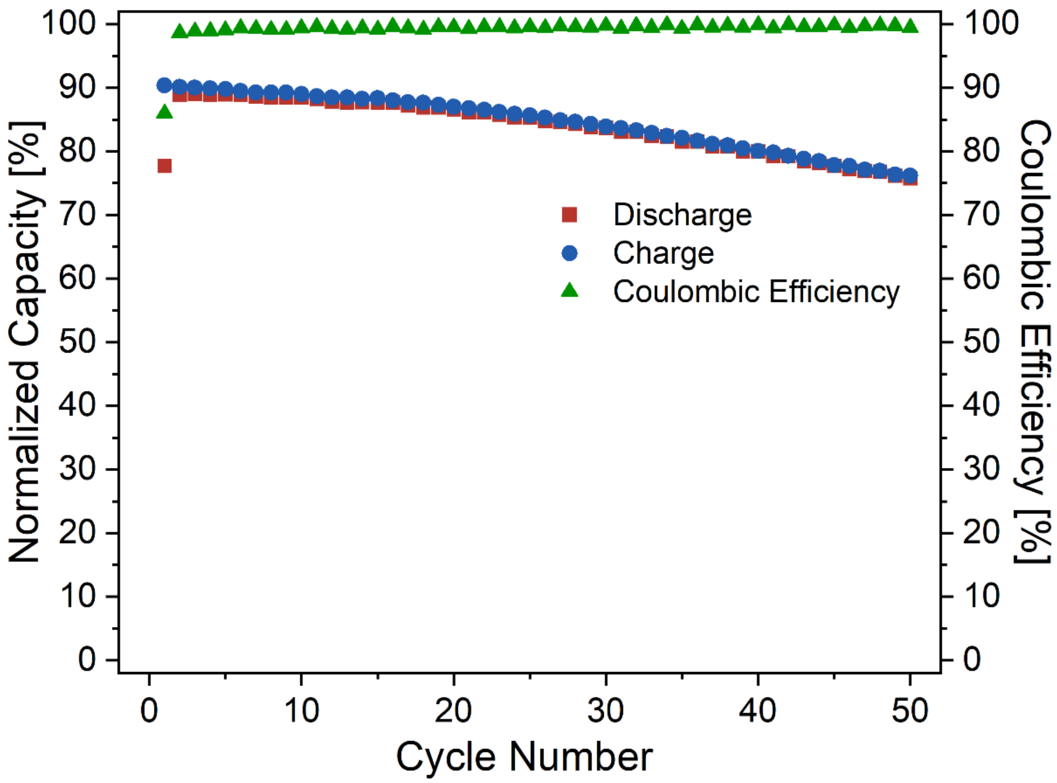

Figure S45: Normalized capacity and coulombic efficiency versus cycle number of **1-*i*Pent** with 0.1 M [(*n*-Bu)$_4$N][PF$_6$] as supporting electrolyte in MeCN in a symmetrical H-cell experiment. The experiment was performed as described above at 0.5 mA (3C) with voltaic cutoffs of −2.10 and −1.70 V (vs internal reference) over the course of 30.4 hours, giving an average 0.48% capacity fade per hour.

1-Cy in MeCN

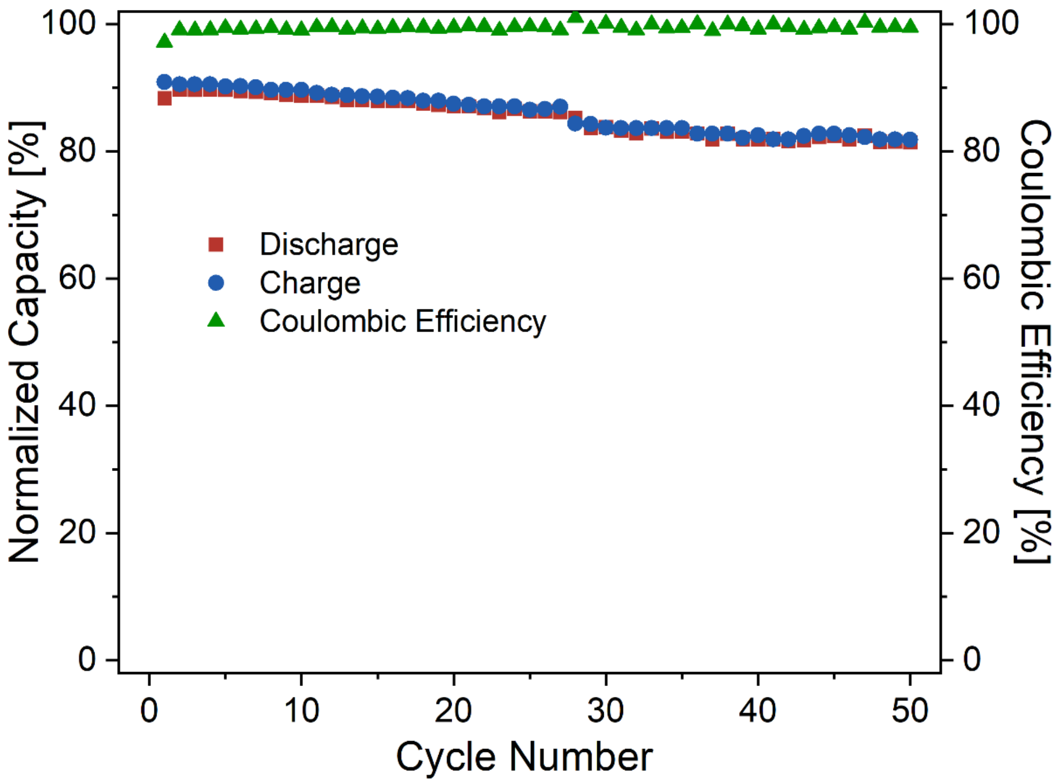

Figure S46: Normalized capacity and coulombic efficiency versus cycle number of **1-Cy** with 0.1 M [(*n*-Bu)$_4$N][PF$_6$] as supporting electrolyte in MeCN in a symmetrical H-cell experiment. The experiment was performed as described above at 0.5 mA (3C) with voltaic cutoffs of −2.10 and −1.70 V (vs internal reference) over the course of 27.6 hours, giving an average 0.33% capacity fade per hour.

1-Bn in MeCN

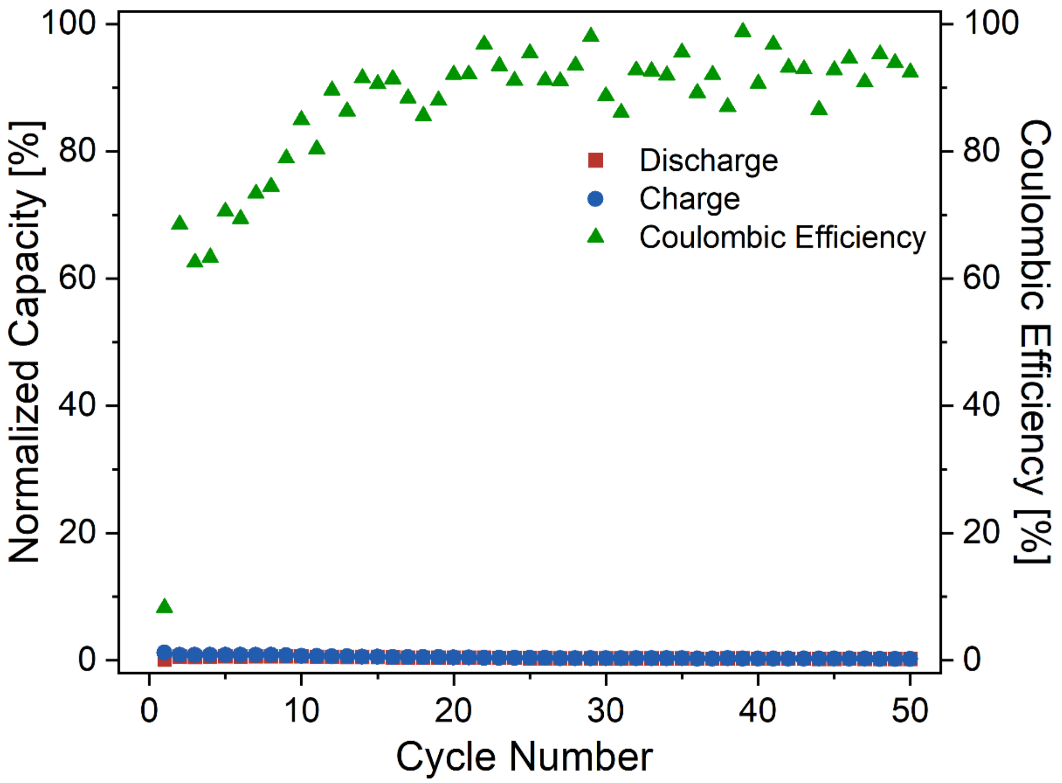

Figure S47: Normalized capacity and coulombic efficiency versus cycle number of **1-Bn** with 0.1 M [(n-Bu)$_4$N][PF$_6$] as supporting electrolyte in MeCN in a symmetrical H-cell experiment. The experiment was performed as described above at 0.5 mA (3C) with voltaic cutoffs of −2.00 and −1.60 V (vs internal reference). After the initial charge, >99% of the initial capacity was lost.

1-EH in MeCN

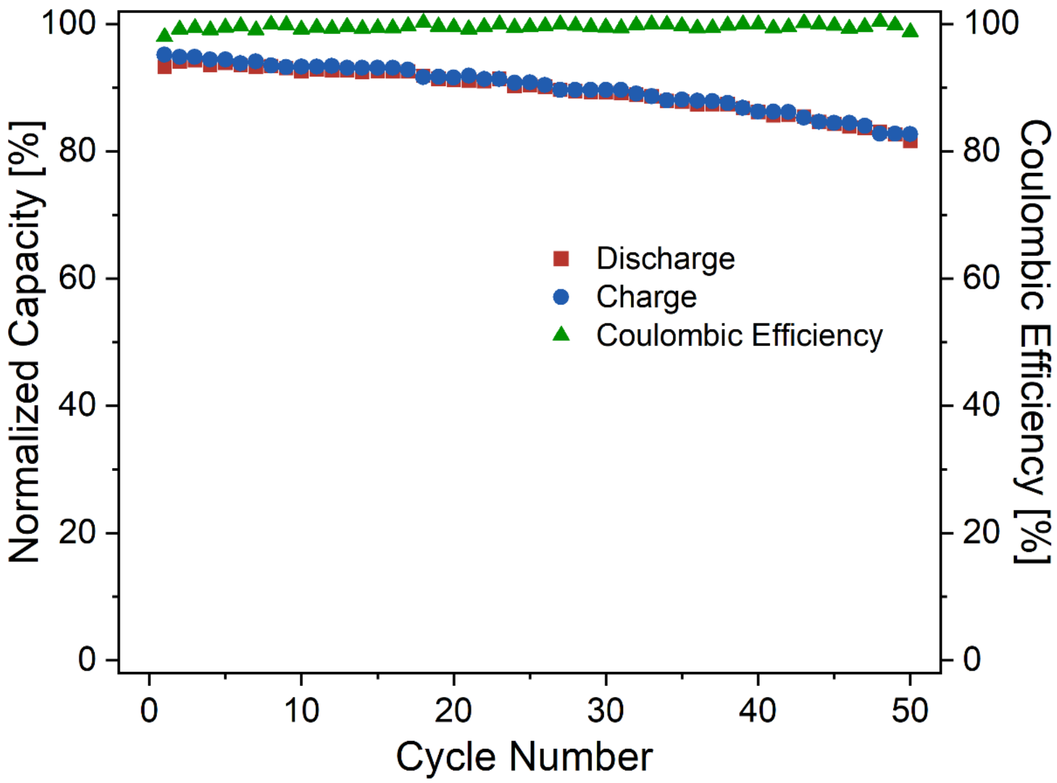

Figure S48: Normalized capacity and coulombic efficiency versus cycle number of **1-EH** with 0.1 M [(*n*-Bu)$_4$N][PF$_6$] as supporting electrolyte in MeCN in a symmetrical H-cell experiment. The experiment was performed as described above at 0.5 mA (3C) with voltaic cutoffs of −2.10 and −1.70 V (vs internal reference) over the course of 28.9 hours, giving an average 0.46% capacity fade per hour.

Solvent System Optimization

The influence of solvent on the stability of the redox system **1-*i*Pr/1-*i*Pr$^{•-}$** was examined by preparing a symmetrical H-cell in different solvents and solvent mixtures with 0.1 M [(*n*-Bu)$_4$N][PF$_6$] as supporting electrolyte.

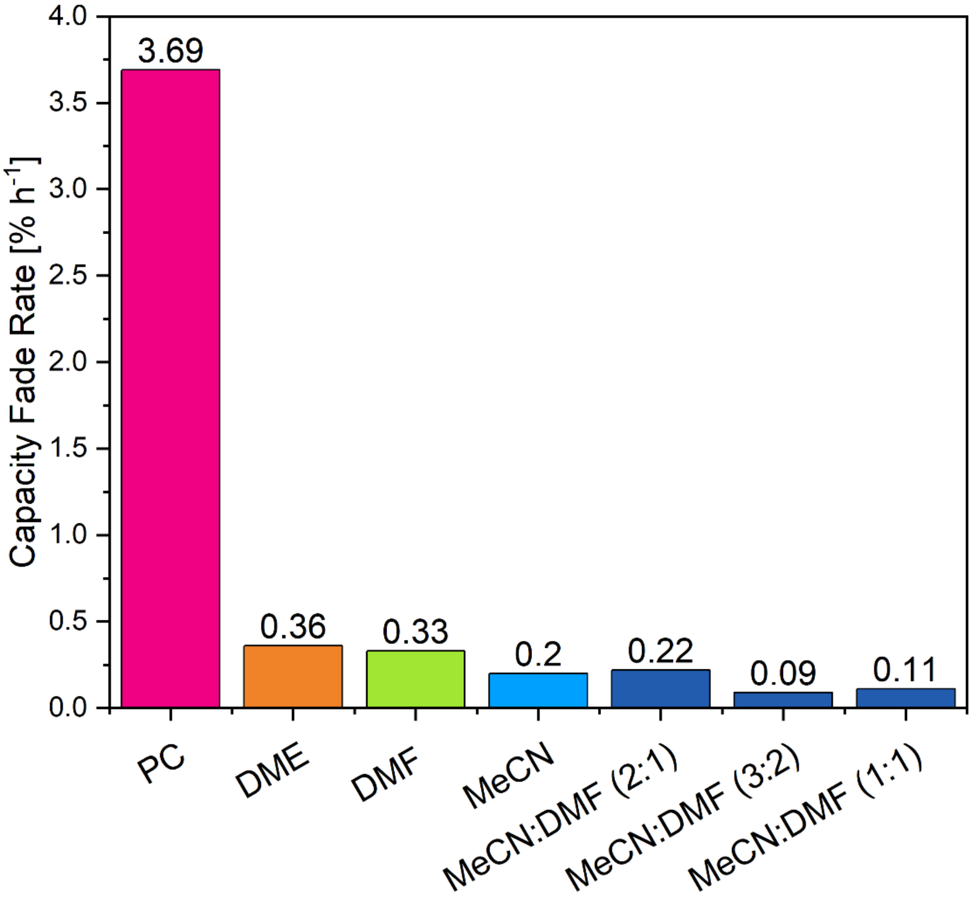

Figure S49: Capacity fade rate (% h^{-1}) of **1-iPr** in various solvent systems, including pure solvents (MeCN, DMF, DME, PC) and MeCN:DMF mixtures at specified volume ratios, measured in a symmetrical H-cell under galvanostatic cycling conditions.

1-*i*Pr in PC

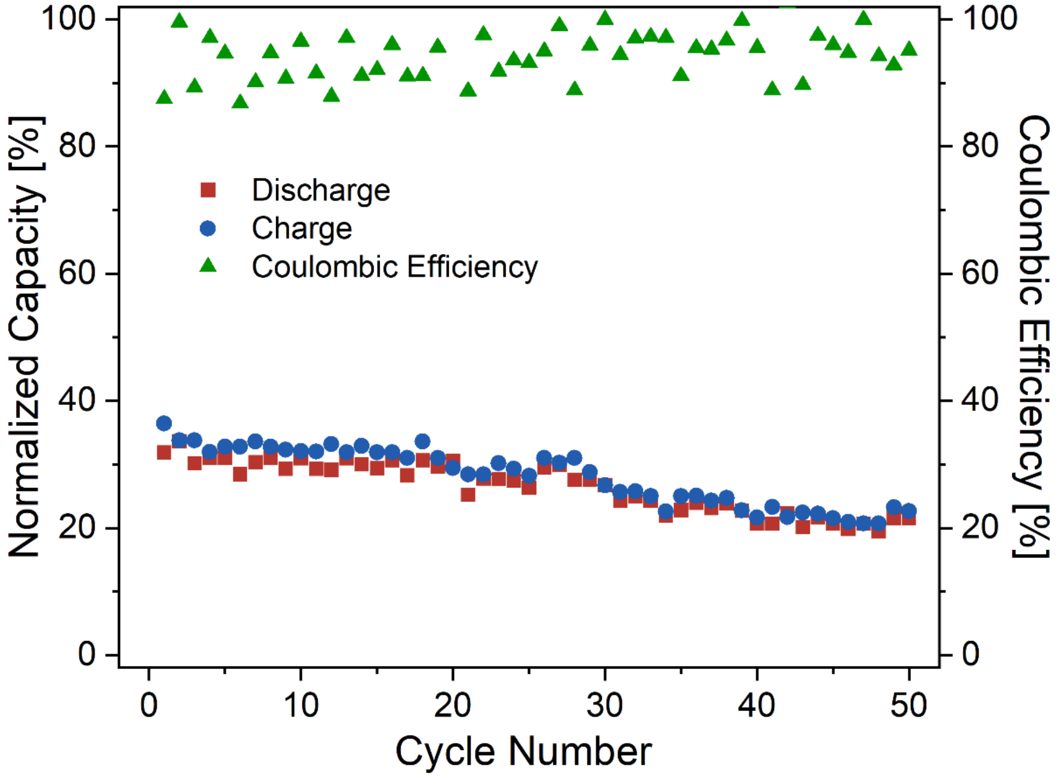

Figure S50: Normalized capacity and coulombic efficiency versus cycle number of **1-*i*Pr** with 0.1 M [(*n*-Bu)$_4$N][PF$_6$] as supporting electrolyte in PC in a symmetrical H-cell experiment. The experiment was performed as described above at 0.5 mA (3C) with voltaic cutoffs of −2.00 and −1.60 V (vs internal reference) over the course of 8.8 hours, giving an average 3.69% capacity fade per hour.

H-cell of **1-*i*Pr** in DME

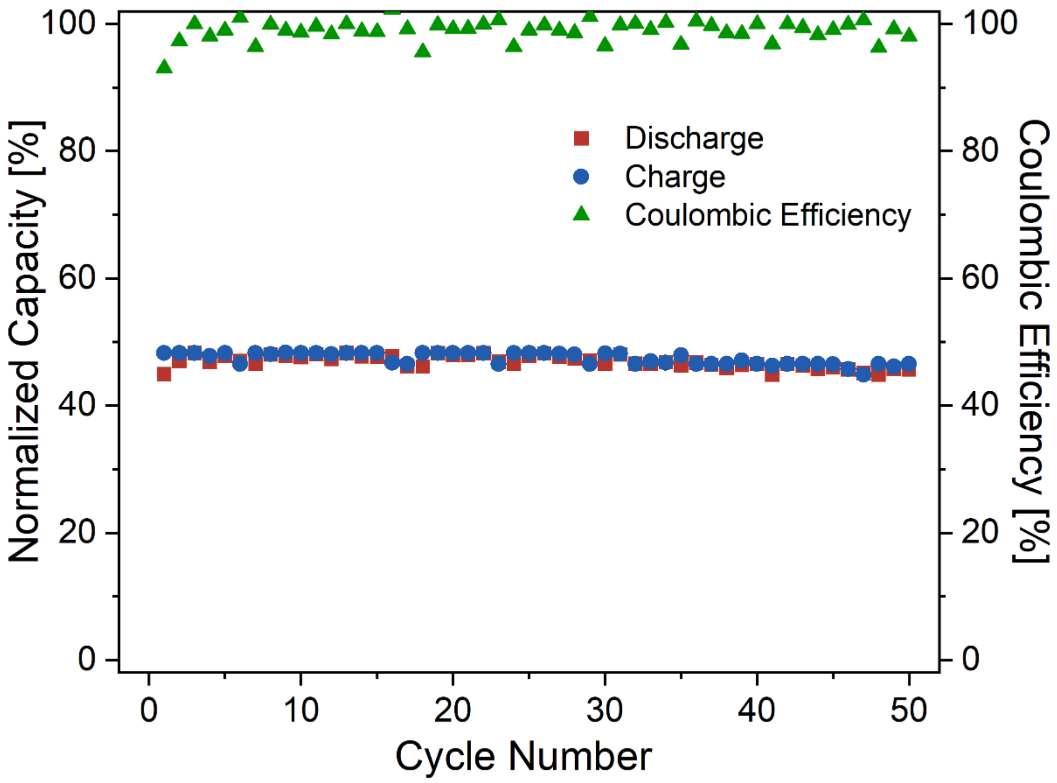

Figure S51: Normalized capacity and coulombic efficiency versus cycle number of **1-*i*Pr** with 0.1 M [(*n*-Bu)$_4$N][PF$_6$] as supporting electrolyte in DME in a symmetrical H-cell experiment. The experiment was performed as described above at 0.5 mA (3C) with voltaic cutoffs of −2.30 and −1.90 V (vs internal reference) over the course of 15.2 hours, giving an average 0.36% capacity fade per hour.

H-cell of **1-*i*Pr** in DMF

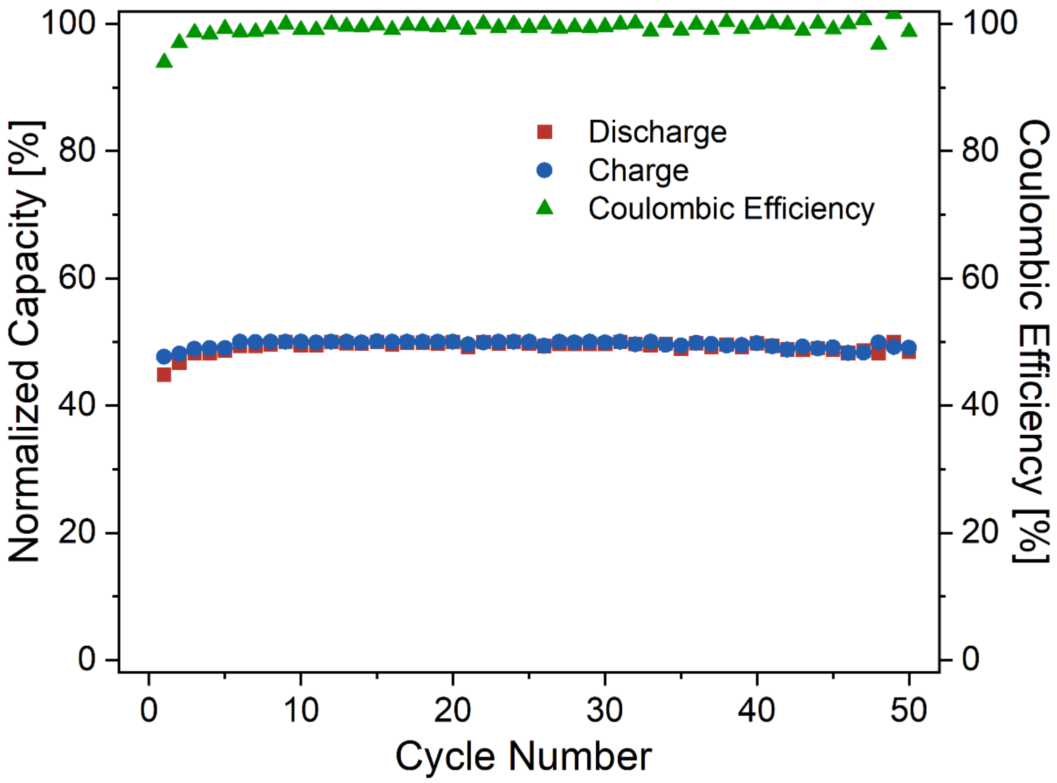

Figure S52: Normalized capacity and coulombic efficiency versus cycle number of **1-*i*Pr** with 0.1 M [(*n*-Bu)$_4$N][PF$_6$] as supporting electrolyte in DMF in a symmetrical H-cell experiment. The experiment was performed as described above at 0.5 mA (3C) with voltaic cutoffs of −2.30 and −1.90 V (vs internal reference) over the course of 15.3 hours, giving an average 0.33% capacity fade per hour.

H-cell of **1-*i*Pr** in MeCN:DMF (1:1, *v*:*v*)

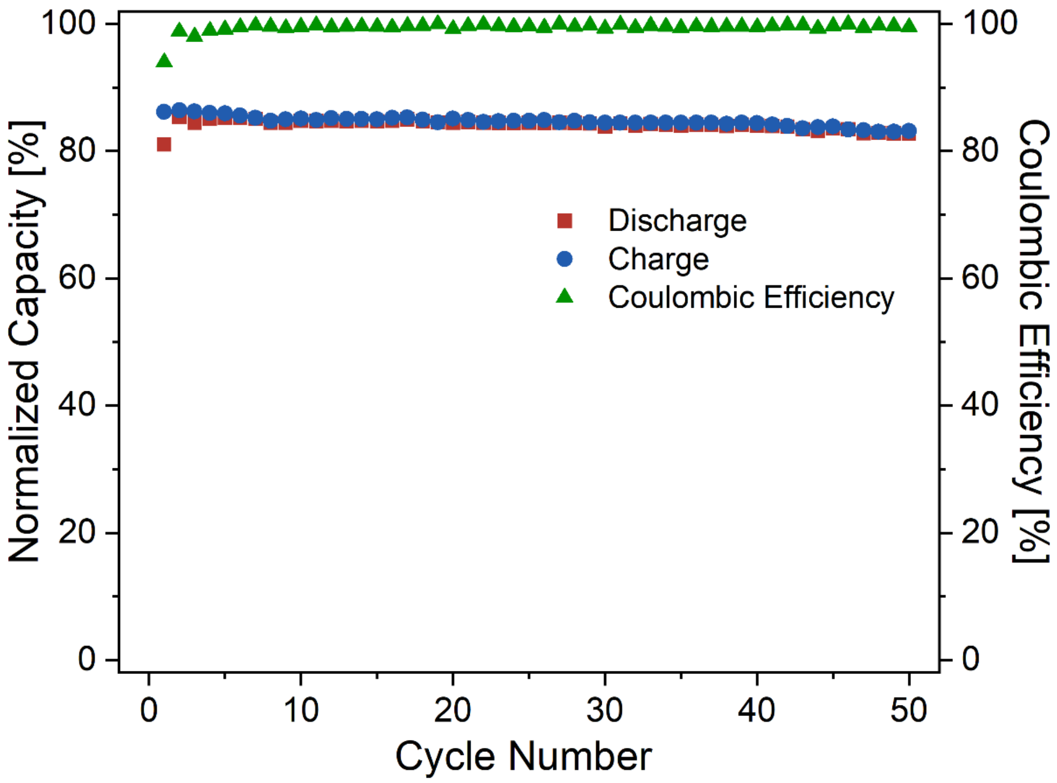

Figure S53: Normalized capacity and coulombic efficiency versus cycle number of **1-*i*Pr** with 0.1 M [(*n*-Bu)$_4$N][PF$_6$] as supporting electrolyte in MeCN:DMF (1:1, *v*:*v*) in a symmetrical H-cell experiment. The experiment was performed as described above at 0.5 mA (3C) with voltaic cutoffs of −2.10 and −1.70 V (vs internal reference) over the course of 27.3 hours, giving an average 0.11% capacity fade per hour.

H-cell of **1-*i*Pr** in MeCN:DMF (2:1, *v*:*v*)

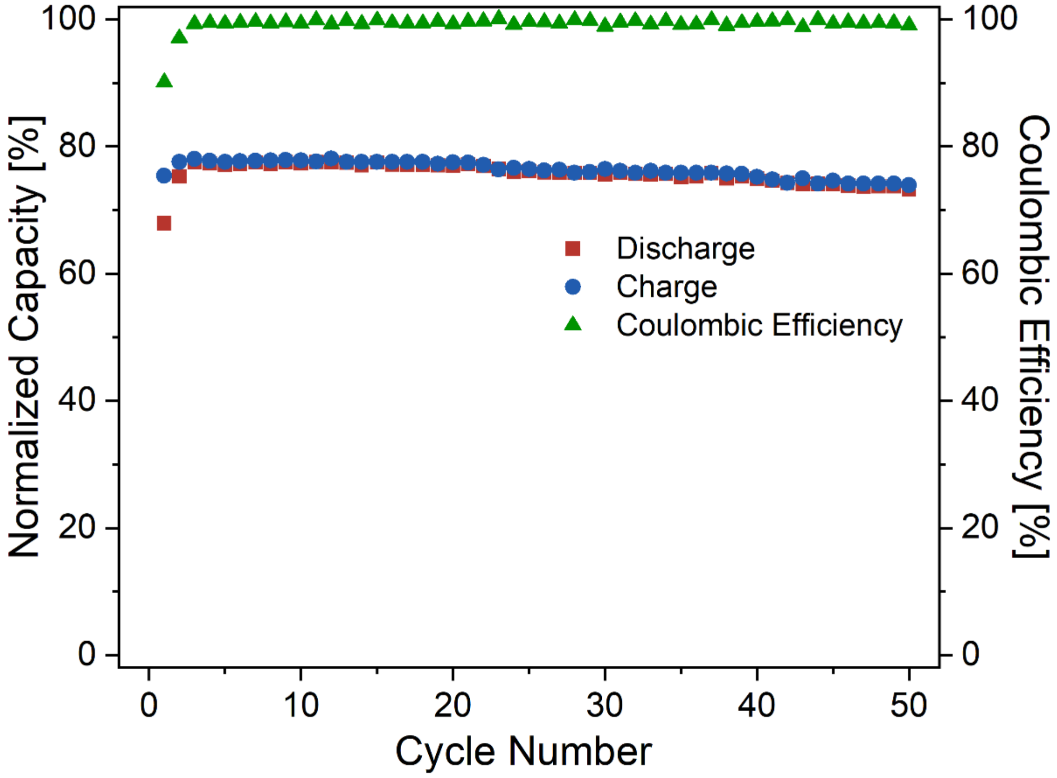

Figure S54: Normalized capacity and coulombic efficiency versus cycle number of **1-*i*Pr** with 0.1 M [(*n*-Bu)$_4$N][PF$_6$] as supporting electrolyte in MeCN:DMF (2:1, *v*:*v*) in a symmetrical H-cell experiment. The experiment was performed as described above at 0.5 mA (3C) with voltaic cutoffs of −2.10 and −1.70 V (vs internal reference) over the course of 24.5 hours, giving an average 0.22% capacity fade per hour.

C-Rate Studies

Low C-Rates

The influence of C-rate on the stability of the optimized redox system **1-*i*Pr/1-*i*Pr**$^{•-}$ was examined by preparing a symmetrical H-cell in MeCN:DMF (3:2, *v*:*v*) with 0.1 M [(*n*-Bu)$_4$N][PF$_6$] as supporting electrolyte. The cell was subjected to a sequence of galvanostatic charge-discharge cycles: five cycles each at 1C (0.168 mA), 2C (0.336 mA), 3C (0.504 mA), 4C (0.672 mA), and 5C (0.840 mA), followed by five cycles each in reverse order (4C, 3C, 2C, and 1C). The total experiment duration was 35.5 hours.

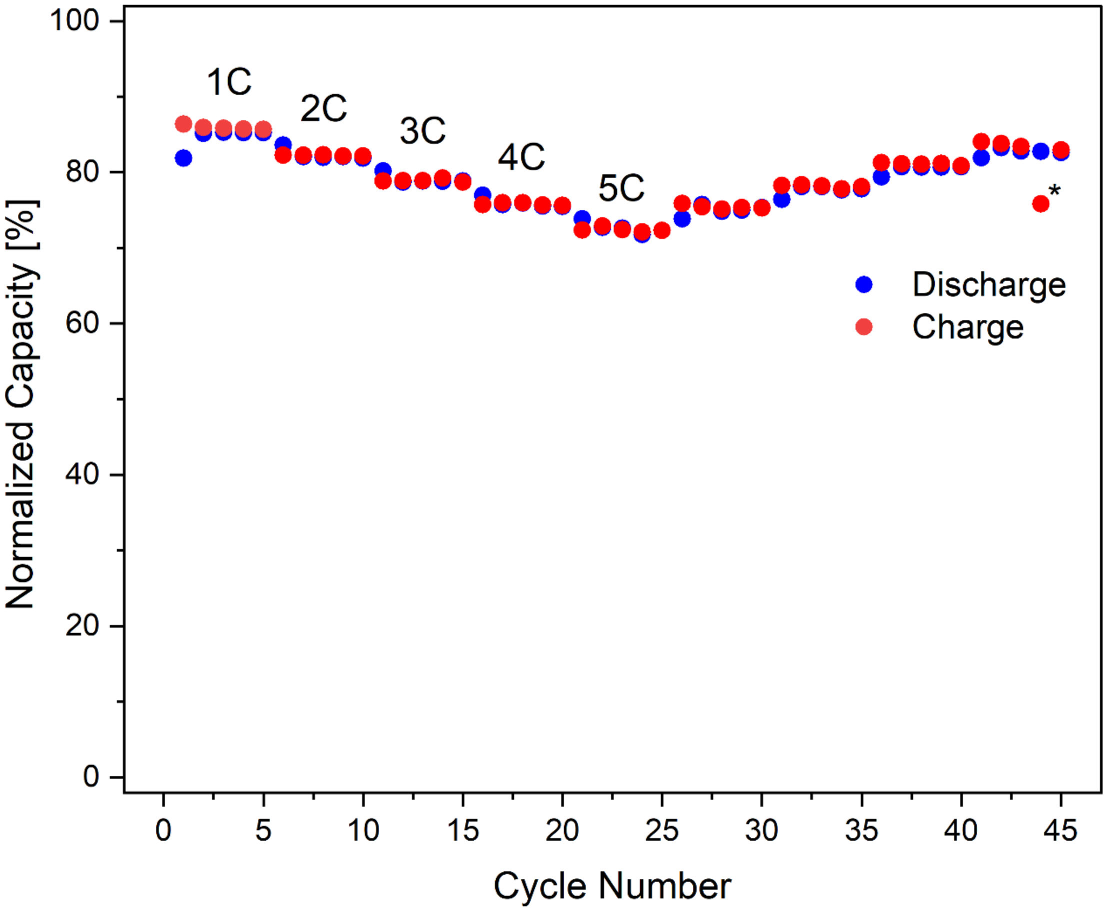

Figure S55: Normalized capacity versus cycle number at various C-rates for the symmetrical H-cell of **1-*i*Pr/1-*i*Pr**$^{•-}$ at 1C (0.168 mA), 2C (0.336 mA), 3C (0.504 mA), 4C (0.672 mA) and 5C (0.840 mA).*Instrumentation error resulting in partial data loss.

High C-Rates

The influence of C-rate on the stability of the optimized redox system **1-*i*Pr/1-*i*Pr**$^{•-}$ was examined by preparing a symmetrical H-cell in MeCN:DMF (3:2, *v*:*v*) with 0.1 M $[(n\text{-Bu})_4N][PF_6]$ as supporting electrolyte. The cell was then subjected to a sequence of galvanostatic charge-discharge cycles: five cycles each at 3C (0.50 mA), 6C (1.0 mA), 9C (1.5 mA), 12C (2.0 mA), and 15C (2.5 mA), followed by five cycles each in reverse order (12C, 9C, 6C, and 3C). The total experiment duration was 6.9 hours.

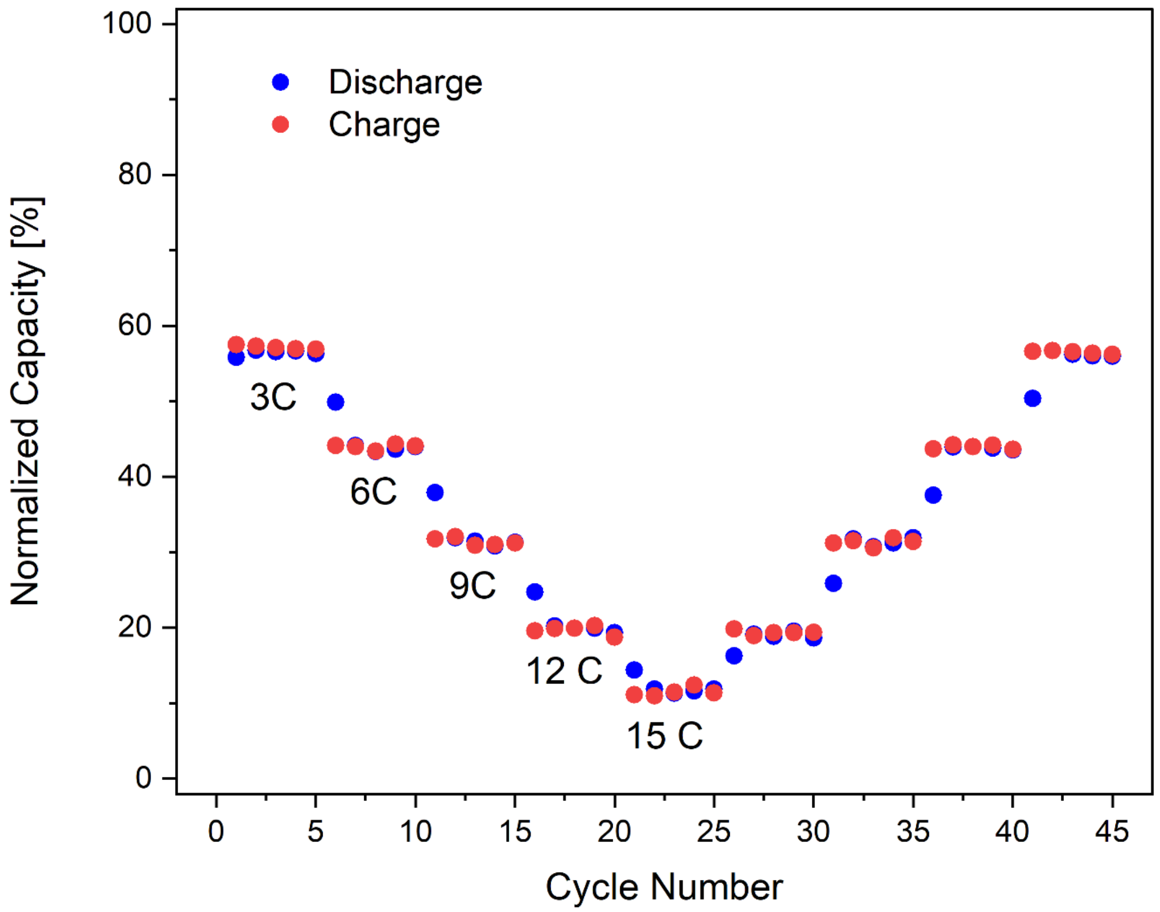

Figure S56: Normalized capacity versus cycle number at various C-rates for the symmetrical H-cell of **1-*i*Pr/1-*i*Pr**$^{•-}$ at 3C (0.50 mA), 6C (1.0 mA), 9C (1.5 mA), 12C (2.0 mA) and 15C (2.5 mA).

Anolyte Performance Comparison

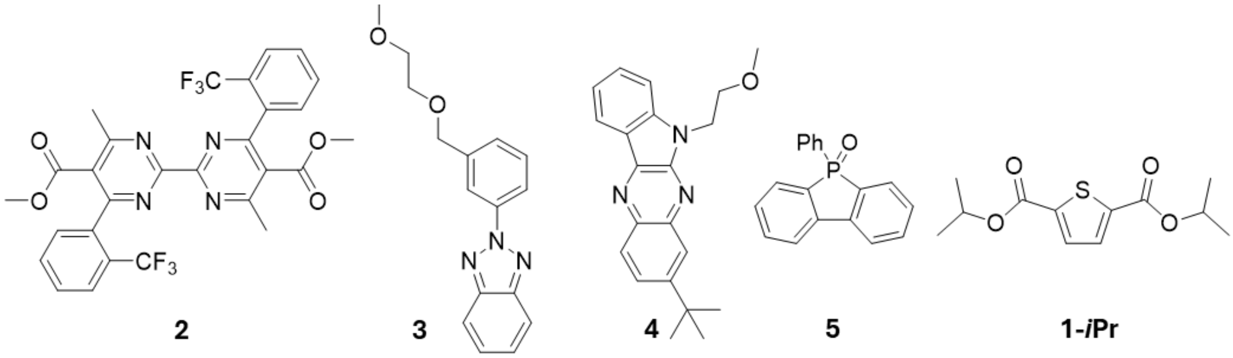

Compound	2	3	4	5	1-*i*Pr
Solvent	MeCN	DMF	MeCN	DMF	MeCN
$E_{1/2}^1$ [V vs. Fc/Fc$^+$]<br>$E_{1/2}^2$ [V vs. Fc/Fc$^+$]	-1.72<br>-1.98	-2.30<br>-	-2.01<br>-	-2.40<br>-	-2.01<br>-
Specific Weight [g/e$^-$]	295.24	283.33	333.44	276.27	256.32
Solubility of Neutral Compound	0.1 M	Miscible	> 2.7 M	1.2 M	Miscible
Solubility of Charged Compound	Miscible	>0.4 M	>0.25 M[c]	> 0.005 M[c]	> 0.5 M
Capacity Fade Rate [%/h]	0.66	0.8	0.12	n/a[b]	0.20
Theoretical Capacity [Ah/L][a]	5.36	10.72	6.70	0.13	13.4
Theoretical Energy Density [Wh/L][a]	9.92	24.66	13.47	0.32	26.94

Figure S57: Performance metrics of selected state-of-the-art organic anolytes[14–17] together with **1-*i*Pr**. [a]Calculated assuming the limiting solubility of each anolyte either in its neutral or charged state, and a half-cell voltage given by $E_{1/2}$ vs Fc/Fc$^+$. [b]The capacity fade rate (per unit time) for compound **5** could not be determined due to the use of a CCCV step during cycling. [c]Solubility of charged species was not explicitly reported, value taken from concentration of electrolyte in best performing H-cell.

Note: To allow for more direct comparison between systems studied under different conditions, metrics were chosen that correspond to the best results obtained in pure solvents in the presence of an ammonium-based supporting electrolyte. We assume that the performance of all systems can be improved by solvent mixture optimization. **1-*i*Pr** stands out due to its negatively shifted $E_{1/2}$ and high solubility both in neutral and charged states, enabling the highest theoretical capacity and energy density among the compared systems while maintaining high stability.

Magnetic Moment Determination

The magnetic moment of **[(Bz-18-c-6)K][1-*i*Pr]** was determined by the Evans method. Namely, **[(Bz-18-c-6)K][1-*i*Pr]** was carefully weighed into a scintillation vial, followed by weighing in the NMR solvent (THF-d^8). Upon complete dissolution of **[(Bz-18-c-6)K][1-*i*Pr]**, the resulting solution was transferred into a J-Young NMR tube containing a flame-sealed capillary with Fc dissolved in THF-d^8. The $\Delta\delta$ measurement for Fc was performed in a temperature-controlled NMR probe at 298 K. This procedure was repeated three times and the average of these three measurements is reported.

Table S1: Evans measurements and the corresponding effective magnetic moment.

Trial	Mass of **[(Bz-18-c-6)K][1-*i*Pr]** [mg]	Mass of solvent [g]	μ_{eff} [μB]
1	8.40	0.601	1.73
2	8.40	0.490	1.73
3	8.30	0.528	1.63

Photophysical Data

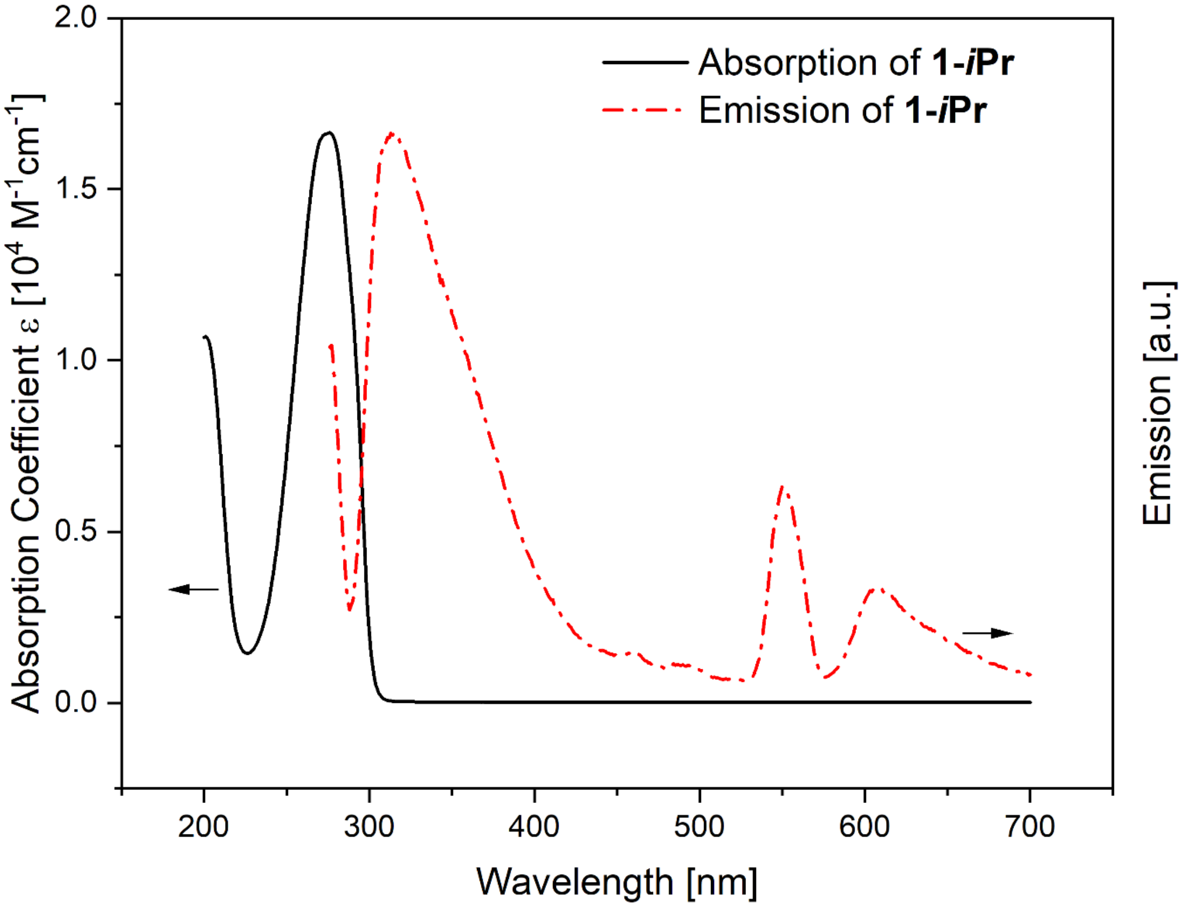

Figure S58: UV-Vis (black solid line) and emission (red dashed line) spectra of **1-*i*Pr** recorded in MeCN at room temperature.

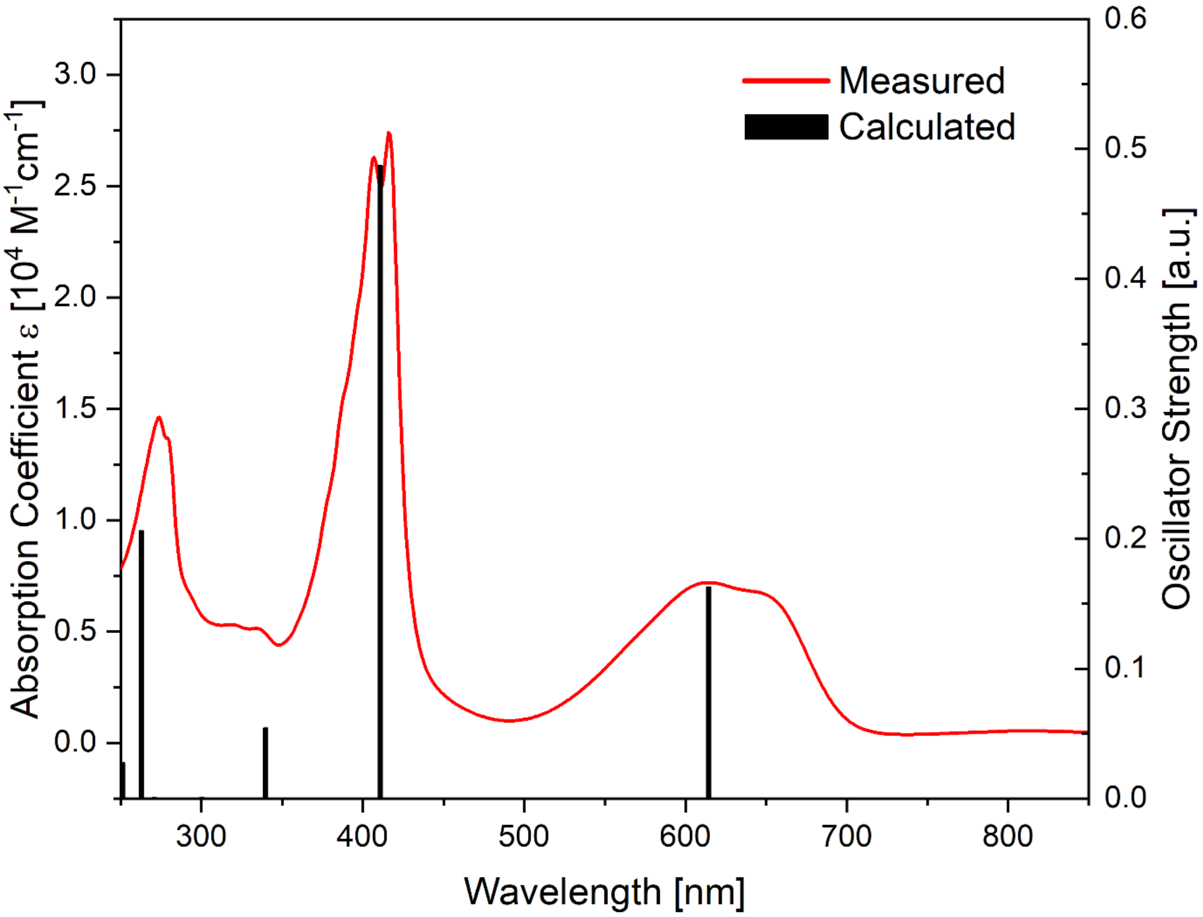

Figure S59: UV-Vis-NIR spectrum of **[(Bz-18-c-6)K][1-*i*Pr]** (red) recorded in MeCN at room temperature and the data from the TD-DFT calculations of **1-*i*Pr**$^{\bullet-}$ at the ωB97-D3/def2-TZVP-CPCM(Acetonitrile) level of theory (black).

EPR Data

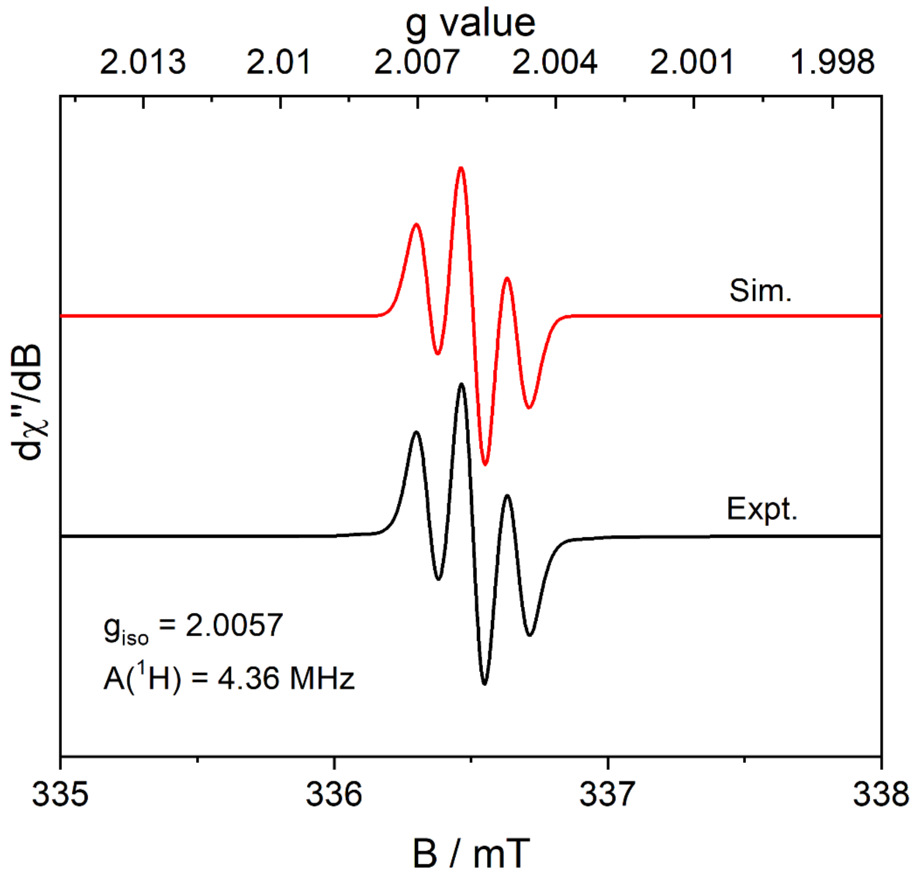

Figure S60: X-Band EPR spectrum of **[(Bz-18-c-6)K][1-*i*Pr]** in 2-methyltetrahydrofuran (2-Me-THF) at 298 K.

Fitting Parameter: g value: 2.0057; Linewidth: 0.1205; Coupling Constant 4.3620.

Acquisition parameters: Microwave frequency: 9.44717628677527 GHz, modulation: 0.05 mT, modulation frequency: 100 kHz, sweep time: 60 s, number of acquisitions: 8.

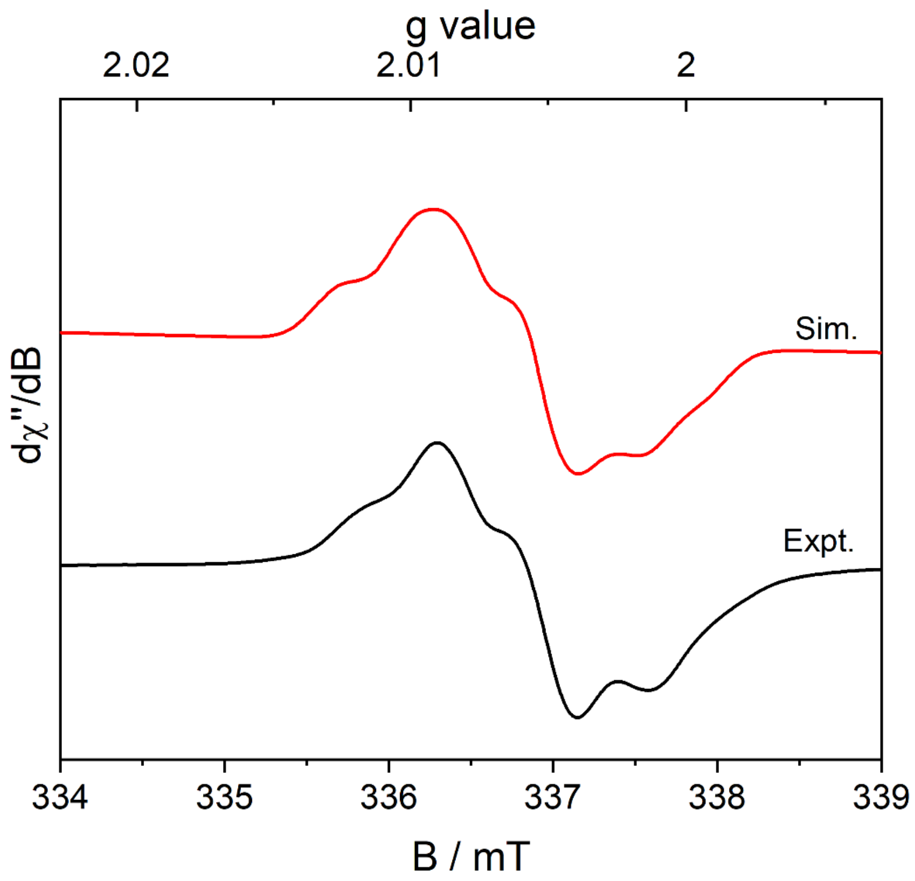

Figure S61: X-Band EPR spectrum of **[(Bz-18-c-6)K][1-*i*Pr]** in 2-methyltetrahydrofuran (2-Me-THF) glass at 90 K.

Fitting Parameter: g tensor: 2.0090 2.0013 2.0072; Linewidth: 0.3809; Coupling Constants: $A_1(^1H)$ = (20.0580 9.7210 10.3494), $A_2(^1H)$ = (11.7199 8.9505 6.5694).

Acquisition parameters: Microwave frequency: 9.45625219595552 GHz, modulation: 0.05 mT, modulation frequency: 100 kHz, sweep time: 60 s, number of acquisitions: 8.

X-Ray Data

Identification code	2456249
Empirical formula	$C_{32}H_{40}KO_{10}S$
Formula weight	655.80
Temperature/K	100.01(10)
Crystal system	monoclinic
Space group	Pn
a/Å	8.39410(10)
b/Å	13.3931(2)
c/Å	15.3065(3)
α/°	90
β/°	105.185(2)
γ/°	90
Volume/Å^3	1660.72(5)
Z	2
ρ_{calc}g/cm^3	1.311
μ/mm^{-1}	2.446
F(000)	694.0
Crystal size/mm^3	0.159 × 0.101 × 0.065
Radiation	Cu Kα (λ = 1.54184)
2Θ range for data collection/°	6.6 to 148.888
Index ranges	-8 ≤ h ≤ 9, -16 ≤ k ≤ 16, -18 ≤ l ≤ 18
Reflections collected	33496
Independent reflections	5938 [R_{int} = 0.0343, R_{sigma} = 0.0263]
Data/restraints/parameters	5938/2/402
Goodness-of-fit on F^2	1.045
Final R indexes [I>=2σ (I)]	R_1 = 0.0346, wR_2 = 0.0858
Final R indexes [all data]	R_1 = 0.0368, wR_2 = 0.0871
Largest diff. peak/hole / e Å^{-3}	0.48/-0.25
Flack parameter	0.287(11)

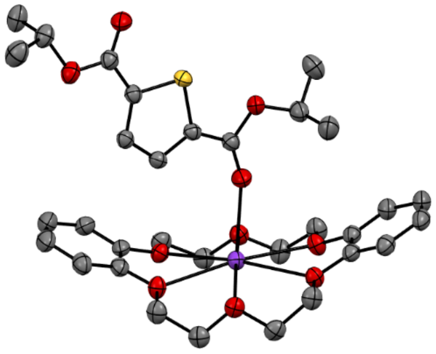

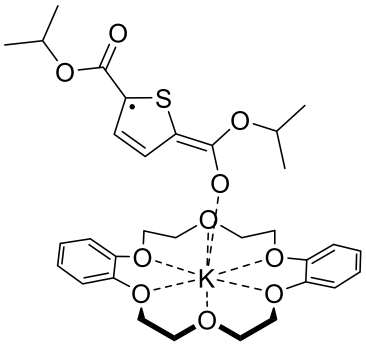

Computational Data

General Considerations

All computations were carried out using the ORCA 6.0.1 program package.[18] Orbitals and spin-density plots were visualized using Chemcraft.

Geometry optimizations were performed at the BP86/def2-TZVP level of theory using the CPCM model.[19–22] Numerical frequency calculations were carried out at the same level of theory to confirm the nature of stationary points located by geometry optimizations and to confirm zero imaginary frequencies. The RI approximation with general def2/J basis set was used to accelerate computations.

TD-DFT calculations were performed on the pre-optimized structures at the ωB97-D3 def2-TZVP level of theory using the CPCM model.[22] The RIJCOSX approximation with general def2/J basis set was used to accelerate the computations.[23] To better match the calculated UV/Vis spectrum to the experimentally obtained data, a redshift of 0.5241 eV was applied to all data points.

Sample ORCA input file for geometry optimization and frequency calculation:

```
! BP86 def2-TZVP
! RI def2/J
! Opt
! Freq
! Normalprint Printbasis PrintMOs

%pal nprocs 8 end
%maxcore 4000

*xyz "charge" "spin multiplicity"
XYZ Coordinates
*
```

Sample ORCA input file for TD-DFT calculation:

! wB97X-D3 def2-TZVP

! RIJCOSX def2/J

! CPCM(Acetonitrile)

%pal nprocs 16 end

%maxcore 8000

%tddft nroots 150

maxdim 5

 end

*xyz “charge” “spin multiplicity”

XYZ Coordinates

*

Spin Population Analysis

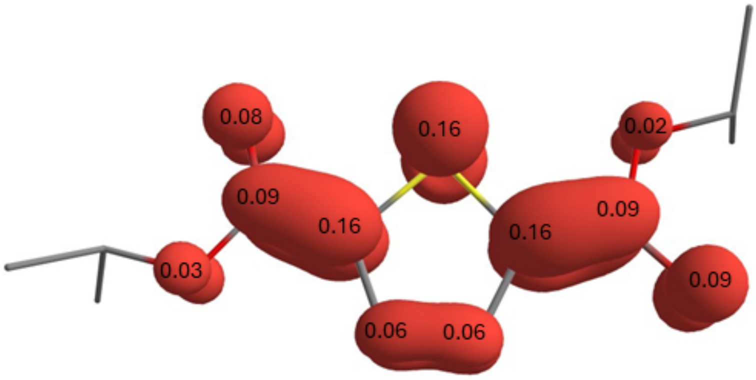

Figure S62: Representation of a spin density plot of compound **[(Bz-18-c-6)K][1-*i*Pr]** and corresponding spin population analysis.

Time-Dependent DFT Calculations

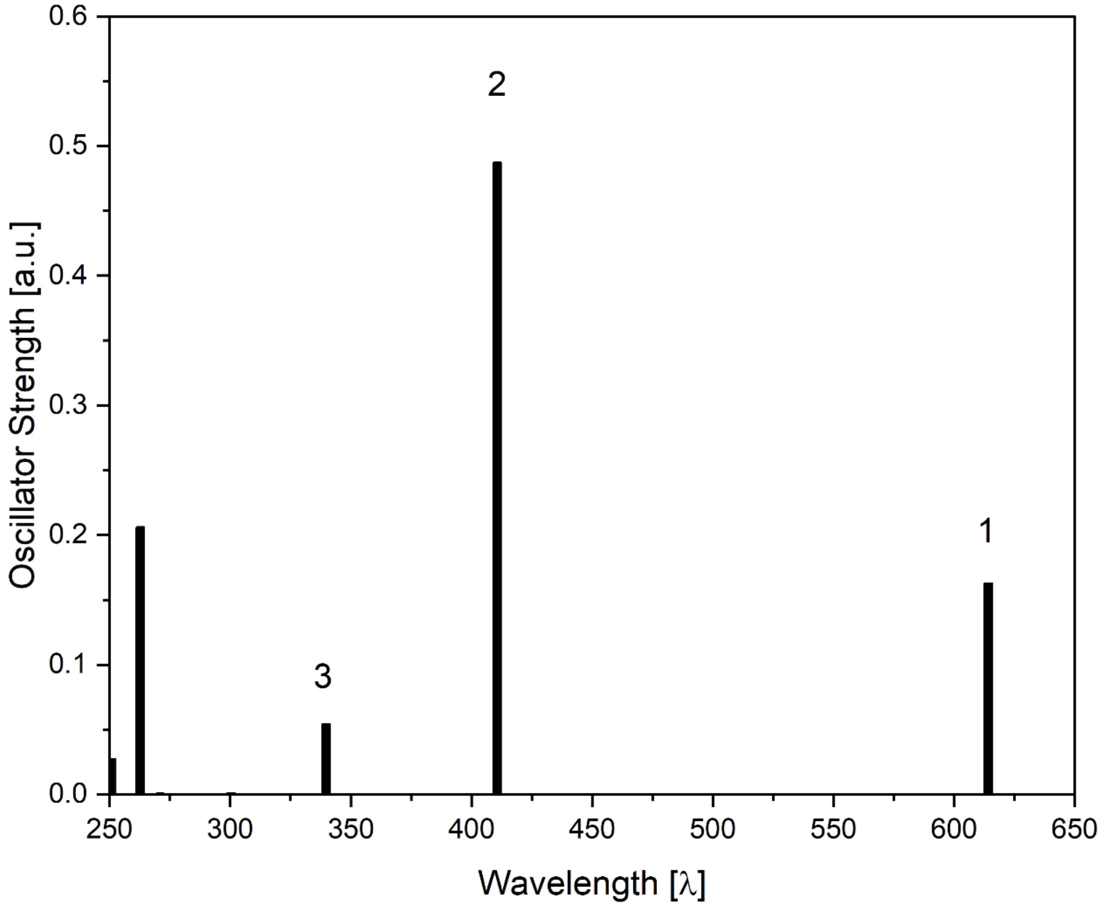

Figure S63: Data from the TD-DFT calculations of **1-*i*Pr**$^{\bullet-}$ at the ωB97-D3/def2-TZVP-CPCM(Acetonitrile) level of theory.

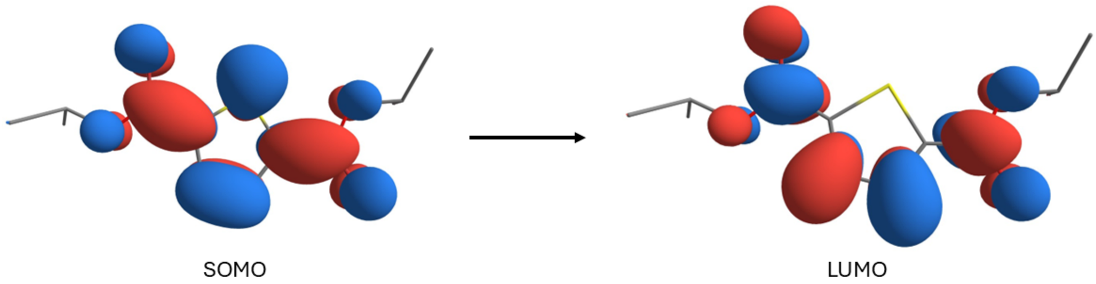

Figure S64: Molecular orbital plots of the dominant transition (90.8%) from transition #1 at 614 nm.

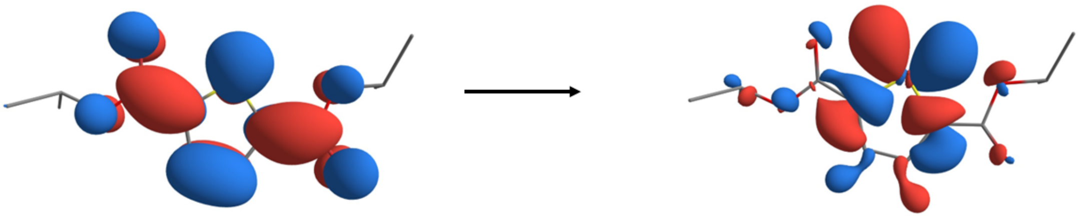

Figure S65: Molecular orbital plots of the dominant transition (91.0%) from transition #2 at 411 nm.

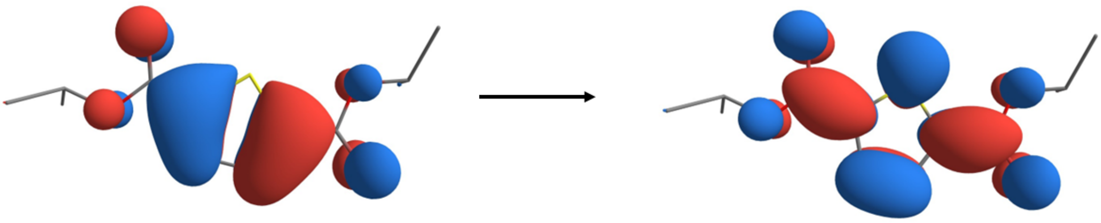

Figure S66: Molecular orbital plots of the dominant transition (98.9%) from transition #3 at 340 nm.

Coordinates

1-Me

C	0.932553	-0.209911	-0.273187
O	2.351130	-0.311806	-0.563476
C	3.004566	0.859848	-0.736306
O	2.476086	1.960875	-0.662022
C	4.434403	0.650001	-1.027045
C	5.369662	1.650791	-1.249444
C	6.658456	1.141607	-1.502415
C	6.699911	-0.245212	-1.472207
C	7.897697	-1.074056	-1.699138
O	9.008077	-0.620176	-1.940315
O	7.617812	-2.394186	-1.607178
C	8.735747	-3.296063	-1.815862
S	5.145590	-0.924057	-1.129307
H	0.586830	-1.242023	-0.166110
H	0.412383	0.289159	-1.100465
H	0.776467	0.349863	0.657507
H	5.114189	2.708768	-1.226509
H	7.537803	1.751295	-1.702650
H	8.318364	-4.300886	-1.704858
H	9.513804	-3.114293	-1.063787
H	9.150411	-3.157548	-2.822247

1-Me$^{\bullet -}$

C	0.932553	-0.209911	-0.273187
O	2.351130	-0.311806	-0.563476
C	3.004566	0.859848	-0.736306
O	2.476086	1.960875	-0.662022
C	4.434403	0.650001	-1.027045
C	5.369662	1.650791	-1.249444
C	6.658456	1.141607	-1.502415
C	6.699911	-0.245212	-1.472207
C	7.897697	-1.074056	-1.699138
O	9.008077	-0.620176	-1.940315
O	7.617812	-2.394186	-1.607178
C	8.735747	-3.296063	-1.815862
S	5.145590	-0.924057	-1.129307
H	0.586830	-1.242023	-0.166110
H	0.412383	0.289159	-1.100465
H	0.776467	0.349863	0.657507
H	5.114189	2.708768	-1.226509
H	7.537803	1.751295	-1.702650
H	8.318364	-4.300886	-1.704858
H	9.513804	-3.114293	-1.063787
H	9.150411	-3.157548	-2.822247

1-*i*Pr

C	1.117149	0.077859	-0.277240
C	2.632123	0.097816	-0.406843
C	3.127026	0.293651	-1.831197
O	3.125168	1.244961	0.389566
C	3.953867	1.000401	1.423118
O	4.346775	-0.108008	1.776252
C	4.348575	2.248855	2.103578
C	3.990824	3.562096	1.831173
C	4.586362	4.478333	2.721333
C	5.393852	3.858481	3.664689
C	6.153849	4.541455	4.730225
O	6.146576	5.756607	4.887207
O	6.847163	3.663963	5.486130
C	7.657514	4.200442	6.602419
C	6.792148	4.262956	7.852786
C	8.856612	3.276998	6.732366
S	5.419245	2.141288	3.456636
H	0.713087	-0.765376	-0.855376
H	0.677668	1.006991	-0.668499
H	0.812349	-0.045953	0.770922
H	3.069474	-0.807417	0.033658
H	2.734833	1.228631	-2.257248
H	2.780940	-0.542063	-2.456011
H	4.224703	0.318945	-1.867824
H	3.323538	3.838832	1.017313
H	4.442444	5.557113	2.689852
H	7.974019	5.209988	6.306037
H	6.439281	3.259117	8.130464
H	5.923819	4.918283	7.702821
H	7.384620	4.664681	8.687228
H	8.539465	2.254456	6.983820
H	9.510864	3.642008	7.536502
H	9.437689	3.250249	5.800610

1-*i*Pr$^{\bullet -}$

C	1.100555	0.066532	-0.280453
C	2.618526	0.120353	-0.412432
C	3.089679	0.289484	-1.852427
O	3.098799	1.263952	0.357210
C	3.961270	1.018692	1.419712

O	4.331845	-0.133152	1.733052
C	4.354440	2.218701	2.083487
C	4.013745	3.569945	1.829103
C	4.605953	4.472076	2.702460
C	5.436020	3.870037	3.679832
C	6.172181	4.535784	4.706175
O	6.196446	5.768688	4.902546
O	6.897064	3.645647	5.491129
C	7.655267	4.197003	6.609079
C	6.773424	4.243629	7.853484
C	8.882152	3.311802	6.780411
S	5.451622	2.112276	3.466458
H	0.702595	-0.785463	-0.851122
H	0.641094	0.986972	-0.671602
H	0.802657	-0.054255	0.770624
H	3.067093	-0.781357	0.027537
H	2.688271	1.216861	-2.288558

H	2.742832	-0.556011	-2.464628
H	4.187036	0.324241	-1.904466
H	3.346144	3.856175	1.017289
H	4.460510	5.551511	2.659712
H	7.956769	5.217924	6.332141
H	6.436121	3.232189	8.125935
H	5.891710	4.876880	7.685097
H	7.338026	4.659396	8.701183
H	8.589109	2.278443	7.019823
H	9.504524	3.691063	7.603652
H	9.490142	3.300621	5.865029

References

(1) C. A. Henrick, G. B. Staal, Pyridine Esters of Cyclopropane-Carboxylic Acid. US4093622A, June 6, 1978.

(2) R. Chesworth, L. D. Gegnas, Benzimidazole Compounds and Their Use as Estrogen Agonists/Antagonists. US2004002524A1, January 1, 2004.

(3) M. Okada, K. Hasumi, T. Nishimoto, I. Miwa, T. Aotsuka, H. Kanazawa, Heterocyclic Ring Compound and H1 Receptor Antagonist. WO2011148888A1, December 1, 2011. https://patents.google.com/patent/WO2011148888A1/en (accessed 2025-04-04).

(4) Y. Wang, Y. Gao, L. Wang, J. Liu, Hydrolysis-Resistant High-Barrier Biodegradable Film and Preparation Method Thereof. CN119192548A, December 27, 2024.

(5) G. Knorr, M. L. Bossi, A. N. Butkevich, S. W. Hell, Synthesis of Thioxanthone 10,10-Dioxides and Sulfone-Fluoresceins via Pd-Catalyzed Sulfonylative Homocoupling. *Org. Lett.* **2024**, *26* (4), 945–949. https://doi.org/10.1021/acs.orglett.3c04300.

(6) W. Chen, J. Ning, Y. Sun, G. Zhou, I. Murtaza, A. Shuja, Y. He, I. F. Perepichka, H. Meng, Thiophene-2,5-Diesters as Electrochromic Materials: The Effect of Ester Groups on the Device Performance and Stability. *Org. Electron.* **2021**, *96*, 106188. https://doi.org/10.1016/j.orgel.2021.106188.

(7) S. Stoll, A. Schweiger, EasySpin, a Comprehensive Software Package for Spectral Simulation and Analysis in EPR. *J. Magn. Reson.* **2006**, *178* (1), 42–55. https://doi.org/10.1016/j.jmr.2005.08.013.

(8) O. V. Dolomanov, L. J. Bourhis, R. J. Gildea, J. A. K. Howard, H. Puschmann, OLEX2: A Complete Structure Solution, Refinement and Analysis Program. *J. Appl. Crystallogr.* **2009**, *42* (2), 339–341. https://doi.org/10.1107/S0021889808042726.

(9) G. M. Sheldrick, SHELXT – Integrated Space-Group and Crystal-Structure Determination. *Acta Crystallogr. Sect. Found. Adv.* **2015**, *71* (1), 3–8. https://doi.org/10.1107/S2053273314026370.

(10) Z. Yan, R. J. Wycisk, A. S. Metlay, L. Xiao, Y. Yoon, P. N. Pintauro, T. E. Mallouk, High-Voltage Aqueous Redox Flow Batteries Enabled by Catalyzed Water Dissociation and Acid–Base Neutralization in Bipolar Membranes. *ACS Cent. Sci.* **2021**, *7* (6), 1028–1035. https://doi.org/10.1021/acscentsci.1c00217.

(11) M.-A. Goulet, M. J. Aziz, Flow Battery Molecular Reactant Stability Determined by Symmetric Cell Cycling Methods. *J. Electrochem. Soc.* **2018**, *165* (7), A1466. https://doi.org/10.1149/2.0891807jes.

(12) Y. Shi, Z. Li, Q. Mai, Preparation Method of 2,5-Thiophenedicarboxylic Acid. CN105906606A, August 31, 2016.

(13) R. L. Myers, *The 100 Most Important Chemical Compounds: A Reference Guide*; Bloomsbury Academic, 2007.

(14) J. D. Griffin, A. R. Pancoast, M. S. Sigman, Interrogation of 2,2'-Bipyrimidines as Low-Potential Two-Electron Electrolytes. *J. Am. Chem. Soc.* **2021**, *143* (2), 992–1004. https://doi.org/10.1021/jacs.0c11267.

(15) Y. Yan, L. Zhang, R. Walser-Kuntz, D. B. Vogt, M. S. Sigman, G. Yu, M. S. Sanford, Benzotriazoles as Low-Potential Anolytes for Non-Aqueous Redox Flow Batteries. *Chem. Mater.* **2022**, *34* (23), 10594–10605. https://doi.org/10.1021/acs.chemmater.2c02682.

(16) W. Zhang, R. Walser-Kuntz, J. S. Tracy, T. K. Schramm, J. Shee, M. Head-Gordon, G. Chen, B. A. Helms, M. S. Sanford, F. D. Toste, Indolo[2,3-b]Quinoxaline as a Low Reduction Potential and High Stability Anolyte Scaffold for Nonaqueous Redox Flow Batteries. *J. Am. Chem. Soc.* **2023**, *145* (34), 18877–18887. https://doi.org/10.1021/jacs.3c05210.

(17) E. R. Mahoney, M. Boudjelel, H. Shavel, M. D. Krzyaniak, M. R. Wasielewski, C. A. Malapit, Triphenylphosphine Oxide-Derived Anolyte for Application in Nonaqueous Redox Flow Battery. *J. Am. Chem. Soc.* **2025**, *147* (2), 1381–1386. https://doi.org/10.1021/jacs.4c07750.

(18) F. Neese, Software Update: The ORCA Program System—Version 5.0. *WIREs Comput. Mol. Sci.* **2022**, *12* (5), e1606. https://doi.org/10.1002/wcms.1606.

(19) A. D. Becke, Density-Functional Exchange-Energy Approximation with Correct Asymptotic Behavior. *Phys. Rev. A* **1988**, *38* (6), 3098–3100. https://doi.org/10.1103/PhysRevA.38.3098.

(20) J. P. Perdew, Density-Functional Approximation for the Correlation Energy of the Inhomogeneous Electron Gas. *Phys. Rev. B* **1986**, *33* (12), 8822–8824. https://doi.org/10.1103/PhysRevB.33.8822.

(21) M. Bursch, J.-M. Mewes, A. Hansen, S. Grimme, Best-Practice DFT Protocols for Basic Molecular Computational Chemistry. *Angew. Chem. Int. Ed.* **2022**, *61* (42), e202205735. https://doi.org/10.1002/anie.202205735.

(22) V. Barone, M. Cossi, Quantum Calculation of Molecular Energies and Energy Gradients in Solution by a Conductor Solvent Model. *J. Phys. Chem. A* **1998**, *102* (11), 1995–2001. https://doi.org/10.1021/jp9716997.

(23) S. Kossmann, F. Neese, Efficient Structure Optimization with Second-Order Many-Body Perturbation Theory: The RIJCOSX-MP2 Method. *J. Chem. Theory Comput.* **2010**, *6* (8), 2325–2338. https://doi.org/10.1021/ct100199k.
